# Lead optimisation of OXS007417: *in vivo* PK profile and hERG liability modulation to optimise a small molecule differentiation agent for the potential treatment of acute myeloid leukaemia†

**DOI:** 10.1039/d4md00275j

**Published:** 2024-07-22

**Authors:** Thomas J. Cogswell, Laia Josa-Culleré, David Zimmer, Sébastien R. G. Galan, Morgan Jay-Smith, Kate S. Harris, Carole J. R. Bataille, Thomas R. Jackson, Douzi Zhang, Stephen G. Davies, Paresh Vyas, Thomas A. Milne, Graham M. Wynne, Angela J. Russell

**Affiliations:** a Department of Chemistry, Chemistry Research Laboratory, University of Oxford Mansfield Road Oxford OX1 3TA UK angela.russell@chem.ox.ac.uk; b MRC Molecular Haematology Unit, MRC Weatherall Institute of Molecular Medicine, Radcliffe Department of Medicine, University of Oxford Oxford OX3 9DS UK; c Department of Pharmacology, University of Oxford Mansfield Road Oxford OX1 3QT UK

## Abstract

The development of a safe, efficacious, and widely effective differentiation therapy for AML would dramatically improve the outlook for many patients worldwide. To this aim, our laboratory has discovered a class of differentiation agents that demonstrate tumour regression in murine models *in vivo*. Herein, we report a lead optimisation process around compound OXS007417, which led to improved potency, solubility, metabolic stability, and off-target toxicity of this compound class. A hERG liability was investigated and successfully alleviated through addition of nitrogen atoms into key positions of the compound. OXS008255 and OXS008474 demonstrated an improved murine PK profile in respect to OXS007417 and a delay in tumour growth in a subcutaneous *in vivo* model using HL-60 cells.

## Abstract 2

The optimisation of a class of AML differentiation agents is described to show improved potency, solubility and stability, reduced off target toxicity, and tumour regression in a murine model *in vivo*.

## Graphical abstract

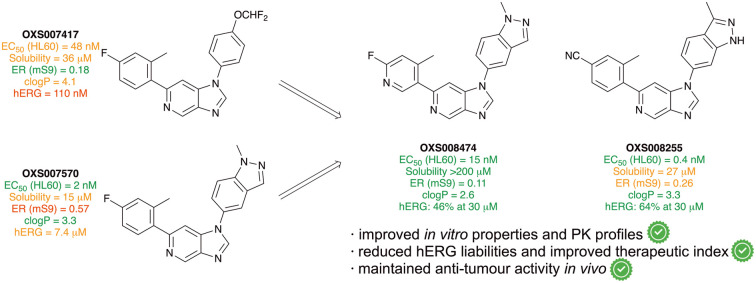

## 1. Introduction

Acute myeloid leukaemia (AML) is a cancer of the haematopoietic system characterised by its lethality and heterogeneity.^1^ It represents an area of significant unmet clinical need which is especially clear in older patients – studies indicate a 5 year survival rate lower than 11% in people over 60.^2^ The disease stems from a disruption of the normal differentiation pathway of the myeloid lineage of blood cells, which leads to the build-up of abnormal myeloid blasts and a dramatic reduction in normal blood cell production. There are a wide variety of genetic mutations that lead to this disruption and patients are organised into disease subtypes for more effective treatment.^3,4^ The WHO subtype classification is not only dependant on the specific mutation(s) but also on several parameters including cancerous cell morphology and clinical history.^4^

The heterogeneity of AML makes the disease especially difficult to tackle with a single therapy. The “best-in-class” therapies involve intensive chemotherapy, focusing on the reduction of tumour burden and removal of the abnormal blasts. Chemotherapeutic agents such as cytarabine are the first port of call for AML treatment, often used in combination with anthracycline drugs such as daunorubicin or idarubicin; they are very effective for the removal of AML blasts and tumour regression. However, these treatment regimens are highly toxic and many patients, especially the elderly, are not able to tolerate them. Even after successful initial treatment, patients have a strong tendency to relapse within 2 years of diagnosis, often with resistance to further cytarabine therapy. Therefore, there is a strong clinical need for novel therapies to tackle AML in a curative manner without the dose-related toxicity and relapse rates that limit the current treatment options. In recent years, several inhibitors targeting specific mutations including IDH1/2,^5^ FLT3,^6^ and LSD1^7^ are delivering exciting breakthroughs – some have reached the clinic and others are in a late stage of development.^8^ However, only a small subset of patients with the corresponding mutational fingerprint will respond to these treatments.

Acute promyelocytic leukaemia (APL) is a subset of AML where there is a block in the differentiation of granulocytes leading to a build-up of abnormal promyelocytes.^9,10^ Unlike AML, patients with APL have a good prognosis with a 10 year survival rate of 80–90%. This is due to APL being uniquely sensitive to the differentiating agent all-*trans* retinoic acid (ATRA) in combination with a chemotherapy agent, arsenic trioxide. Unfortunately, ATRA does not promote differentiation in other AML subtypes. However, there is significant interest in utilising a similar approach for AML and the discovery of new cellular mechanisms that could be harnessed to find a differentiation agent effective in other AML cells. Overcoming the differentiation block in AML, as seen in APL, could provide a novel low toxicity treatment with the potential to transform the prognosis for AML patients.^11^

We have previously reported the discovery of a small molecule differentiation agent, OXS007417.^12^ This compound, which was discovered through a phenotypic screening approach followed by hit optimisation of potency and other properties, causes differentiation in a variety of AML cell lines at low nanomolar concentrations. Evaluation using *in vivo* experiments encompassing both subcutaneous and orthotopic murine models, OXS007417 demonstrated significant disease specific tumour regression. Follow-up mechanistic studies highlighted the major driver of the phenotypic effect to be binding of the compound to tubulin beta chain,^13^ a common target in the field of oncology. The anti-proliferative effects of binding to β-tubulin are well documented, however the differentiation phenotype is previously unknown for tubulin binders. As ATRA is known to cause an anti-proliferative effect combined with cell differentiation in APL cells, a similar combined effect in AML cell lines is an exciting prospect.

**Fig. 1:**
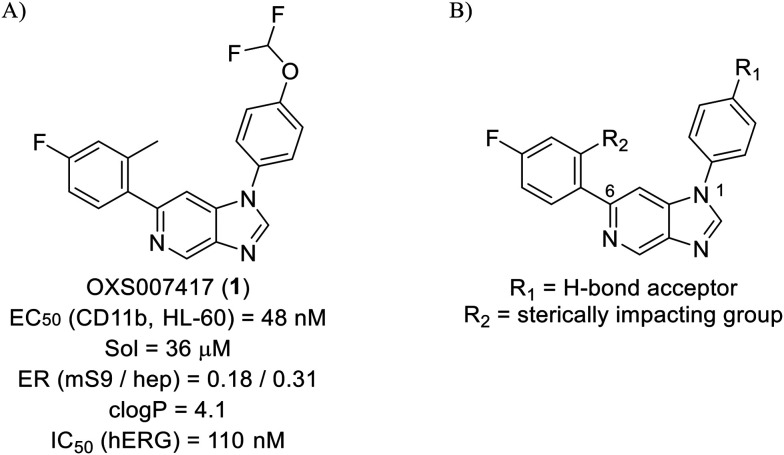
(A) Structure of OXS007417, and (B) major pharmacophore of the previously described imidazo[4,5-*c*]pyridines.

Whilst the lead molecule OXS0007417 had previously demonstrated a suitable balance of activity and metabolic stability that allowed the key murine *in vivo* proof-of-concept experiments to be undertaken successfully, some of its properties needed to be optimised to move the program forward (Fig. 1A). Despite high potency *in vitro* (EC_50_ = 48 nM) and a suitable PK profile, the clog *P* of 4.1 was higher than desired, and it had moderate solubility (36 μM) and suboptimal metabolic stability (ER = 0.18). Most critically, OXS0007417 was later found to have strong affinity to the human ether-a-go-go related gene (hERG) channel with an IC_50_ of 110 nM, which clearly had to be resolved in order to move the series forward.

The hERG channel contains multiple binding pockets that can accommodate a broad range of small molecules. Typical medicinal chemistry strategies to lower the hERG activity focus on modulating lipophilicity (TPSA, log *D*, log *P*) and altering the p*K*_a_ of basic nitrogen sites, thus lowering the compounds' lipophilicity and modulating aromatic sites. These strategies aim to reduce π-cation, hydrophobic, and π-stacking interactions to the aromatic residues present in the hERG cavity.^14–16^ There are numerous *in silico* tools in development for modelling binding to hERG,^17–19^ although to date there is no consensus on the binding mode for compounds to hERG. A wealth of literature examples of medicinal chemistry programs shows how a hERG liability has been resolved to a satisfactory therapeutic window,^20,21^ however often multiple strategies are needed to be investigated and an extensive program of SAR can be required to alleviate this interaction.

Here, we show how we were able to alleviate the hERG activity for the series and concurrently improve *in vitro* activity and metabolic stability to provide novel compounds with potential for the treatment of AML. Our general strategy consisted of improving the therapeutic window by both reducing the hERG liability and increasing potency, while improving physicochemical properties. This work builds upon the structure–activity relationship (SAR) studies previously undertaken,^12,22^ where the major pharmacophore was identified to have a H-bond acceptor in the *para* position of the aromatic ring at N-1 (Fig. 1B). This moiety can take the form of a variety of groups such as –OMe, –OCF_2_H, and heterocycles with an appropriately oriented H-bond acceptor. A further important site of the compound is the aromatic ring at C-6, which seemingly requires a group in the *ortho* position such as a –CH_3_ or –OCH_3_ to induce the correct torsion angle to this aromatic ring for significant potency. The 6,5 fused core of the molecule, which is an imidazopyridine in OXS007417, is interchangeable with other 6,5 fused heterocycles that hold the N-1 and C-6 substituents in similar geometries, such as an imidazo[1,2-*a*]pyridine,^22^ an imidazo[1,2-*a*]pyrazine, or a triazolo[4,3-*a*]pyridine.

## 2. Results

### 2.1. Initial SAR studies using OXS007570 as a starting point

We initiated our optimisation studies by focusing on an earlier compound, OXS007570, which replaced the phenyl *p*-OCF_2_H group with an *N*-methyl indazole (Table 1). This was inspired by a publication from Crawford, Dossetter, and co-workers detailing 1,3-dimethyl-1*H*-indazole as a dimethoxy bioisostere.^23^ The compound was highly active with an IC_50_ of 2 nM compared to the 48 nM of OXS0007417. Importantly, the hERG profile also improved with a 70-fold lower affinity. However, the metabolic stability and solubility of this compound were comparably worse (ER = 0.57, *S* = 15 μM) than OXS007417 and for this reason it had previously not been progressed further.

**Table 1:** Structure–activity relationships at C-6

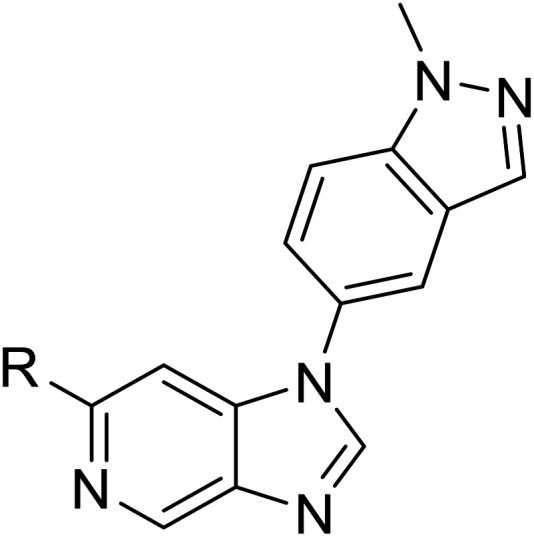								
Compound	R	EC_50_^a^ [nM]	ER in mS9^b^	Solubility^c^ [μM]	hERG^d^		clog *P*^f^	LLE^g^
					% inh^e^	IC_50_ [μM]		
OXS007570 (6)	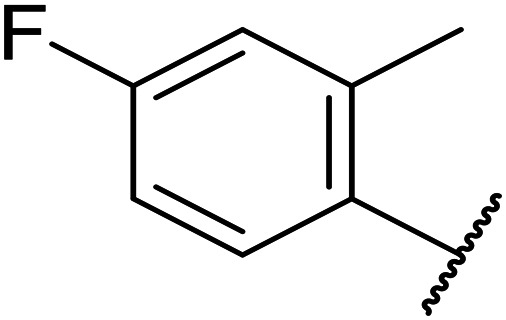	2	0.57	15	n.d.^h^	7.4	3.3	5.4
7	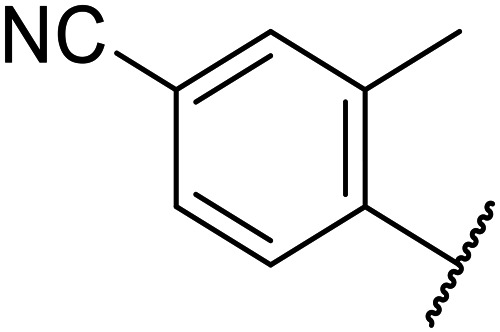	13	0.10	26	n.d.	7.3	3.0	4.9
OXS008474 (8)	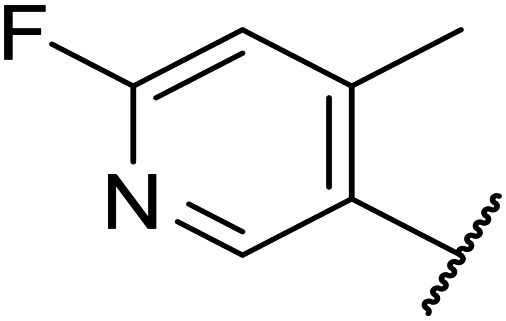	15	0.11	>200	46%	n.d.	2.6	5.2
9	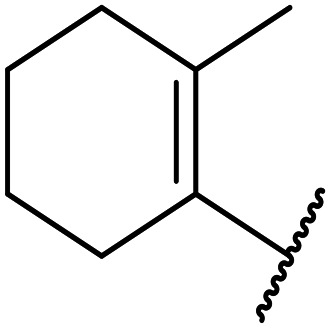	20	1	179	89%	n.d.	3.0	4.7
10	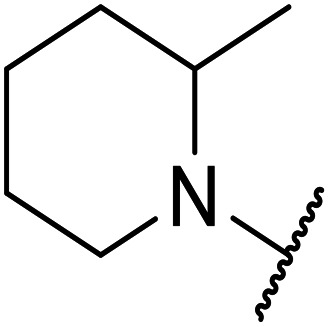	1100	n.d.	n.d.	n.d.		2.6	3.4
11	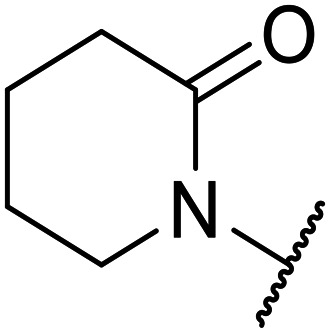	>30 000	n.d.	n.d.	n.d.		1.7	n.a.^i^

a %CD11b response in HL-60 cells.

b Extraction ratio (ER) = Cl_int_/species flow rate (mice: 90 ml min^−1^ kg^−1^) in mouse S9 fraction (mS9), high (>0.7), intermediate (0.3–0.7) or low (<0.3).

c Semi-thermodynamic aqueous solubility.

d hERG inhibition, determined using a patch clamp assay.

e Percentage inhibition of the hERG channel at 30 μM.

f Calculated octanol–water partition coefficient clog *P* determined using Datawarrior.

g Lipophilic efficiency LLE = −log EC_50_ – clog *P*.

h Not determined.

i Not applicable.

**Scheme 1:**
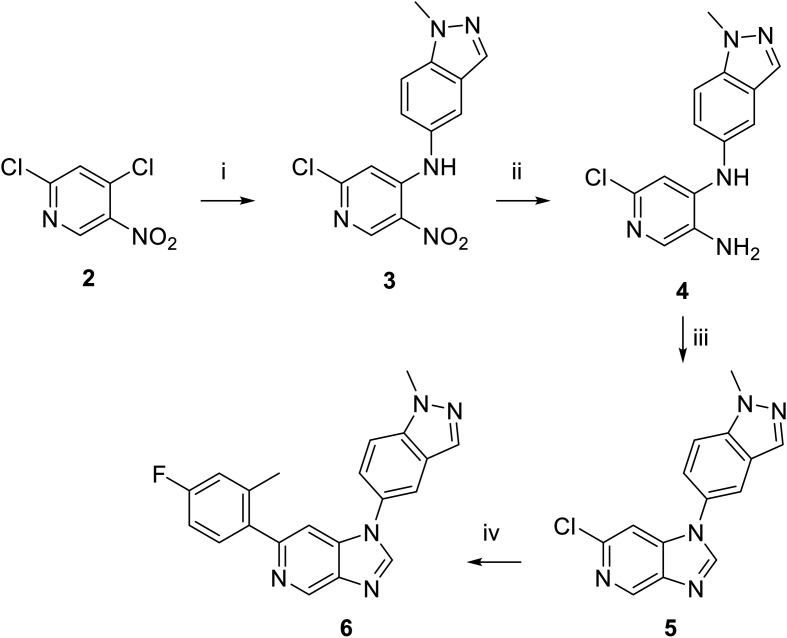
Synthetic route of OXS007570 (6). i. 1-methyl-1*H*-indazol-5-amine, Et_3_N, MeCN, rt, 48 h, 90%; ii. Fe, NH_4_Cl, EtOH/H_2_O, 78 °C, 14 h, 78%; iii. (MeO)_3_CH, HCO_2_H, 100 °C, 18 h, 84%; iv. (4-F,-2-Me)PhB(OH)_2_, K_2_CO_3_, Pd(dppf)Cl_2_, DME, 80 °C, 18 h, 11%.

Moving forwards, OXS0007570 provided a suitable starting point for future investigation with the inherent high potency and lower hERG activity. Firstly, a screen of C-6 ring systems was conducted (Table 1), keeping the indazole at the N-1 position, with the aim of improving the metabolic stability and solubility while maintaining the high AML differentiation activity and low hERG affinity. The compounds were prepared as previously described^12,24^ which is exemplified for OXS007570 in Scheme 1; for details of all compounds, see ESI.†

To evaluate the biological activity of the new derivatives, we used an *in vitro* phenotypic differentiation assay as described in the prior publications^12,13,25^ – it is based on a flow cytometry readout of HL-60 cells stained for the differentiation marker CD11b. The metabolic stability of the analogues was assessed using mouse liver S9 (mS9) fractions and is quoted as the extraction ratio (ER), which is calculated as the measured intrinsic clearance over the hepatic blood flow of each species (90 mL min^−1^ kg^−1^ for mice^26^). It has the advantage over other parameters, such as half-life and clearance, that it is independent of the species used. We assessed activity on the hERG potassium channel using a patch clamp assay,^27^ either determining the IC_50_ or the % inhibition at 30 μM.^16^

Replacement of the *p*-F 6 (OXS007570) with a *p*-CN in 7 was previously reported in these systems to increase metabolic stability, and as expected, an improvement from ER = 0.57 to ER = 0.10 was observed, although the hERG activity was unchanged and there was a 6-fold drop in potency. These trends were also observed for the pairs 1*vs.*12 and 13*vs.*14 (Table 2), and in the former case the cyano group led to an increase in hERG activity. Introduction of nitrogen atoms into the aromatic ring to reduce clog *P* and change the ring electronics, in 6-fluoro-4-methylpyridin-3-yl derivative 8, improved both the metabolic stability and hERG activity showing the first signs that site selective nitrogen inclusion could reduce hERG binding to acceptable levels. Several aliphatic rings were incorporated and tested, with the *o*-Me cyclohexene 9 showing good activity, improved solubility, but poor metabolic stability. Finally, two examples with a nitrogen linker atom were made, 2-methylpiperidine 10 and δ-lactam 11, however the potency was poor in both examples, and they were not progressed further. These were prepared using Buchwald–Hartwig and Chan–Lam coupling strategies respectively in the last step.

**Table 2:** Structure–activity relationships at N-1

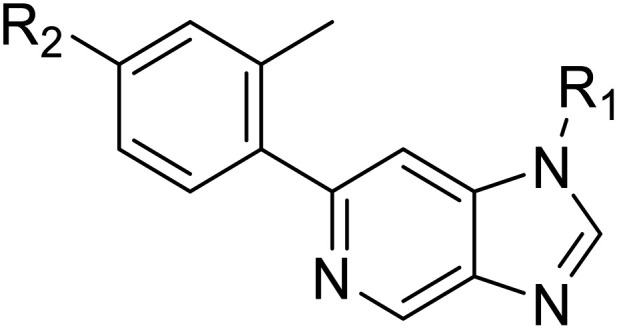									
Compound	R_1_	R_2_	EC_50_^a^ [nM]	ER in mS9^b^	Solubility^c^ [μM]	hERG^d^		clog *P*^f^	LLE^g^
						% inh^e^	IC_50_ [μM]		
OXS007417 (1)	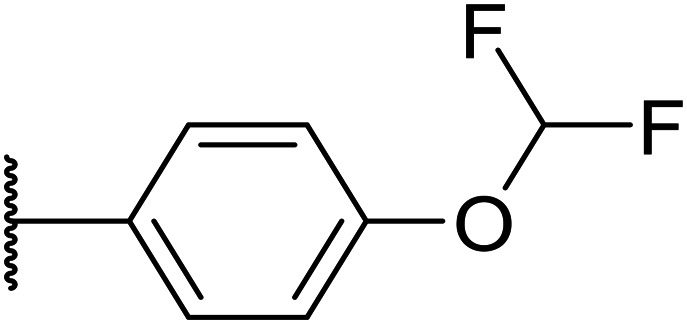	F	48	0.18	36	n.d.^h^	0.11	4.1	3.2
OXS007570 (6)	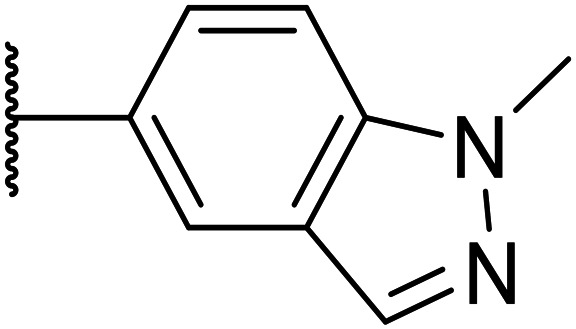	F	2.7	0.57	15	n.d.	7.43	3.3	5.3
12	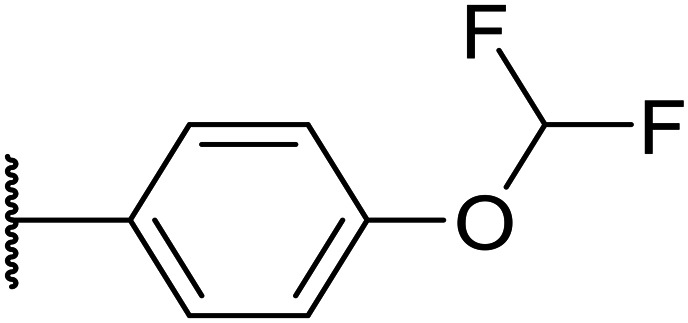	CN	370	0.02	68	n.d.	0.013	3.8	2.6
13	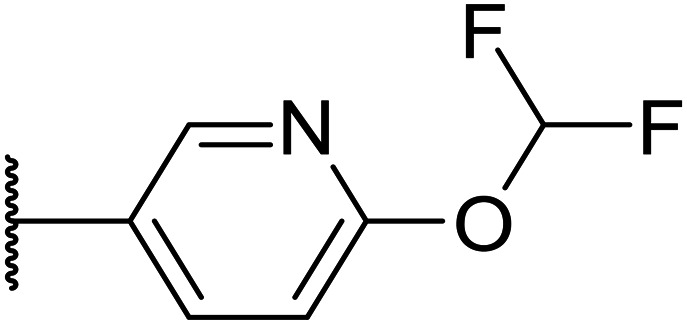	F	370	0.09	161	n.d.		3.4	3.0
14	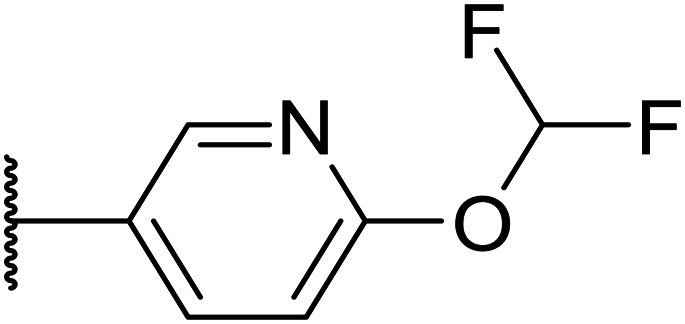	CN	2000	0.02	177	n.d.		3.2	2.5
15	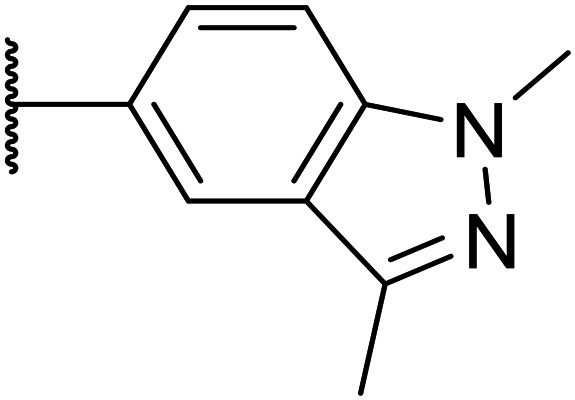	F	24	0.68	7	n.d.		3.7	3.9
16	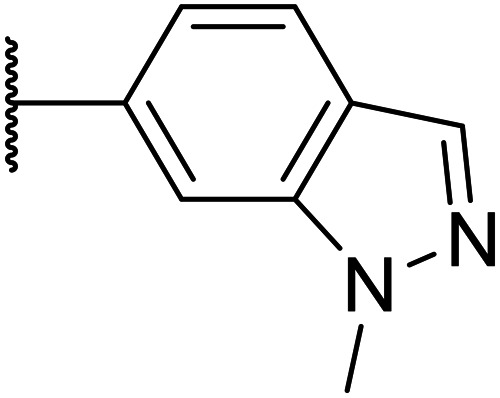	F	2500	n.d.	n.d.	n.d.		3.3	2.3
17	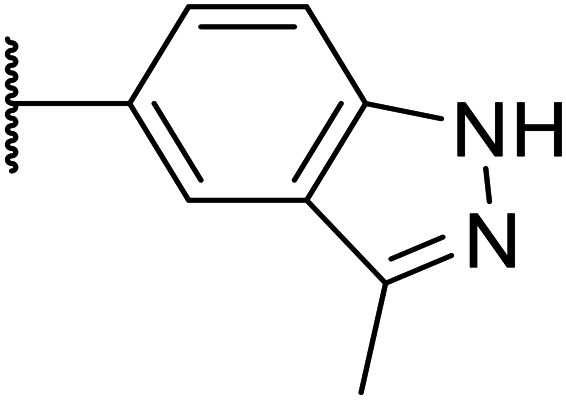	F	169	n.d.	n.d.	n.d.		3.5	3.3
18	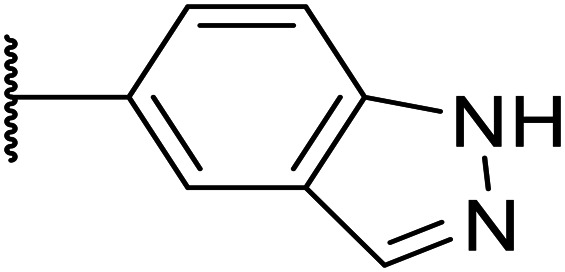	F	14	40	1	96%	n.d.	3.1	4.8
19	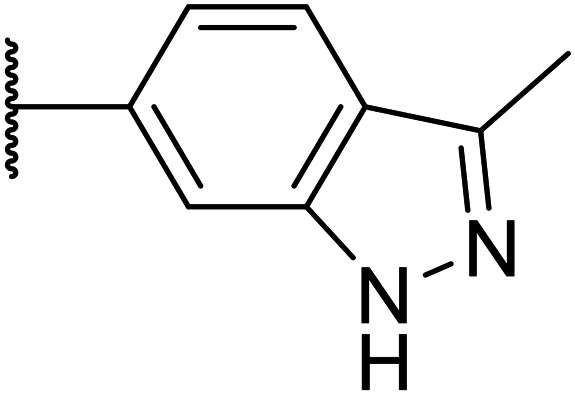	F	0.5	0.46	51		3.41	3.5	5.8
OXS008255 (20)	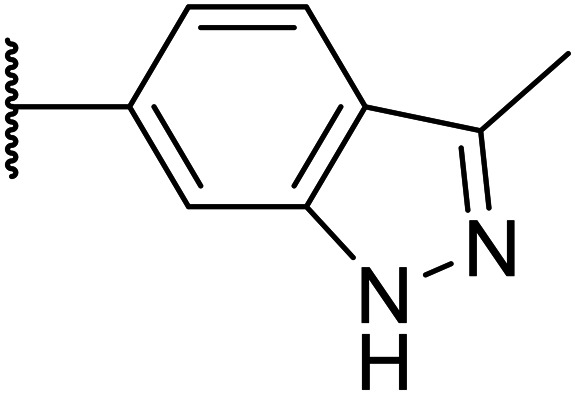	CN	0.4	0.26	27	64%	n.d.	3.3	6.1
21	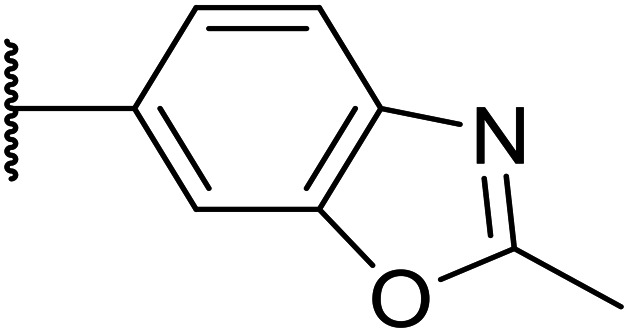	F	2.7	n.d.	n.d.	68%	n.d.	4.2	4.4
22	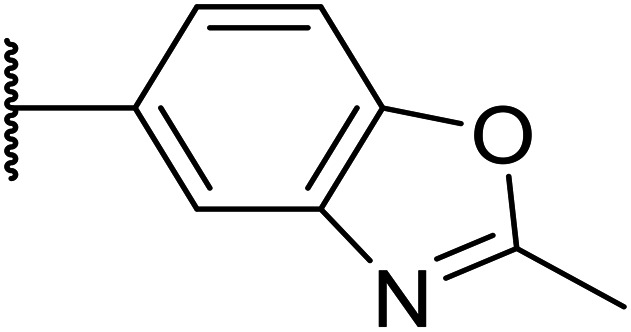	F	23	n.d.	n.d.	n.d.		4.2	3.4
23	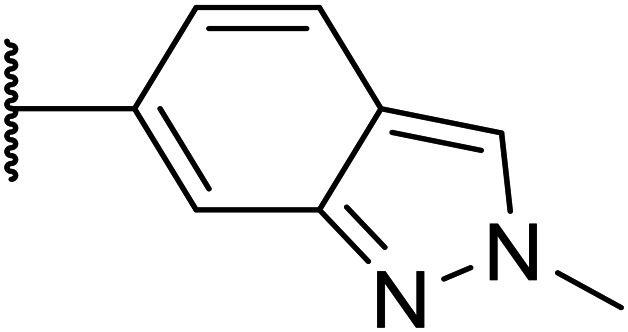	F	3.6	1	15	n.d.		3.3	5.1
24	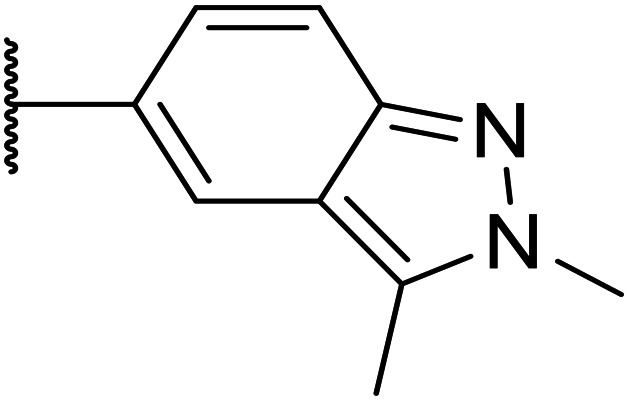	F	46	0.97	7	n.d.		3.7	3.6

a %CD11b response in HL-60 cells.

b Extraction ratio (ER) = Cl_int_/species flow rate (mice: 90 ml min^−1^ kg^−1^) in mouse S9 fraction (mS9), high (>0.7), intermediate (0.3–0.7) or low (<0.3).

c Semi-thermodynamic aqueous solubility.

d hERG inhibition, determined using a patch clamp assay.

e Percentage inhibition of the hERG channel at 30 μM.

f Calculated octanol–water partition coefficient clog *P* determined using Datawarrior.

g Lipophilic efficiency LLE = −log EC_50_ – clog *P*.

h Not determined.

Next, a variety of 6,5 fused heterocycle ring systems at C-6 were synthesised and tested (Table 2) to examine further similar structures to the NMe-indazole with the objective of delivering higher solubility and metabolic stability, and lower hERG activity. Incorporation of an extra methyl onto the indazole ring (15) reduced the potency 10-fold and gave no improvement in metabolic stability. The change of regiochemistry in 16 and 17 led to a 1000- and 60-fold drop of potency respectively as compared to 6, and removal of the NMe to NH (18) led to another slight activity drop demonstrating the importance of this site of the compound as a major pharmacophore. Interestingly, analogue 19, which retains the methyl position but changes the regiochemistry of the indazole, gave a 5-fold increase in potency to an EC_50_ of 0.5 nM. The solubility and metabolic stability of this compound also slightly improved in comparison to OXS0007570 (6), however the hERG activity increased. The corresponding *p*-CN 20 retained the nanomolar potency while increasing the metabolic stability from ER of 0.46 to 0.25. The hERG was still in the micromolar potency range, but the therapeutic window was improved due to the high potency of these two examples, which additionally have the highest LLE of the analogues in this sub-series. Benzoxazole examples 21 and 22 demonstrated good potency but were found early on to be chemically unstable in acidic media and so were not progressed further. Indazole 23 had equivalent potency to OXS007570 (6). A 10-fold decrease in potency was observed for the double-methylated 24 compared to the monomethyl derivative.

### 2.2. Addition of nitrogen atoms to the attached aromatic rings

Taking inspiration from compound 8 bearing a pyridine, where an additional nitrogen atom led to improved metabolic stability and solubility, and reduced hERG binding with respects to phenyl 6, our next goal was to introduce additional nitrogen atoms into the indazole benzene ring at different positions to lower the electron density of the ring. It was hoped that the additional nitrogens would contribute to improve metabolic stability and hERG activity, and that in some positions, potency would be retained.

Thus, the relevant pyrazolopyridine analogues shown in Table 3 were synthesised and tested. When a nitrogen atom was incorporated into the 4-position of the indazole ring (25), potency was reduced and metabolic stability was not improved, however, the hERG liability was significantly reduced (39% binding at 30 μM) which could be ascribed to a change in the dipole vector of the molecule. Derivative 26 with the nitrogen at the 6-position showed good potency but no improvement in other properties tested. When the nitrogen was placed at the 7-position (27), the best overall properties were exhibited in this series. The potency was slightly lowered to 23 nM as compared to 6, but solubility was dramatically increased to >200 μM, metabolic stability decreased to an ER of 0.23, and hERG activity was significantly reduced to 37% binding at 10 μM.

**Table 3:** Introduction of nitrogen atoms into the indazole at N-1

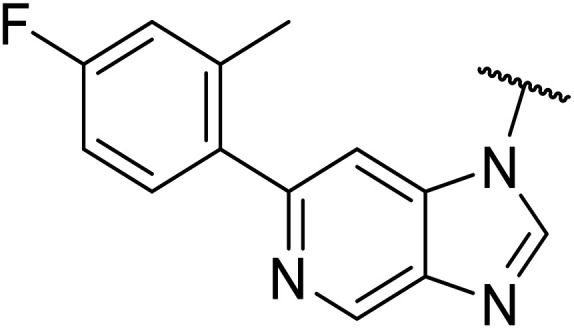								
Compound	R	EC_50_^a^ [nM]	ER in mS9^b^	Solubility^c^ [μM]	hERG^d^		clog *P*^f^	LLE^g^
					% inh^e^	IC_50_ [μM]		
25	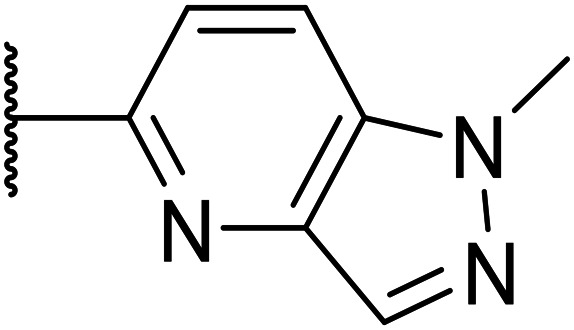	84	0.63	17	39%	n.d.^h^	3.9	3.2
26	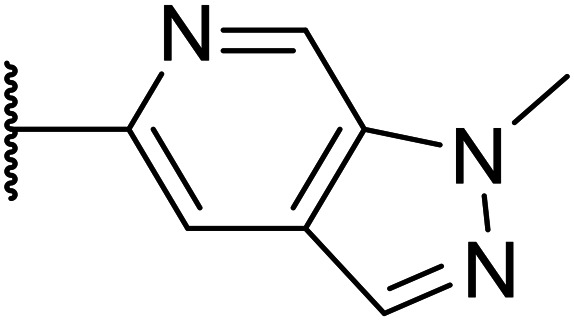	6.7	0.62	12	78%	n.d.	3.7	4.5
OXS008203 (27)	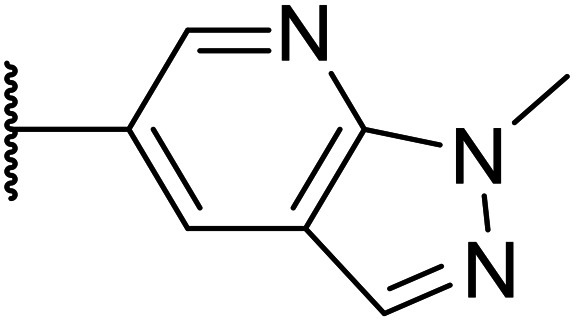	23	0.23	>200	n.d.	18	2.6	5.0

a %CD11b response in HL-60 cells.

b Extraction ratio (ER) = Cl_int_/species flow rate (mice: 90 ml min^−1^ kg^−1^) in mouse S9 fraction (mS9), high (>0.7), intermediate (0.3–0.7) or low (<0.3).

c Semi-thermodynamic aqueous solubility.

d HERG inhibition, determined using a patch clamp assay.

e Percentage inhibition of the hERG channel at 30 μM.

f Calculated octanol–water partition coefficient clog *P* determined using Datawarrior.

g Lipophilic efficiency LLE = −log EC_50_ − clog *P*.

h Not determined.

Following the positive results observed when adding nitrogen atoms into the aromatic rings, both in terms of physicochemical properties and hERG activity, we designed a final library of compounds that combined the incorporation of extra nitrogens at both C-6 and N-1 substituents (Table 4). Changing the regiochemistry of indazole 8 to 28 had a small effect on potency and hERG activities, but the metabolic stability was poor. Installation of the pyrazolopyridine at C-6 with the pyridine nitrogen at the 4- (29) and 6-positions (30) resulted in a loss of potency. However, when the *N* of the pyridine was located at the 7-position (31) the potency was 21 nM with reasonable solubility (86 μM), excellent metabolic stability (ER = 0.03) and only weak activity on the hERG channel (26% inhibition at 30 μM). Maintaining the pyridine nitrogen at the same position but changing the regioisomer at the pyrazole (32) also gave excellent metabolic stability and even lower hERG affinity, albeit CD11b activity and solubility were worse. The NH indazole of 33, which in example 19 and 20 had given picomolar potency compounds, in this example it gave a 3.5 nM potency with good solubility, metabolic stability, and hERG binding. Instead, the corresponding pyrazolopyridine 34 showed a loss of potency, having an EC_50_ of 285 nM. Benzisoxazole 35 had good activity, but as it came with a higher log *D* and lower LLE this was not progressed.

**Table 4:** Introduction of nitrogen atoms at both C-6 and N-1 substituents

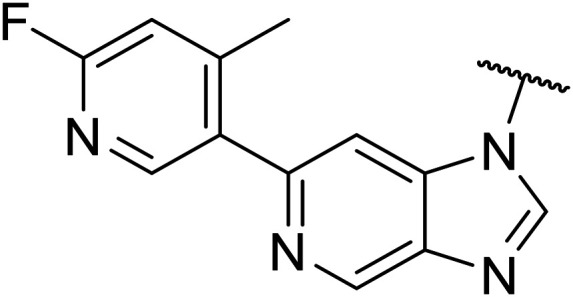							
Compound	R	EC_50_^a^ [nM]	ER in mS9^b^	Solubility^c^ [μM]	hERG% inh^d^	clog *P*^e^	LLE^f^
8	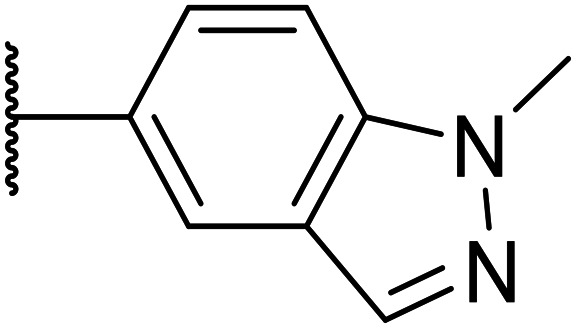	15	0.11	200	46%	2.6	5.2
28	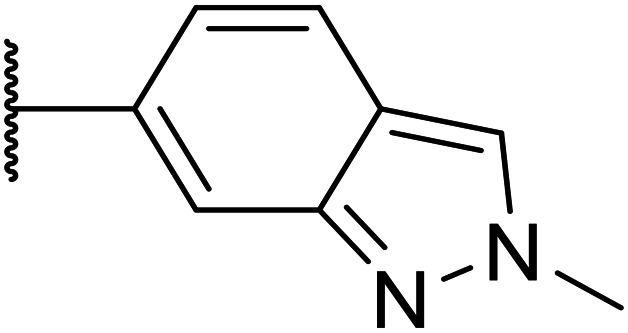	32	0.68	>200	42%	2.6	4.9
29	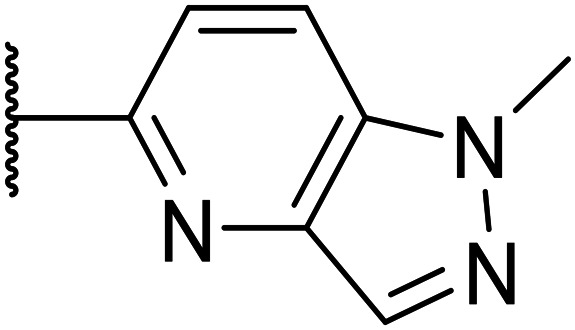	674	n.d.^g^	n.d.	n.d.	3.2	3.0
30	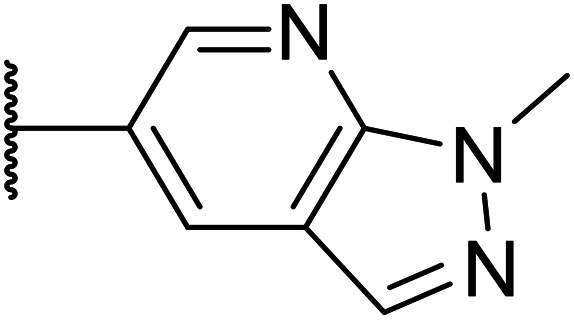	317	n.d.	n.d.	n.d.	1.9	4.6
31	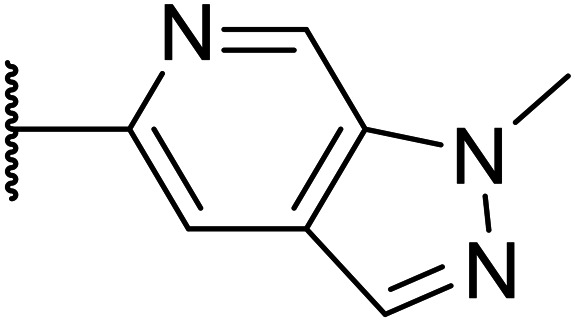	21	0.03	86	26%	3.1	4.6
32	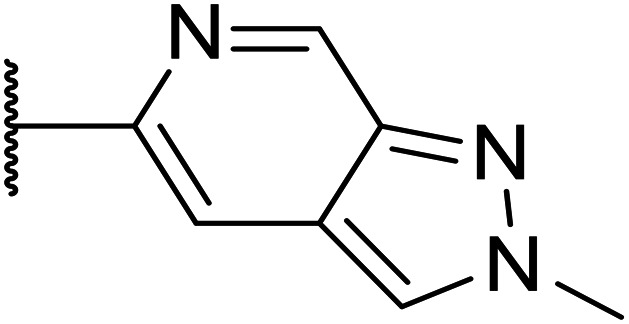	82	0.03	40	18%	3.1	4.0
33	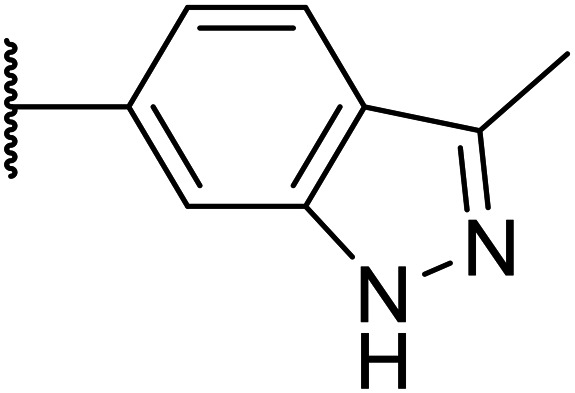	3.5	0.13	125	40%	2.9	5.6
34	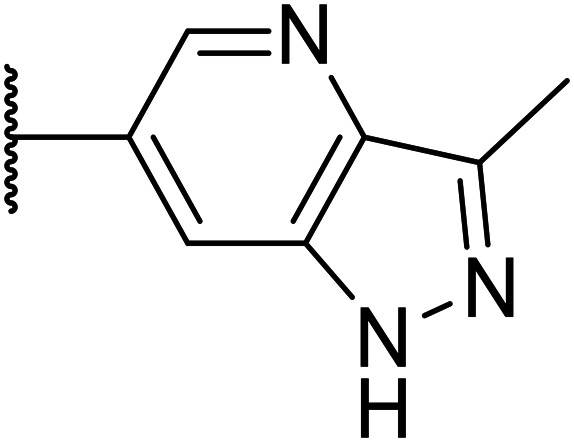	285	n.d.	n.d.	n.d.	2.0	4.5
35	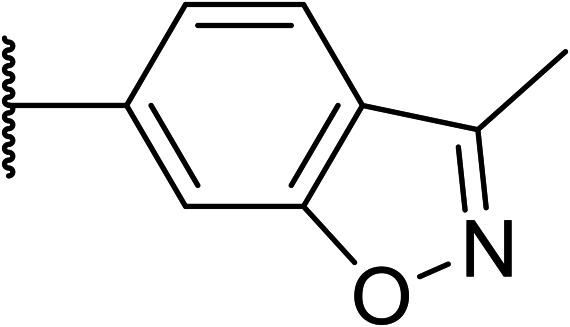	17	n.d.	n.d.	n.d.	3.4	4.4

a %CD11b response in HL-60 cells.

b Extraction ratio (ER) = Cl_int_/species flow rate (mice: 90 ml min^−1^ kg^−1^) in mouse S9 fraction (mS9), high (>0.7), intermediate (0.3–0.7) or low (<0.3)

c Semi-thermodynamic aqueous solubility.

d hERG inhibition, determined using a patch clamp assay, shown as percentage inhibition at 30 μM.

e Calculated octanol–water partition coefficient clog *P* determined using Datawarrior.

f Lipophilic efficiency LLE = −log EC_50_ – cLog *P*.

g Not determined.

### 2.3. Evaluation of pharmacokinetic profile

**Fig. 2:**
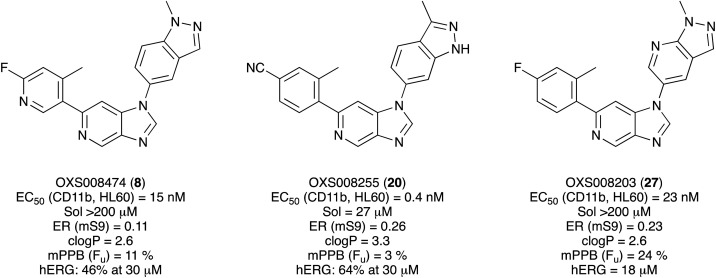
Overall profile of compounds selected for pharmacokinetic studies. Sol = solubility, ER = extraction ratio, mS9 = murine liver S0 fraction, mPPB = murine plasma protein binding, F_u_ = fraction unbound, hERG = human ether-a-go-go-related gene.

Given the positive *in vitro* profiles of some of the described analogues, the next step was to examine their pharmacokinetic properties. The compounds selected to be progressed into PK studies were OXS008255 (20), and OXS008203 (27) and OXS008474 (8) which demonstrated overall bioactivity, physicochemical, and *in vitro* ADME properties which were notably improved relative to OXS007417 (Fig. 2).

**Fig. 3:**
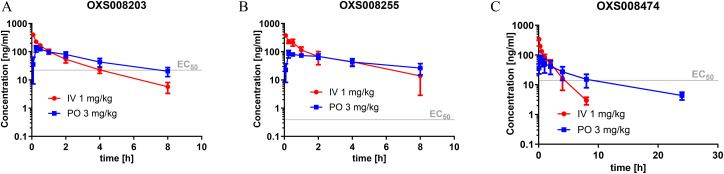
Pharmacokinetic profile of (A) OXS008203, (B) OXS008255, and (C) OXS008474. Observed total blood concentration profile in mice following single 3 mg kg^−1^ PO and 1 mg kg^−1^ IV dosing, *N* = 3, data plotted as mean ± SD.

The pharmacokinetic profile previously evaluated for OXS007417 in male CD-1 mice, dosed at 3 mg kg^−1^ PO and 1 mg kg^−1^ IV, had shown a moderate clearance (64 mL min^−1^ kg^−1^) and volume of distribution (4.6 L kg^−1^), leading to an elimination half-life of 1.5 h, with a bioavailability of 48%. At 3 mg kg^−1^, the *C*_max_ was 128 ng mL^−1^, which is 7-fold higher than the EC_50_*in vitro*, and compound concentration above the EC_50_ was maintained for 5 h. In comparison, OXS008255 with the same dosing regimen (Fig. 3B) also showed a moderate clearance (31 mL min^−1^ kg^−1^) and volume of distribution (5.0 L kg^−1^). The elimination half-life was higher at 6.2 h, and due to the picomolar EC_50_ the compound, concentration was maintained higher than the EC_50_ for over 24 h when extrapolating the curve.

OXS008203 and OXS008474 demonstrated improved clearances (41 and 52 mL min^−1^ kg^−1^) and elimination half-lives of 5.0 h and 7.5 h respectively with respects to OXS007417. Total compound concentrations were maintained at higher than the cellular EC_50_ for 8 h for OXS008203 and 10 h for OXS008474. These pharmacokinetic profiles were much improved in terms of half-life in comparison to OXS007417, each compound having coverage above the cell EC_50_ for at least 8 h at 3 mg kg^−1^ PO dosing (Table 5). In subsequent tolerability studies, OXS008203 was well tolerated up to 10 mg kg^−1^ QD for 8 days (Fig. S1†) and OXS008474 up to 3 mg kg^−1^ (Fig. S3†). OXS008255 was tolerated at 1 mg kg^−1^ QD but showed some atrophies at the thymus when treated at 3 mg kg^−1^ QD (Fig. S2†).

**Table 5:** Pharmacokinetic parameters of OXS008203, OXS008255, and OXS008474 determined in the single dose 3 mg kg^−1^ PO and 1 mg kg^−1^ IV studies

	OXS008203	OXS008255	OXS008474
PO *t*_1/2_ [h]^a^	5.04	6.17	7.48
PO AUC [ng h mL^−1^]^b^	510	428	391
PO *C*_max_ [ng mL^−1^]^c^	137	94.1	80.4
PO *F* [%]^d^	42.6	27.4	44.6
IV Cl_obs_ [mL min^−1^ kg^−1^]^e^	41.0	31.1	51.9
IV Vd_ss_ [L kg^−1^]^f^	4.78	4.96	4.56

a Elimination half-life.

b Area under the curve.

c Maximum concentration.

d Bioavailability.

e Clearance.

f Volume of distribution.

Moving forwards, it was hoped that a lower dosing regimen could be utilised effectively with these compounds compared to that used for OXS007417 to further improve the therapeutic window.

### 2.4. *In vivo* efficacy studies

**Fig. 4:**
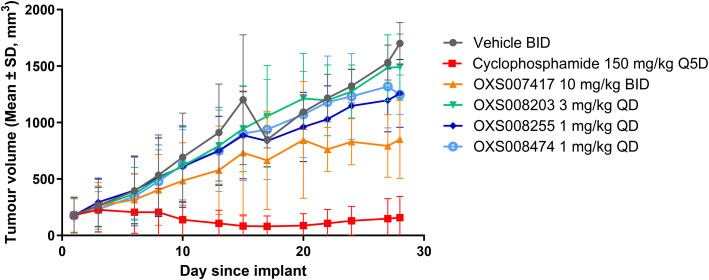
Evolution of tumour volume in the HL-60 subcutaneous xenograft model under treatment with selected compounds. Values shown are mean ± SD; *n* = 10 at day 0.

To evaluate the compounds in a disease relevant *in vivo* model, the efficacy of OXS008203, OXS008255, and OXS008747 was evaluated in a subcutaneous xenograft model with HL-60 cells implanted onto the flank of female NOD SCID mice (Fig. 4). Cyclophosphomide, a chemotherapy agent, and the initial lead compound OXS007417, were used as internal controls. Our previous study found OXS007417 to significantly delay the growth of HL-60 tumours with *T*/*C* (test *versus* control) of 55%, when compared to vehicle group (*p* < 0.0001) at 10 mg kg^−1^ using twice daily dosing. Due to the improved potency and PK profiles of the new generation compounds, lower dosing regimens were selected for this new study. A once daily dose of 1 mg kg^−1^ was selected for OXS008255 and OXS008474, whereas a once daily dose of 3 mg kg^−1^ was selected for OXS008203 to account for its lower elimination half-life. Based on the PK results, it was expected that these doses would maintain constant total compound blood levels above their cellular EC_50_.

As expected, the three compounds were well tolerated – bodyweights remained similar to, or above, 100% of pre-treatment level over the course of the 28 day dosing period, with no observations of adverse clinical signs or concerns around animal welfare.

OXS008203 at 3 mg kg^−1^ QD showed an insignificant delay in HL-60 tumour growth (*T*/*C* of 86% and *p* = 0.40), leading us to believe that higher or twice daily dosing would be needed for this compound to show a significant effect.

In contrast, even at 1 mg kg^−1^ QD, OXS008474 and OXS008255 showed a trend to reduced tumour growth compared to vehicle treated controls. At day 28, OXS008474 had *T*/*C* of 70.3% and OXS008255 of 71.0%, with *p* values of 0.018 and 0.031 respectively. This key result demonstrated that the new generation compounds with improved *in vitro* properties, therapeutic window, PK profiles, and reduced hERG liabilities were still active in a disease relevant model.

We also confirmed that representative compound OXS008474 inhibits polymerisation of tubulin (Fig. S4†), supporting that these optimised molecules maintain the same mechanism of action as the original OXS007417.^13^

## 3. Discussion

In this study, using the early lead compound OXS007417 and its derivative OXS007570 as starting points, we undertook multi-parameter optimisation to improve the potency and DMPK properties whilst retaining the *in vivo* efficacy of this imidazo[4,5-*c*]pyridine compound series (Fig. S5†). The affinity for the hERG channel was a key parameter to mitigate the study and alleviating this affinity was a high priority to move the project forward. The N-1 indazole in OXS007570 was seen to increase potency but had been previously discounted due to a drop of metabolic stability and solubility. Revisiting this group led the SAR in a new direction.

Firstly, with the N-1 indazole in place, a small survey of different groups at the C-6 position showed that the compounds' metabolic stability could be improved dramatically and the indazole was not a metabolic liability as first thought (Table 1). A *p*-CN motif improved extraction ratio and lowered clog *P*. The addition of a nitrogen atom onto pyridine OXS008474 not only improved metabolic stability but also reduced hERG activity and hugely improved solubility. In these systems, an altered electron density at the C-6 aromatic seems to lead to a reduction of enzymatic oxidation and increase in metabolic stability, with concomitant effect on hERG binding.

Next, to explore the N-1 indazole functionality in-depth, we synthesised and tested a variety of 6,5 heterobicycles (Table 2). The indazole regiochemistry was found to be of paramount importance and specifically the vector of the methyl group. As an example, regioisomers OXS007570 and 16 had a 60-fold difference in potency with a change of position of the NMe group from the 5 to the 7 positions. The methyl group can also be carbon linked – analogue 19, which incorporates a CMe group in the 5 position of the indazole and a NH in the 7 position, showed a 5-fold increase of potency to an EC_50_ of 0.5 nM. Combining this with a *p*-CN identified OXS008255, which had an improved metabolic stability and reduced hERG liability whilst maintaining exquisite potency, having a cellular EC_50_ of 0.4 nM. This did come with a decrease of solubility, perhaps due to the introduction of further sp2 character. The impressive increase in potency could be caused by a new interaction between the NH of 19 and OXS008255 and the binding target, this motif not being present in compound 6. Instead, indazole regioisomer with the CMe in the 7 position (17) had a lower potency, the cell EC_50_ being 169 nM. Analogues with the methyl group in the 6-position (21–23) also had good activity, providing further options for SAR exploration.

Given the positive results in certain examples where the phenyl ring is replaced by a pyridine ring (*e.g.*, 6*vs.*8), we followed a similar strategy to add additional nitrogen atoms onto the indazole at N-1 (Table 3). This led us to identify OXS008203, which had improved metabolic stability, excellent solubility, and a significant reduction of hERG binding compared to earlier compounds. The introduction of the pyridine motif could reduce the electron density of the aromatic ring and the lipophilicity of the compound, imparting the desired property improvements. Lastly, adding nitrogen atoms on both rings (Table 4) provided the lowest extraction ratios in this compound series. Compounds 31 and 32 both had excellent metabolic profiles, with ERs of 0.03. Depending on the pyrazolopyridine regioisomer at N-1, the potency could be retained (*e.g.*31, EC_50_ = 21 nM), but in general we observed a reduction.

With the new generation of compounds in hand, PK studies were undertaken with three selected compounds to determine how their *in vivo* profiles compared to the previous lead compound OXS007417. The three compounds chosen were the highly active compound OXS008255, a C-6 pyridine example OXS008474 and pyrazolopyridine OXS008203. The results for all three compounds showed improved elimination half-lives in comparison to OXS007417. Pyridine compound OXS008474 performed best in each scenario with an S9 extraction ration of 0.11 and an elimination half-life of 7.5 h.

The improved potencies and PK profiles gave confidence that progression to evaluation in a translationally relevant disease model was warranted, and that a low dose of compound could be used. The dosing regimen used previously for OXS007417 was 10 mg kg^−1^ BID, for the new study we chose to use 1 mg kg^−1^ QD for OXS008255 and OXS008474, and 3 mg kg^−1^ QD for OXS008203. Although the former compounds showed a trend to reduce tumour volume, the *T*/*C* values were lower than OXS007417 and an alternative dosing regimen might be needed for future studies. When performing our modelling to select suitable doses for our *in vivo* studies we compared total plasma concentrations of the compounds with *in vitro* EC_50_ values. We did this as the cell culture media for our *in vitro* experiments contained 10% foetal bovine serum, and previous studies have shown for drugs with similar levels of protein binding to our compounds (76–97%, Fig. 2), comparable levels of free drug between such cell culture conditions and in plasma samples.^28^ However, total protein levels and also protein composition can vary between these conditions, and between species,^29^ and this discrepancy may have led to an underestimation of the dosage needed to achieve a more significant reduction in tumour size in our efficacy experiments. A more detailed analysis of free compound levels in our cell culture conditions and *in vivo* would be needed to address this issue.

Overall, in this work we aimed to remove the hERG liability of our previous lead compound OXS007417. Our lead optimisation campaign was also directed to improve the metabolic stability and solubility properties. Initially, our general approach was to lower the log *P*, and initially as we moved from OXS007417 (hERG = 110 nM, clog *P* 4.1) to OXS007570 (hERG = 7.4 μM, log *P* 3.3) this strategy seemed to follow this trend. However, it quickly became apparent with more examples that driving optimisation based solely on the clog *P* was not sufficient and there was not a clear correlation between hERG binding and clog *P*. In this study, the data suggests that localised changes in electron density in certain key sites of the compounds are more significant than a general decrease in lipophilicity. The most successful examples had nitrogen atoms replacing CH groups at either the N-1 or C-6 aromatic rings (*e.g.*, CH analogue OXS007570, IC_50_ (hERG) = 7.4 μM *vs.* N analogue OXS008474, IC_50_ (hERG) ≥ 30 μM). The change to a pyridine ring from the benzene ring appears to have a big effect on hERG channel binding in this system; this may be due to the lowered electron density on the ring system no longer facilitating favourable π interactions with the hERG channel.

We expect our learnings to be of use for the medicinal chemistry community aiming to improve physicochemical properties of compound series, and to contribute to the strategies that can be used to alleviate a hERG liability.

## 4. Conclusion

In summary, the early lead compound OXS007417, an early lead molecule acting as a differentiation therapy for the potential treatment of AML, has been optimised to improve the potency, physicochemical, and pharmacokinetic properties and to alleviate an off-target hERG channel interaction. A new generation of compounds bearing an indazole pharmacophore were identified that had improved potency, solubility, and metabolic stability in comparison to OXS007417. In addition to this, site selective nitrogen addition was utilised to remove the hERG liability while maintaining high solubility and metabolic stability, and to create a wide therapeutic window for the compounds. The next generation compounds OXS008255 and OXS008474 had improved pharmacokinetic properties and demonstrated efficacy by delaying tumour growth in *in vivo* studies using a subcutaneous model with HL-60 cells.

## Data availability

The data supporting this article have been included as part of the ESI.†

## Ethical statement

All animal procedures were performed in accordance with the Guidelines for Care and Use of Laboratory Animals, and were approved by the Animal Welfare and Ethical Review Body of Axis Bioservices.

## Author contributions

T. J. C., L. J.-C., Da. Z., S. R. G. G., M. J.-S., K. S. H., T. R. J., T. A. M., G. M. W., and A. J. R. conceived the experimental design. T. J. C., L. J.-C., Da. Z., S. R. G. G., M. J.-S., K. S. H., T. R. J., and Do. Z. carried out experiments and analysed the data. T. J. C. and L. J.-C. wrote the manuscript. T. J. C., L. J.-C., Da. Z., S. R. G. G., M. J.-S., K. S. H., T. R. J., S. G. D., T. A. M., G. M. W., and A. J. R. interpreted the data. All authors contributed to reviewing and editing the manuscript. P. V., A. J. R. and T. A. M. provided supervision and funding.

## Conflicts of interest

The authors declare the following financial interests/personal relationships which may be considered as potential competing interests: A. J. R., T. A. M., S. G. D. and P. V. are founders and minor shareholders of OxStem Oncology, a subsidiary company of OxStem Ltd. G. M. W. is a minor shareholder of OxStem Ltd. At the time the work was conducted, A. J. R. and G. M. W. were paid consultant for OxStem Ltd. T. A. M. is currently a paid consultant for and minor shareholder of Dark Blue Therapeutics Ltd.

## Supplementary Material

MD-OLF-D4MD00275J-s001
