# Supplementary material for: Lead optimisation of OXS007417: in vivo PK profile and hERG liability modulation to optimise a small molecule differentiation agent for the potential treatment of acute myeloid leukaemia
Source: RSC Med Chem. 2024 Jul 22;15(10):3495–506. doi: 10.1039/d4md00275j (PMC11361297; doi:10.1039/d4md00275j)
Supplement: MD-015-D4MD00275J-s001 [file MD-015-D4MD00275J-s001.pdf]

Supplementary Information (SI) for RSC Medicinal Chemistry.
This journal is © The Royal Society of Chemistry 2024

SUPPORTING INFORMATION

Lead optimisation of OXS007417: *in vivo* PK profile and hERG liability modulation to optimise a small molecule differentiation agent for the potential treatment of Acute Myeloid Leukaemia

Thomas J. Cogswell,[1,4] Laia Josa-Culleré,[1,4] David Zimmer,[1] Sébastien R. G. Galan,[1] Morgan Jay-Smith,[1] Kate S. Harris,[1] Thomas R. Jackson,[2] Douzi Zhang,[2] Stephen G. Davies,[1] Paresh Vyas,[2] Thomas A. Milne,[2] Graham M. Wynne,[1] Angela J. Russell[1,3]

[1] Department of Chemistry, Chemistry Research Laboratory, University of Oxford, Mansfield Road, Oxford OX1 3TA, UK.

[2] MRC Molecular Haematology Unit, MRC Weatherall Institute of Molecular Medicine, Radcliffe Department of Medicine, University of Oxford, Oxford OX3 9DS, UK.

[3] Department of Pharmacology, University of Oxford, Mansfield Road, Oxford OX1 3QT, UK.

[4] These authors contributed equally to this work.

Table of contents

Biological testing experimental procedures..3

Chemistry synthesis experimental procedures...5

Supplementary Figures 43

NMR spectra of final compounds..46

References...73

Biological testing experimental procedures

Cell culture

AML cell line HL-60 was purchased from the American Type Culture Collection (ATCC). The cells were maintained in RPMI supplemented with 10% fetal bovine serum and 1% L-glutamine.

Flow cytometry

PE mouse-anti-human-CD11b/Mac-1 (ICRF44, Cat 555388) was purchased from BD Bioscience. Cells were suspended in 100 µL growth media (RPMI + 10% FBS + 1% L-glutamine) at a density of $2x10^4$ cells/well of a V-bottom 96 well plate (Corning) and grown for 96 hours in the presence of compound at the required concentration. After 96 hours, the plate was centrifuged, media was removed, and cells were resuspended in 50 µL of blocking buffer (IMDM, no phenol red + 10% FBS) containing 2.5 µL of CD11b antibody and incubated on ice in the dark for 20 min. Cells were washed twice in 200 µL of staining buffer (IMDM, no phenol red + 2% FBS) before being resuspended in 200 µL of staining buffer containing 1 µg/ml of DAPI to help identify dead cells. Flow cytometry data was collected on an Attune NxT and analysed using the Attune NxT software (Life Technologies).

ADME properties

Determination of solubility, metabolic stability, and PPB was performed by Cyprotex Ltd. (www.cyprotex.com) using their standard assays.

PK studies

Male CD-1 mice were used for the PK studies, 3 for each treatment in a cassette study group. The compounds were dosed either PO (3 mg/kg) or IV (1 mg/kg), and blood concentration was measured by LC−MS/MS over 24 h.

Tolerance studies

Male CD-1 mice were dosed PO with 1, 3, and 10 mg/kg of the compounds once a day for 8 consecutive days, 2 for each treatment. The animals were weighed every day and observed for any adverse effects, including their behaviour and activity, body condition, respiration, appearance, dietary status, excretion, and other gross lesions. At the end of the study, the animals were euthanised and gross necropsy was performed to analyse abdominal (digestive and genitourinary system), thoracic, heart, lung, thymus, cranial cavity and external surface of the brain.

***In vivo* efficacy study**

Female NOD SCID mice aged 5-7 weeks of age were used for the HL-60 subcutaneous model. Cells ($5x10^6$, 1:1 in Matrigel) were implanted subcutaneously under the flank of each mouse. When tumours reached approximately 150 mm^3, mice were grouped randomly into treatment groups based on their bodyweight to ensure equal distribution. Mice were treated by indicated treatment regimes. Drug solutions were formulated immediately prior to dosing.

Group	n	Treatment	Dose Level	Dosing Route	Dosing Regimen
1	10	Vehicle	--	PO	BID
2	10	Cyclophosphamide	150 mg/kg	IP	Q5D
3	10	OXS007417	10 mg/kg	PO	BID
4	10	OXS008203	3 mg/kg	PO	QD
5	10	OXS008255	1 mg/kg	PO	QD
6	10	OXS008474	1 mg/kg	PO	QD

Cyclophosphamide was formulated in sterile saline. Dosing volume was 10 ml/kg for IP dosing.

All OXS compounds was formulated in DMSO (final concentration of 5%) and PBS + 0.1% Tween 20. Dosing volume was 10 ml/kg for PO dosing.

The length and width of the tumours were measured 3 times per week using digital callipers. The length and width of the tumour will be measured, and volume calculated using the following formula: volume = (length x width2)/2. The study was terminated at the end of the 28-day treatment period. Tumours were removed from each animal and the wet tumour weight was recorded.

The bodyweight of all mice on the study were measured and recorded 3 times per week; no significant reduction was considered it animal lost less than 10% bodyweight. Additionally, mice were observed daily for any signs of distress or changes to general condition e.g. starred fur, lack of movement, difficulty breathing, and changes around the tumour area (e.g. inflammation or ulceration). All protocols used in this study were approved by the Axis Bioservices Animal Welfare and Ethical Review Committee, and all procedures are carried out under the guidelines of the Animal (Scientific Procedures) Act 1986.

One-way ANOVA with Dunnett's test was used for the statistical analysis.

Chemistry synthesis experimental procedures

General information

All reactions involving moisture sensitive reagents were carried out under a nitrogen or an argon atmosphere. Anhydrous solvents were dried by passing over an activated alumina column, under an inert atmosphere, using a solvent purification system. All other solvents and reagents were used as supplied (analytical or HPLC grade) without prior purification. *Flash* column chromatography was performed on Kieselgel 60 silica gel (230-400 mesh particle size) on a glass column or on a Biotage SP4 automated flash column chromatography platform. NMR spectra were recorded on Bruker Advance spectrometers at 400 or 500 MHz in the deuterated solvent stated at room temperature. The field was locked by external referencing to the relevant deuteron resonance. Chemical shifts (δ) are reported in parts per million (ppm) and coupling constants (*J*) are quoted in Hz. Data are reported as follows: chemical shift, multiplicity (s = singlet, d = doublet, t = triplet, q = quartet, sext = sextuplet, hept = heptet, and m = multiplet), coupling constant and integration. Low-resolution mass spectra (*m/z*) were recorded on an Agilent 1260 Infinity II with Diode Array and Single Quadrupole Detectors in solutions of MeOH. A selected peak is reported in Daltons and its intensity given as percentage of the base peak. High resolution mass spectra (HRMS) were run on a Bruker microTOF (ESI and APCI) or on a Waters GCT (EI). Experiments conducted in CROs used their standard equipment.

General synthetic routes

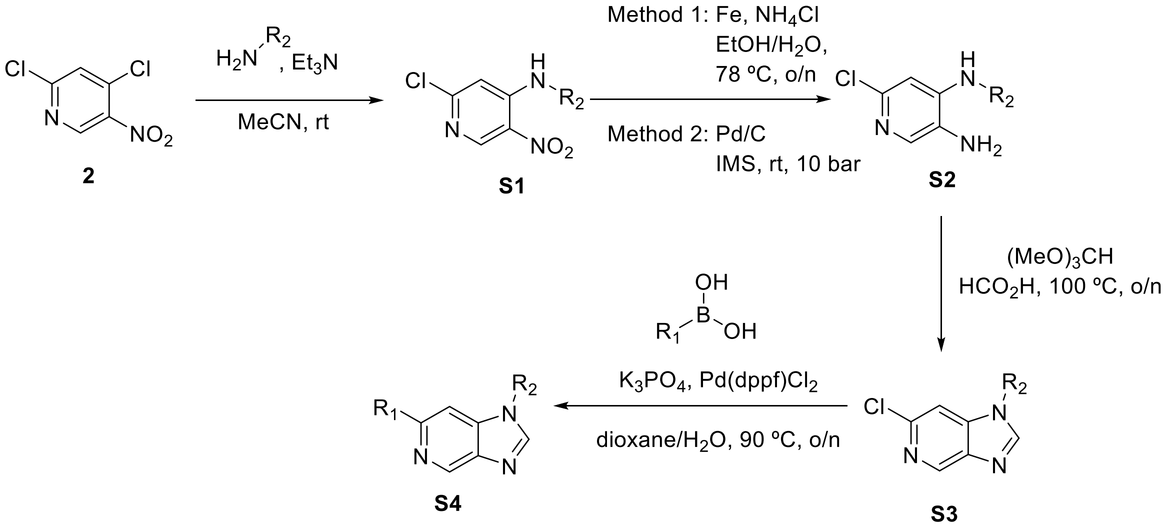

General procedure A: Nucleophilic aromatic substitution

To a solution of 2,4-dichloro-5-nitro-pyridine (1.0 equiv) in acetonitrile (*c* 0.5 M) were added the desired amine (1.0 equiv) and triethylamine (2.0 equiv). The mixture was stirred at room

temperature until full conversion and concentrated under reduced pressure. The resulting residue was redissolved in EtOAc, washed with 1xH_2O and 1xbrine, dried over anhydrous Na_2SO_4 and concentrated to give the amine **S1**, which was used in the following step without further purification.

General procedure B.1: Nitro reduction with Fe/NH_4Cl

To a suspension of compound **S1** (1.0 equiv) in ethanol/water (1:2, *c* 0.2) were added ammonium chloride (4 equiv) and iron powder (5 equiv). The reaction mixture was heated at reflux for 14 h, then cooled to room temperature, diluted with DCM and filtered through Celite. The filtrate was basified with saturated aqueous $NaHCO_3$, extracted with 3xDCM, washed with brine, dried over anhydrous Na_2SO_4 and concentrated to give amine **S2**, which was used in the following step without further purification.

General procedure B.2: Nitro reduction via hydrogenation

A solution of nitro **S1** (1.0 equiv) in IMS (*c* 0.01 M) was placed in an autoclave. Pd/C (10 wt.%, 0.05 equiv) wetted with water was added and the reaction was heated to 20-35 °C under 10-12 bar H_2 for 2 h. The mixture was then filtered through Celite® and evaporated to dryness. The crude residue was purified by *flash* column chromatography to give the amine **S2**.

General procedure C: Imidazole formation

Formic acid (1 equiv) was added to a solution of amine **S2** (1.0 equiv) in triethylorthoformate (*c* 0.3 M), and the mixture was stirred at 100 °C overnight. The reaction mixture was concentrated, and to the resulting residue was added saturated aqueous $NaHCO_3$, extracted with 3xEtOAc, dried over anhydrous Na_2SO_4 and concentrated to give imidazopyridine **S3**, which was used in the following step without further purification.

General procedure D: Suzuki reaction

To a solution of compound **S3** (1 equiv) in either 1,4-dioxane/water (4:1, *c* 0.2 M) or DME (*c* 0.5 M) under argon were added potassium phosphate or potassium carbonate (3 equiv), the desired boronic acid (1.2 equiv) and Pd(dppf)Cl_2 (0.1 equiv). The mixture was heated at 80-100 °C overnight, then diluted with EtOAc, filtered through Celite and concentrated. The crude product was purified by *flash* column chromatography to give the desired product **S4**.

Compounds **OXS007417**, **OXS007570**, **12**, and **13** were prepared using the reported methods.[1]

N-(2-Chloro-5-nitropyridin-4-yl)-1-methyl-1H-indazol-5-amine (3)

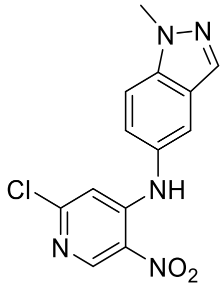

General procedure A; yield 90% (1.42 g, 4.69 mmol); orange solid. ^{1}H NMR (400 MHz, $CDCl_3$) δ 9.68 (s, 1H), 9.10 (s, 1H), 8.04 (d, J = 1.0 Hz, 1H), 7.72 – 7.46 (m, 2H), 7.28 (d, J = 2.0 Hz, 1H), 6.75 (s, 1H), 4.14 (s, 3H); m/z (ESI+) 304.0 ([M+H]+, 100%).

6-Chloro-N^4-(1-methyl-1H-indazol-5-yl)pyridine-3,4-diamine (4)

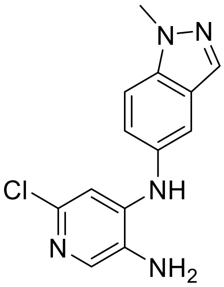

General procedure B.1 from **3** (1.40 g, 4.61 mmol); yield 78% (986 mg, 3.56 mmol); red-brown solid. ^{1}H NMR (400 MHz, $CDCl_3$) δ 7.96 (d, J = 1.0 Hz, 1H), 7.79 (s, 1H), 7.54 – 7.38 (m, 2H), 7.23 (dd, J = 8.9, 1.9 Hz, 1H), 6.70 (s, 1H), 6.07 (s, 1H), 4.11 (s, 3H); m/z (ESI+) 274.1 ([M+H]+, 100%).

6-Chloro-1-(1-methyl-1H-indazol-5-yl)-1H-imidazo[4,5-*c*]pyridine (5)

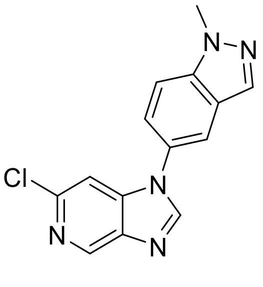

General procedure C from **4** (980 mg, 3.56 mmol); yield 84% (845 mg, 2.98 mmol); brown solid. ^{1}H NMR (400 MHz, $CDCl_3$) δ 8.96 (d, J = 1.0 Hz, 1H), 8.17 (s, 1H), 8.12 (d, J = 1.0 Hz, 1H), 7.82

(dd, J = 2.0, 0.7 Hz, 1H), 7.63 (dt, J = 8.8, 0.9 Hz, 1H), 7.48 – 7.38 (m, 2H), 4.19 (s, 3H); m/z (ESI+) 284.0 ([M+H]+, 100%).

3-Methyl-4-(1-(1-methyl-1*H*-indazol-5-yl)-1*H*-imidazo[4,5-*c*]pyridin-6-yl)benzonitrile (7)

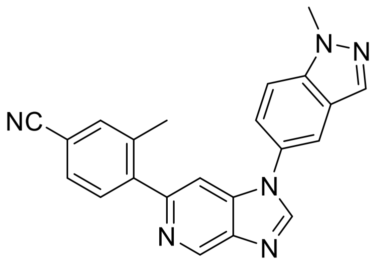

General procedure D from **5** (88 mg, 0.31 mmol) and 3-methyl-4-(4,4,5,5-tetramethyl-1,3,2-dioxaborolan-2-yl)benzonitrile (91 mg, 0.37 mmol); yield 48% (54 mg, 0.15 mmol); brown solid. ^{1}H NMR (500 MHz, $CDCl_3$) δ 9.29 (d, *J* = 0.9 Hz, 1H), 8.26 (s, 1H), 8.11 (d, *J* = 0.7 Hz, 1H), 7.86 (d, *J* = 1.8 Hz, 1H), 7.62 (d, *J* = 8.8 Hz, 1H), 7.58 (s, 1H), 7.56 – 7.52 (m, 1H), 7.52 – 7.47 (m, 3H), 4.18 (s, 3H), 2.40 (s, 3H); ^{13}C NMR (126 MHz, $CDCl_3$) δ 152.2, 145.5, 145.0, 143.1, 140.3, 139.9, 139.4, 137.9, 134.4, 133.4, 130.8, 129.7, 128.3, 124.4, 123.1, 119.1, 117.3, 111.8, 111.0, 106.0, 36.1, 20.5; *m/z* (ESI$^+$) 364.1 ([M+H]$^+$, 100%); HRMS (ESI$^+$) $C_{22}H_{17}N_6$ ([M+H]$^+$) requires 365.1510; found 365.1509.

6-(6-Fluoro-4-methylpyridin-3-yl)-1-(1-methyl-1*Hz*-indazol-5-yl)-1*H*-imidazo[4,5-*c*]pyridine (OXS008474, 8)

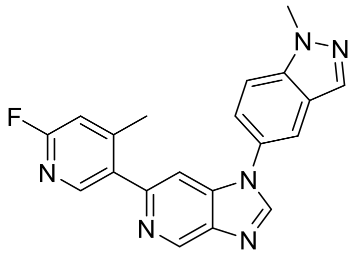

General procedure D from **5** (50 mg, 0.18 mmol); yield 13% (8 mg, 0.02 mmol); white solid. ^{1}H NMR (500 MHz, $CDCl_3$) δ 9.28 (s, 1H), 8.25 (s, 1H), 8.18 (s, 1H), 8.11 (s, 1H), 7.86 (d, *J* = 1.9 Hz, 1H), 7.63 (d, *J* = 8.6 Hz, 1H), 7.51 (dd, *J* = 8.8, 2.0 Hz, 1H), 7.49 (d, *J* = 1.0 Hz, 1H), 6.91 – 6.82 (m, 1H), 4.18 (s, 3H), 2.44 (s, 3H); ^{13}C NMR (125 MHz, $CDCl_3$) δ 163.5 (d, *J* = 239 Hz), 152.1 (d, *J* = 8 Hz), 149.9, 147.8 (d, *J* = 16 Hz), 144.9, 143.2, 140.2, 140.0, 139.4, 135.3 (d, *J* = 4 Hz), 133.4, 128.2, 124.4, 123.1, 117.3, 111.1, 110.6 (d, *J* = 37 Hz), 106.2, 36.1, 20.5 (d, *J* = 3 Hz); *m/z* (ESI$^+$) 359.1 ([M+H]$^+$, 80%); HRMS (ESI$^+$) $C_{20}H_{16}N_6F^+$ ([M+H]$^+$) requires 359.14150; found 359.14162.

4,4,5,5-Tetramethyl-2-(2-methylcyclohex-1-en-1-yl)-1,3,2-dioxaborolane (S5)

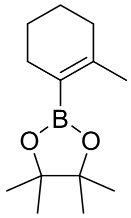

To a mixture of methyl cyclohexanone (500 mg, 4.46 mmol) and sodium carbonate (963 mg, 8.92 mmol) in CH_2Cl_2 (40 mL) at 0 °C was added trifluoromethanesulfonic anhydride (1.2 mL, 7.1 mmol) and the mixture stirred at room temperature overnight. The mixture was washed with water then brine, dried and concentrated. The crude product was purified by *flash* column chromatography (100% pentane) to give a colourless oil (1.0 g) that was used immediately. To a solution of the resultant oil in 1,4-dioxane (40 mL) under argon were added B_2Pin_2 (1.25 g, 4.91 mmol), potassium acetate (804 mg, 8.19 mmol) and Pd(dppf)Cl_2 (150 mg, 0.21 mmol). The mixture was heated at 80 °C overnight, then diluted with pentane, filtered through Celite, washed with brine, dried and concentrated. The crude product was purified by *flash* column chromatography (100% pentane) to give **S5** (339 mg, 1.52 mmol, 37%) as a colourless oil. ^{1}H NMR (500 MHz, $CDCl_3$) δ 2.12 – 2.05 (m, 2H), 2.02 – 1.95 (m, 2H), 1.92 – 1.87 (m, 3H), 1.62 – 1.55 (m, 2H), 1.55 – 1.47 (m, 2H), 1.26 (s, 12H).

1-(1-Methyl-1*H*-indazol-5-yl)-6-(2-methylcyclohex-1-en-1-yl)-1*H*-imidazo[4,5-*c*]pyridine (9)

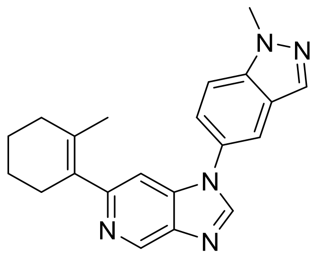

To a solution of **5** (40 mg, 0.14 mmol) in 1,4-dioxane/water (1:1, 820 µL) under argon were added potassium phosphate (90 mg, 0.42 mmol), borolane **S5** (38 mg, 0.17 mmol), and Pd(PPh_3)$_4$ (16 mg, 0.010 mmol). The mixture was heated at 90 °C overnight, then diluted with EtOAc, filtered through Celite, dried over anhydrous Na_2SO_4, and concentrated *in vacuo*. The crude product was purified by flash column chromatography to give **9** (36 mg, 0.10 mmol, 74%) as a brown solid. ^{1}H NMR (400 MHz, $CDCl_3$) δ 9.15 (d, J = 1.1 Hz, 1H), 8.13 (s, 1H), 8.09 (d, J = 1.0 Hz, 1H), 7.81 (dd, J = 2.0, 0.8 Hz, 1H), 7.60 (dt, J = 8.8, 0.9 Hz, 1H), 7.47 (dd, J = 8.8, 2.0 Hz, 1H), 7.23 (d, J = 1.1 Hz, 1H), 4.15 (s, 3H), 2.43 – 2.32 (m, 2H), 2.11 – 2.03 (m, 2H), 1.76 – 1.64 (m, 4H), 1.58 (s, 3H); ^{13}C NMR (100 MHz, $CDCl_3$) δ 156.1, 144.1, 142.7, 139.7, 139.3, 133.3, 132.7, 131.8, 128.6, 124.4, 123.2, 117.0, 110.8, 104.9, 36.0, 31.9, 30.8, 23.4, 23.0, 20.8; *m/z* (ESI$^+$) 344.1 ([M+H]$^+$, 100%); HRMS (ESI$^+$) $C_{21}H_{22}N_5^+$ ([M+H]$^+$) requires 344.18697; found 344.18716.

1-(1-Methyl-1*H*-indazol-5-yl)-6-(2-methylpiperidin-1-yl)-1*H*-imidazo[4,5-*c*]pyridine (10)

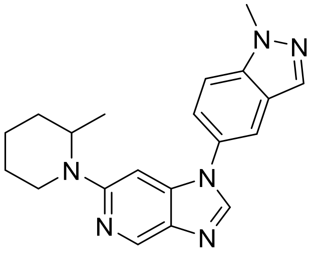

In a sealed vial, **5** (80 mg, 0.28 mmol), 2-methylpiperidine (99 µL, 0.85 mmol), sodium *tert*-butoxide (54 mg, 0.56 mmol), $Pd_2(dba)_3$ (26 mg, 0.030 mmol), and JohnPhos (17 mg, 0.060 mmol) in degassed toluene (950 µL) were purged with nitrogen for 10 min, the vial was sealed and heated to 110 °C for 3 h. The mixture was diluted with $CHCl_3$, filtered through Celite, and concentrated *in vacuo*. The crude residue was purified by flash column chromatography (EtOAc) and recrystallised from EtOAc to give the product (11 mg, 0.030 mmol, 11%) as a yellow solid. ^{1}H NMR (400 MHz, $CDCl_3$) δ 8.80 (d, *J* = 1.0 Hz, 1H), 8.10 (d, *J* = 1.0 Hz, 1H), 7.90 (s, 1H), 7.80 (dd, *J* = 1.9, 0.8 Hz, 1H), 7.59 (dt, *J* = 8.8, 0.9 Hz, 1H), 7.47 (dd, *J* = 8.8, 2.0 Hz, 1H), 6.49 (d, *J* = 1.1 Hz, 1H), 4.70 (p, *J* = 6.6 Hz, 1H), 4.17 (s, 3H), 3.90 – 3.79 (m, 1H), 2.92 (td, *J* = 12.3, 3.2 Hz, 1H), 1.90 – 1.59 (m, 6H), 1.07 (d, *J* = 6.7 Hz, 3H); ^{13}C NMR (126 MHz, $CDCl_3$) δ 157.1, 142.7, 142.4, 140.8, 139.1, 134.7, 133.2, 129.0, 124.3, 123.4, 117.0, 110.6, 86.4, 48.1, 41.0, 35.9, 30.7, 25.8, 19.0, 13.3; *m/z* (ESI^+) 347.1 ($[M+H]^+$, 100%); HRMS (ESI^+) $C_{20}H_{23}N_6^+$ ($[M+H]^+$) requires 347.1979; found 347.1978.

1-(1-(1-Methyl-1*H*-indazol-5-yl)-1*H*-imidazo[4,5-*c*]pyridin-6-yl)piperidin-2-one (11)

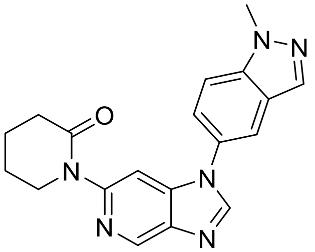

In a sealed vial, **5** (50 mg, 0.18 mmol), 2-piperidone (26 mg, 0.26 mmol), copper iodide (2 mg, 0.01 mmol), *trans*-*N*,*N*-dimethyl-1,2-cyclohexanediamine (1 µL, 0.01 mmol), and potassium carbonate (49 mg, 0.35 mmol) in dry 1,4-dioxane (180 µL) were purged with nitrogen for 10 min, the vial was sealed and heated to 150 °C overnight. The mixture was cooled down to room temperature, diluted with DCM, washed with saturated aqueous ammonia, the layers separated, and the aqueous layer extracted with 3xDCM. The combined organic layers were dried and evaporated under reduced pressure. The residue was purified by flash column chromatography (DCM to 95/5/0.5 DCM/MeOH/NH_4OH) to afford the product (38 mg, 0.11 mmol, 62%) as a yellow solid. ^{1}H NMR (400 MHz, $CDCl_3$) δ 9.00 (d, *J* = 1.0 Hz, 1H), 8.17 (s, 1H), 8.08 (d, *J* = 1.0

Hz, 1H), 7.82 (dd, J = 2.0, 0.8 Hz, 1H), 7.78 (d, J = 1.0 Hz, 1H), 7.57 (dt, J = 8.9, 0.9 Hz, 1H), 7.49 (dd, J = 8.8, 2.0 Hz, 1H), 4.15 (s, 3H), 3.97 (t, J = 5.8 Hz, 2H), 2.56 (t, J = 6.6 Hz, 2H), 2.01 – 1.88 (m, 4H); ^{13}C NMR (101 MHz, $CDCl_3$) δ 170.7, 149.1, 145.1, 141.0, 140.4, 139.5, 139.2, 133.2, 128.3, 124.2, 123.1, 117.0, 110.8, 102.9, 48.9, 35.9, 33.3, 23.3, 21.1; *m/z* (ESI$^+$) 347.1 ([M+H]$^+$, 100%); HRMS (ESI$^+$) $C_{19}H_{19}N_6O^+$ ([M+H]$^+$) requires 347.16149; found 347.16156.

_N_-(2-Chloro-5-nitropyridin-4-yl)-6-(difluoromethoxy)pyridin-3-amine (S6)

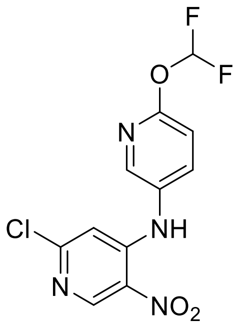

General procedure A; yield 54%. (1.60 g (est. 50% product), 2.53 mmol); dark brown oil. ^{1}H NMR (400 MHz, $CDCl_3$) δ 9.50 (s, 2H), 9.12 (s, 1H), 8.20 (dt, J = 2.8, 0.7 Hz, 1H), 7.71 – 7.68 (m, 1H), 7.48 (t, J = 76.7 Hz, 1H), 6.72 (s, 1H); *m/z* (ESI$^+$) 317.0 ([M+H]$^+$, 100%).

6-Chloro-_N_^{4}-(6-(difluoromethoxy)pyridin-3-yl)pyridine-3,4-diamine (S7)

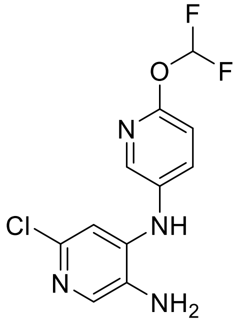

General procedure B.1 from **S6**; yield 61% (1.17 g, 2.86 mmol); dark brown thick oil. ^{1}H NMR (400 MHz, $CDCl_3$) δ 8.05 (d, J = 2.7 Hz, 1H), 7.83 (s, 1H), 7.58 (dd, J = 8.7, 2.6 Hz, 1H), 7.42 (d, J = 72.6 Hz, 1H), 6.97 (d, J = 8.6 Hz, 1H), 6.71 (s, 1H), 6.02 (s, 1H), 3.28 (s, 2H); m/z (ESI+) 287.0 ([M+H]+, 100%).

6-Chloro-1-(6-(difluoromethoxy)pyridin-3-yl)-1*H*-imidazo[4,5-*c*]pyridine (S8)

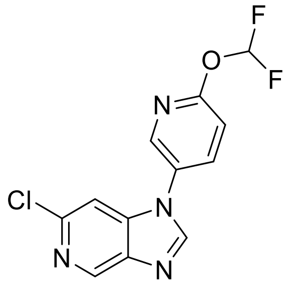

General procedure C from **S7**; yield 76% (913 mg, 3.08 mmol); dark brown solid.^{1}H NMR (400 MHz, $CDCl_3$) δ 8.97 (d, J = 0.9 Hz, 1H), 8.39 (dd, J = 2.8, 0.7 Hz, 1H), 8.10 (s, 1H), 7.88 (dd, J = 8.7, 2.8 Hz, 1H), 7.52 (t, J = 72.1 Hz, 1H), 7.39 (d, J = 1.0 Hz, 1H), 7.19 (dd, J = 8.7, 0.7 Hz, 1H); m/z (ESI+) 297.0 ([M+H]+, 100%).

4-(1-(6-(Difluoromethoxy)pyridin-3-yl)-1*H*-imidazo[4,5-*c*]pyridin-6-yl)-3-methylbenzonitrile (14)

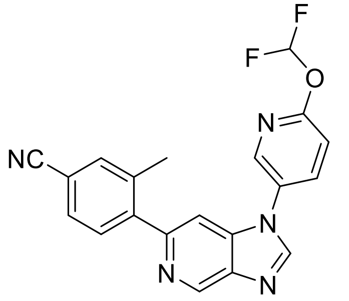

General procedure D from **S8** (92 mg, 0.31 mmol); yield 49% (58 mg, 0.15 mmol); beige solid. ^{1}H NMR (500 MHz, $CDCl_3$) δ 9.30 (d, *J* = 0.8 Hz, 1H), 8.43 (d, *J* = 2.7 Hz, 1H), 8.18 (s, 1H), 7.91 (dd, *J* = 8.7, 2.8 Hz, 1H), 7.59 (s, 1H), 7.56 (d, *J* = 7.9 Hz, 1H), 7.51 (t, *J* = 72.2 Hz, 1H), 7.49 (d, *J* = 7.9 Hz, 1H), 7.44 (d, *J* = 0.8 Hz, 1H), 7.18 (d, *J* = 8.7 Hz, 1H), 2.39 (s, 3H); ^{13}C NMR (126 MHz, $CDCl_3$) δ 159.1 (t, *J* = 4 Hz), 152.9, 145.1, 144.0, 143.4, 142.9, 140.2, 139.4, 137.9, 136.4, 134.4, 130.7, 129.7, 128.9, 119.0, 114.0 (t, *J* = 258 Hz), 113.3, 112.1, 105.4, 20.5; *m/z* (ESI^+) 378.1 ($[M+H]^+$, 100 %); HRMS (ESI^+) $C_{20}H_{14}ON_5F_5$ ($[M+H]^+$) requires 378.11627; found 378.11609.

N-(2-Chloro-5-nitropyridin-4-yl)-1,3-dimethyl-1H-indazol-5-amine (S9)

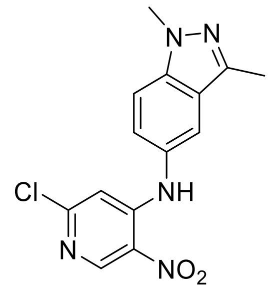

General procedure A; yield quant. (1.65 g, 5.19 mmol); dark-brown solid. ^{1}H NMR (400 MHz, Chloroform-d) δ 9.75 (s, 1H), 9.11 (s, 1H), 7.76 (dd, J = 8.4, 0.7 Hz, 1H), 7.24 (dt, J = 1.6, 0.8 Hz, 1H), 7.01 (dd, J = 8.4, 1.7 Hz, 1H), 6.91 (s, 1H), 4.02 (s, 3H), 2.60 (s, 3H); m/z (ESI+) 318.1 ([M+H]+, 100%).

6-Chloro-N^4-(1,3-dimethyl-1H-indazol-5-yl)pyridine-3,4-diamine (S10)

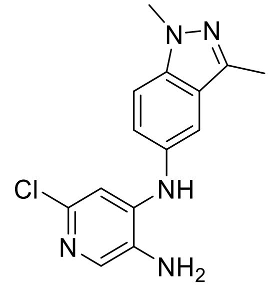

General procedure B.1 from **S9**; yield 87% (1.29 g, 4.48 mmol); red-brown solid. ^{1}H NMR (400 MHz, Chloroform-d) δ 7.82 (s, 1H), 7.62 (dd, J = 8.4, 0.7 Hz, 1H), 6.99 (d, J = 1.8 Hz, 2H), 6.90 (dd, J = 8.5, 1.8 Hz, 1H), 6.17 (s, 1H), 3.95 (s, 3H), 3.33 (s, 2H), 2.55 (s, 3H); m/z (ESI+) 288.1 ([M+H]+, 100%).

6-Chloro-1-(1,3-dimethyl-1H-indazol-5-yl)-1H-imidazo[4,5-c]pyridine (S11)

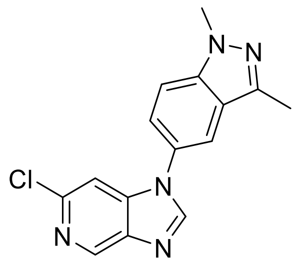

General procedure C from **S10**; yield 97% (1.29 g, 4.33 mmol); dark red solid.^{1}H NMR (400 MHz, Chloroform-d) δ 8.95 (d, J = 0.9 Hz, 1H), 8.22 (s, 1H), 7.86 (dd, J = 8.4, 0.7 Hz, 1H), 7.49

(d, J = 0.9 Hz, 1H), 7.40 (dd, J = 1.7, 0.7 Hz, 1H), 7.19 (dd, J = 8.4, 1.8 Hz, 1H), 4.07 (s, 3H), 2.63 (s, 3H); m/z (ESI+) 298.1 ([M+H]+, 100%).

1-(1,3-Dimethyl-1*H*-indazol-5-yl)-6-(4-fluoro-2-methylphenyl)-1*H*-imidazo[4,5-*c*]pyridine (15)

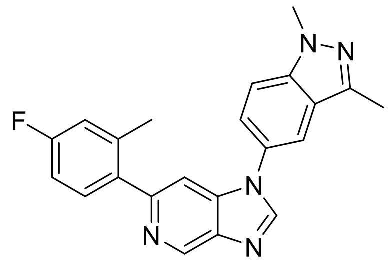

General procedure D from **S11** (93 mg, 0.31 mmol) and 5-fluoro-2-methylphenylboronic acid (58 mg, 0.37 mmol); yield 73% (85 mg, 0.23 mmol); red-brown solid. ^{1}H NMR (500 MHz, $CDCl_3$) δ 9.27 (s, 1H), 8.27 (s, 1H), 7.85 (d, *J* = 8.4 Hz, 1H), 7.53 (d, *J* = 0.9 Hz, 1H), 7.43 (d, *J* = 1.3 Hz, 1H), 7.37 (dd, *J* = 8.4, 6.0 Hz, 1H), 7.26-7.24 (m, 1H), 7.00 (dd, *J* = 9.8, 2.6 Hz, 1H), 6.95 (td, *J* = 8.4, 2.6 Hz, 3H), 4.05 (s, 3H), 2.63 (s, 3H), 2.37 (s, 3H); ^{13}C NMR (126 MHz, $CDCl_3$) δ 162.6 (d, *J* = 247 Hz), 153.4, 144.4, 142.8, 142.2, 141.0, 140.0, 139.6, 138.8 (d, *J* = 8 Hz), 137.1, 133.8, 131.7 (d, *J* = 8 Hz), 123.4, 122.8, 117.4 (d, *J* = 21 Hz), 116.3, 112.8 (d, *J* = 21 Hz), 106.0, 104.6, 35.7, 20.8, 12.0; *m/z* (ESI^+) 372.1 ($[M+H]^+$, 100%); HRMS (ESI) $C_{22}H_{19}N_5F$ ($[M+H]^+$) requires 372.1621; found 372.1619.

***N*-(2-Chloro-5-nitropyridin-4-yl)-1-methyl-1*H*-indazol-6-amine (S12)**

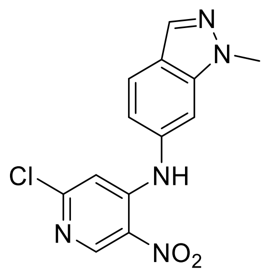

General procedure A; yield 86% (678 mg, 2.23 mmol); brown solid; ^{1}H NMR ($CDCl_3$, 400 MHz) δ 9.82 (bs, 1H), 9.08 (s, 1H), 8.04 (s, 1H), 7.85 (d, *J* = 8.8 Hz, 1H), 7.39 (s, 1H), 7.07 (d, *J* = 8.4 Hz, 1H), 6.91 (s, 1H), 4.10 (s, 3H); ^{13}C NMR ($CDCl_3$, 100 MHz) δ 13C NMR (101 MHz, CDCl3) δ 156.2, 148.9, 146.4, 140.0, 134.1, 132.8, 129.5, 123.0, 122.8, 118.5, 108.1, 106.0, 35.5; *m/z* (ESI^+) 304.1 ($[M+H]^+$, 100%).

6-Chloro-N^4-(1-methyl-1*H*-indazol-6-yl)pyridine-3,4-diamine (S13)

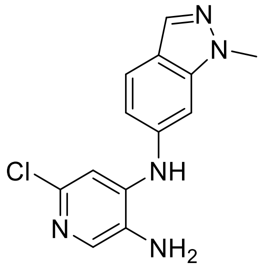

General procedure B.2 from **S12** (660 mg, 2.17 mmol); yield 67% (400 mg, 1.46 mmol); brown solid. ^{1}H NMR (DMSO-d_6, 400 MHz) δ 7.95 (d, *J* = 0.8 Hz, 1H), 7.86 (br s, 1H), 7.72 (d, *J* = 8.4 Hz, 1H), 7.66 (s, 1H), 7.28 (s, 1H), 7.00 - 6.98 (m, 1H), 6.84 (s, 1H), 5.02 (br s, 2H), 3.98 (s, 3H); ^{13}C NMR (DMSO-d_6, 100 MHz) δ 141.0, 140.6, 139.7, 139.2, 135.1, 133.5, 132.9, 122.1, 120.5, 116.6, 107.3, 100.2, 35.7; *m/z* (ESI$^+$) 274.1 ([M+H]$^+$, 100%).

6-Chloro-1-(1-methyl-1*H*-indazol-6-yl)-1*H*-imidazo[4,5-*c*]pyridine (S14)

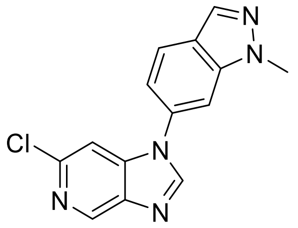

General procedure C from **S13** (390 mg, 1.42 mmol); yield 81% (330 mg, 1.16 mmol); beige solid. ^{1}H NMR (DMSO-d_6, 400 MHz) δ 8.91 (s, 1H), 8.81 (s, 1H), 8.19 (s, 1H), 8.05 (s, 1H), 8.01 (d, *J* = 8.8 Hz, 1H), 7.81 (s, 1H), 7.44 (dd, *J* = 8.4, 1.6 Hz, 1H), 4.13 (s, 3H); ^{13}C NMR (DMSO-d_6, 100 MHz) δ 147.2, 143.9, 142.1, 141.2, 141.1, 140.3, 133.3, 133.2, 123.7, 123.1, 117.4, 106.7, 106.1, 36.1; *m/z* (ESI$^+$) 284.1 ([M+H]$^+$, 100%).

6-[6-(4-Fluoro-2-methylphenyl)-1*H*-imidazo[4,5-*c*]pyridin-1-yl]-1-methyl-1*H*-indazole (16)

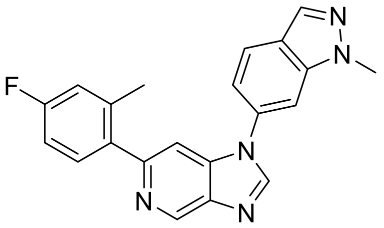

General procedure D from **S14** (150 mg, 0.529 mmol); yield 20% (38 mg, 0.11 mmol); white solid. ^{1}H NMR (DMSO-d_6, 400 MHz) δ 9.15 (d, *J* = 0.8 Hz, 1H), 8.80 (s, 1H), 8.18 (s, 1H), 8.07 (s, 1H), 8.00 (d, *J* = 8.4 Hz, 1H), 7.75 (s, 1H), 7.51 - 7.48 (m, 2H), 7.15 (dd, *J* = 10.4, 2.8 Hz, 1H), 7.07

(dt, J = 8.4, 2.4 Hz, 1H), 4.11 (s, 3H), 2.36 (s, 3H); ^{13}C NMR (DMSO-d_6, 100 MHz) δ161.5 (d, J = 244 Hz), 161.3 (d, J = 4 Hz)151.9, 145.6, 141.3, 139.6 (d, J = 9 Hz), 138.6, 138.5 (d, J = 8 Hz), 137.1 (d, J = 3 Hz), 133.0, 132.7, 131.8 (d, J = 8 Hz), 122.8, 122.5, 116.8, 116.7 (d, J = 21 Hz), 112.2 (d, J = 21 Hz), 106.1, 105.2, 35.5, 20.2; m/z (ESI$^+$) 358.1 ([M+H]$^+$, 100%); HRMS (ESI) $C_{21}H_{17}N_5F$ ([M+H]$^+$) requires 358.1463; found 358.1470.

N-(2-Chloro-5-nitropyridin-4-yl)-3-methyl-1H-indazol-5-amine (S15)

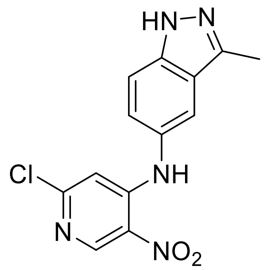

General procedure A; yield 87% (688 mg, 2.27 mmol); red solid. ^{1}H NMR (DMSO-d_6, 400 MHz) δ 12.7 (bs, 1H), 9.97 (s, 1H), 8.96 (s, 1H), 7.71 (d, J = 1.2 Hz, 1H), 7.58 (d, J = 8.8 Hz, 1H), 7.29 (dd, J = 8.8, 1.6 Hz, 1H), 6.61 (s, 1H), 2.49 (s, 3H). ^{13}C NMR (DMSO-d_6, 100 MHz) δ 155.0, 150.0, 149.3, 142.1, 140.2, 130.4, 129.2, 125.6, 123.2, 118.5, 111.9, 108.6, 12.1; m/z (ESI$^+$) 304.0 ([M+H]$^+$, 100%).

6-Chloro-N^4-(3-methyl-1H-indazol-5-yl)pyridine-3,4-diamine (S16)

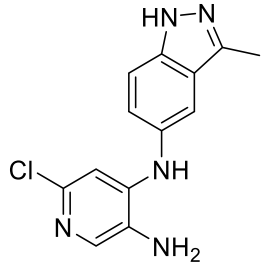

General procedure B.2 from **S15** (0.67 g, 2.21 mmol); yield 61% (370 mg, 1.35 mmol); yellow solid. ^{1}H NMR (DMSO-d_6, 400 MHz) δ 12.89 (br s, 1H), 7.64 (s, 1H), 7.58 (s, 1H), 7.50 (d, J = 8.8 Hz, 1H), 7.46 (s, 1H), 7.20 (dd, J = 1.6, 8.8 Hz, 1H), 6.44 (s, 1H), 4.92 (br s, 2H), 2.47 (s, 3H); ^{13}C NMR (DMSO-d_6, 100 MHz) δ 143.0, 141.3, 139.6, 139.1, 134.3, 132.5, 132.2, 124.2, 123.2, 114.2, 111.5, 104.9, 12.1; m/z (ESI$^+$) 274.0 ([M+H]$^+$, 100%).

6-Chloro-1-(1-(diethoxymethyl)-3-methyl-1*H*-indazol-5-yl)-1*H*-imidazo[4,5-*c*]pyridine (S17)

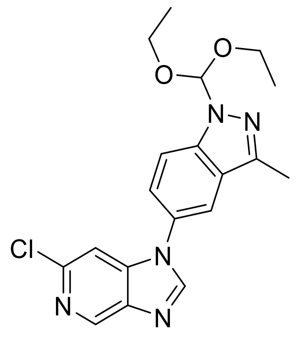

General procedure C from **S16** (360 mg, 1.32 mmol); yield 90% (458 mg, 1.19 mmol); orange solid. ^{1}H NMR (DMSO-d_6, 400 MHz) δ 8.90 (s, 1H), 8.75 (s, 1H), 8.11 (d, *J* = 1.6 Hz, 1H), 7.95 (d, *J* = 8.8 Hz 1H), 7.71-7.67 (m, 2H), 6.50 (s, 1H), 3.77-3.69 (m, 2H), 3.59-3.50 (m, 2H), 2.55 (s, 3H), 1.17 (t, *J* = 7.2 Hz, 6H); ^{13}C NMR (DMSO-d_6, 100 MHz) δ 147.5, 143.7, 143.0, 142.0, 141.3, 141.0, 138.4, 128.5, 125.0, 124.0, 117.1, 113.5, 106.5, 106.3, 62.5, 15.2, 12.1; *m/z* (ESI$^-$) 282.1 ([M-CHOEt$_2$]$^+$, 100%).

5-[6-(4-Fluoro-2-methylphenyl)-1*H*-imidazo[4,5-*c*]pyridin-1-yl]-3-methyl-1*H*-indazole (17)

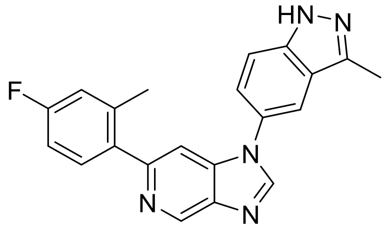

General procedure D from **S17** (0.15 g, 0.39 mmol), then the resulting crude product was dissolved in MeCN (3 mL), TFA (140 µL, 1.94 mmol) was added and stirred at room temperature overnight. The mixture was basified (NaOH) and concentrated *in vacuo*. The aqueous layer was extracted with DCM, the combined organic layers dried over anhydrous Na_2SO_4, then evaporated to dryness and triturated with Et_2O to give **OXS008185** (61 mg, 0.17 mmol, 44%) as an off-white solid. ^{1}H NMR ($CDCl_3$, 400 MHz) δ 10.48 (bs, 1H), 9.27 (d, *J* = 0.8 Hz, 1H), 8.23 (s, 1H), 7.79 (d, *J* = 1.2 Hz, 1H), 7.60 (d, *J* = 8.8 Hz, 1H), 7.50-7.45 (m, 2H), 7.37 (dd, *J* = 8.4, 6.0 Hz, 1H), 6.98 (dd, *J* = 10.0, 2.8 Hz, 1H), 6.93 (dt, *J* = 8.4, 2.8 Hz, 1H), 2.64 (s, 3H), 2.36 (s, 3H); ^{13}C NMR ($CDCl_3$, 100 MHz) δ ^{13}C NMR (101 MHz, $CDCl_3$) δ 162.5 (d, *J* = 247 Hz), 153.2, 144.6, 144.0, 142.6, 140.5, 139.9, 139.7, 138.7 (d, *J* = 8 Hz), 137.0 (d, *J* = 3 Hz), 131.5 (d, *J* = 8 Hz), 128.0, 123.6, 123.4, 117.2 (d, *J* = 21 Hz), 116.5, 112.6 (d, *J* = 21 Hz), 111.5, 105.7, 20.6, 12.0; *m/z* (ESI$^+$) 385.1 ([M+H]$^+$, 100%); HRMS (ESI$^+$) $C_{21}H_{17}N_5F$ ([M+H]$^+$) requires 358.1463; found 358.1473.

_N_-(2-Chloro-5-nitropyridin-4-yl)-1_H_-indazol-5-amine (S18)

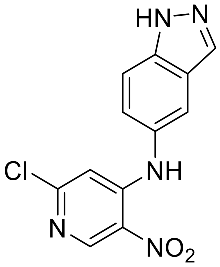

General procedure A; yield 93% (700 mg, 2.42 mmol); orange solid. ^{1}H NMR (DMSO-d_6, 400 MHz) δ 13.21 (bs, 1H), 9.99 (s, 1H), 8.96 (s, 1H), 8.13 (s, 1H), 7.77 (d, J = 1.2 Hz, 1H), 7.67 (d, J = 8.8 Hz, 1H), 7.31 (dd, J = 1.6, 8.4 Hz, 1H), 6.63 (s, 1H); ^{13}C NMR (DMSO-d_6, 100 MHz) δ 155.0, 149.9, 149.3, 139.3, 134.4, 130.5, 129.9, 125.6, 123.8, 118.8, 112.0, 105.5; *m/z* (ESI$^+$) 289.9 ([M+H]$^+$, 100%).

6-Chloro-_N_-(1_H_-indazol-5-yl)pyridine-3,4-diamine (S19)

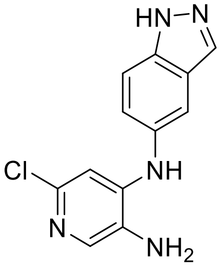

General procedure B.2 from **S18** (680 mg, 2.35 mmol); yield 66% (400 mg, 1.54 mmol); pale orange solid. ^{1}H NMR (DMSO-d_6, 400 MHz) δ 13.02 (br s, 1H), 8.04 (s, 1H), 7.66 (s, 1H), 7.59-7.57 (m, 2H), 7.54 (d, J = 1.6 Hz, 1H), 7.22 (dd, J = 2.0, 8.8 Hz, 1H), 6.50 (s, 1H), 4.94 (br s, 2H); ^{13}C NMR (DMSO-d_6, 100 MHz) δ 142.7, 139.6, 138.1, 134.4, 133.7, 133.3, 132.3, 124.0, 123.9, 114.0, 111.6, 105.0; *m/z* (ESI$^+$) 260.0 ([M+H]$^+$, 100%).

6-Chloro-1-(1-(diethoxymethyl)-1H-indazol-5-yl)-1H-imidazo[4,5-c]pyridine (S20)

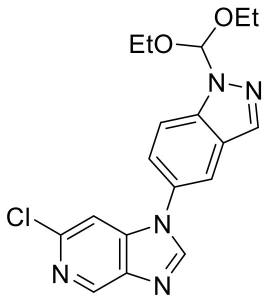

General procedure C from **S19** (390 mg, 1.50 mmol); yield 76% (425 mg, 1.14 mmol); off-white solid. ^{1}H NMR (DMSO-d_6, 400 MHz) δ 8.89 (s, 1H), 8.76 (s, 1H), 8.27 (s, 1H), 8.15 (d, J = 1.6 Hz, 1H), 8.02 (d, J = 8.8 Hz,1H), 7.73-7.70 (m, 2H), 6.61 (s, 1H), 3.79-3.72 (m, 2H), 3.60-3.52 (m, 2H), 1.17 (t, J = 7.2 Hz, 6H); ^{13}C NMR (DMSO-d_6, 100 MHz) δ 147.4, 143.7, 142.0, 141.3, 141.0, 137.5, 134.9, 129.1, 125.5, 124.0, 117.4, 113.7, 106.6, 106.5, 62.6, 15.2; *m/z* (ESI$^+$) 372.0 ([M+H]$^+$, 100%).

5-[6-(4-Fluoro-2-methylphenyl)-1*H*-imidazo[4,5-*c*]pyridin-1-yl]-1H-indazole (18)

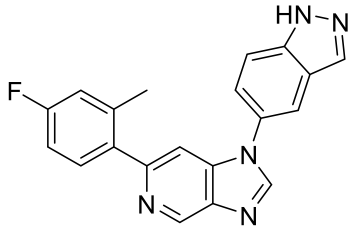

General procedure D from **S20** (150 mg, 0.40 mmol), then the product was dissolved in DCM and TFA (200 μL) and stirred overnight. The solution was then basified with saturated aqueous $NaHCO_3$ and stirred for 1 h. The resulting precipitate was filtered and washed with water, then hexane. The solid was dried in a vacuum oven at 60°C overnight to give the product (32 mg, 0.093 mmol, 23%) as an off-white solid. ^{1}H NMR (DMSO-d_6, 400 MHz) δ 13.32 (br s, 1H), 9.13 (d, J = 1.2 Hz, 1H), 8.73 (s, 1H), 8.21 (s, 1H), 8.13 (d, J =1.2 Hz, 1H), 7.78 (d, J = 8.8 Hz, 1H), 7.68 (dd, J = 2.0, 8.8 Hz, 1H), 7.61 (d, J = 0.8 Hz, 1H), 7.46 (dd, J = 2.0,8.4 Hz, 1H), 7.15-7.12 (m, 1H), 7.06 (td, J = 2.8, 8.8 Hz, 1H), 2.34 (s, 3H); ^{13}C NMR (101 MHz, DMSO-d_6) δ 161.4 (d, J = 244 Hz), 151.8, 145.7, 141.2, 139.4, 139.0, 138.9, 138.5 (d, J = 8 Hz), 137.2 (d, J = 3 Hz), 134.1, 131.6 (d, J = 8 Hz), 127.9, 123.0, 122.7, 116.6 (d, J = 21 Hz), 116.1, 112.1 (d, J = 21 Hz), 111.5, 105.8, 20.1; *m/z* (ESI$^+$) 344.0 ([M+H]$^+$, 100%); HRMS (ESI$^+$) $C_{20}H_{15}N_5F$ ([M+H]$^+$) requires 344.1306; found 344.1297.

***N*-(2-Chloro-5-nitropyridin-4-yl)-3-methyl-1*H*-indazol-6-amine (S21)**

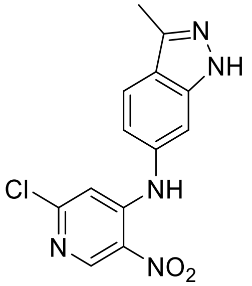

General procedure A; yield 100% (918 mg, 3.02 mmol); yellow solid. ^{1}H NMR (DMSO-d_6, 400 MHz) δ 12.74 (bs, 1H), 10.02 (s, 1H), 8.97 (s, 1H), 7.81 (d, J = 8.8 Hz, 1H), 7.46 (s, 1H), 7.06 (dd, J = 8.4, 1.6 Hz, 1H), 6.78 (s, 1H), 2.51 (s, 3H). ^{13}C NMR (DMSO-d_6, 100 MHz) δ 155.0, 149.3,

149.2, 141.9, 141.7, 135.4, 130.8, 121.8, 121.6, 118.5, 108.9, 107.6, 12.1; *m/z* (ESI^+) 304.0 ($[M+H]^+$, 100%).

6-Chloro-N^4-(3-methyl-1*H*-indazol-6-yl)pyridine-3,4-diamine (S22)

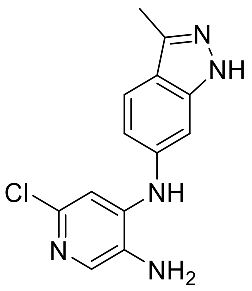

General procedure B.2 from **S21** (303 mg, 2.47 mmol); yield 66% (450 mg, 1.64 mmol); orange solid. ^{1}H NMR (DMSO-d_6, 400 MHz) δ 12.35 (s, 1H), 7.82 (s, 1H), 7.50 (dd, *J* = 8.4 Hz, 1H), 7.65 (s, 1H), 7.14 (d, *J* = 1.6 Hz, 1H), 6.94-6.91 (m, 1H), 6.83 (s, 1H), 5.03 (s, 2H), 2.45 (s, 3H); ^{13}C NMR (DMSO-d_6, 100 MHz) δ 142.2, 141.6, 140.6, 139.5, 139.1, 135.0, 133.5, 121.3, 119.2, 115.5, 107.1, 100.2, 12.1; *m/z* (ESI^+) 274.1 ($[M+H]^+$, 100%).

6-Chloro-1-(1-(diethoxymethyl)-3-methyl-1*H*-indazol-6-yl)-1*H*-imidazo[4,5-*c*]pyridine (S23)

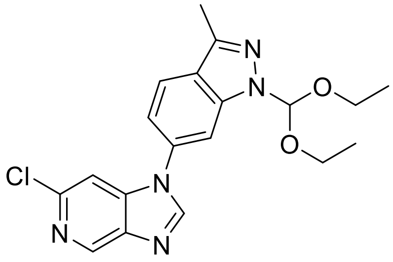

General procedure C from **S22** (450 mg, 1.64 mmol); yield 66% (420 mg, 1.09 mmol); brown solid. ^{1}H NMR (DMSO-d_6, 400 MHz) δ 8.92 (d, *J* = 0.8 Hz, 1H), 8.83 (s, 1H), 8.00 (d, *J* = 8.4 Hz, 1H), 7.97 (d, *J* = 1.2 Hz, 1H), 7.61 (d, *J* = 0.8 Hz, 1H), 7.50 (dd, *J* = 2, 8.4 Hz, 1H), 6.52 (s, 1H), 3.75-3.68 (m, 2H), 3.60-3.49 (m, 2H), 2.57 (s, 3H), 1.18-1.14 (m, 6H); ^{13}C NMR (DMSO-d_6, 100 MHz) δ 147.2, 143.8, 142.8, 142.3, 141.2, 141.0, 139.1, 133.8, 124.2, 122.9, 117.8, 107.0, 106.3, 105.9, 62.4, 15.2, 12.0; *m/z* (ESI^-) 282.1 ($[M-CHOEt_2]^+$, 100%).

1-(1-(Diethoxymethyl)-3-methyl-1*H*-indazol-6-yl)-6-(4-fluoro-2-methylphenyl)-1*H*-imidazo[4,5-*c*]pyridine (S24)

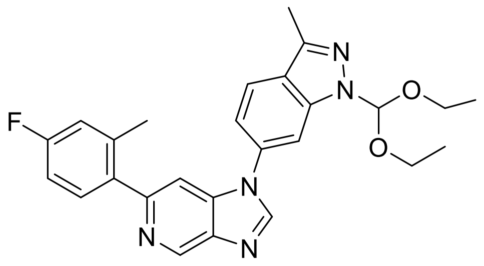

General procedure D from **S23** (150 mg, 0.39 mmol); yield 59% (106 mg, 0.231 mmol); beige solid. ^{1}H NMR ($CDCl_3$, 400 MHz) δ 9.26 (d, *J* = 0.8 Hz, 1H), 8.29 (s, 1H), 7.99 (d, *J* = 1.2 Hz, 1H), 7.83 (d, *J* = 8.8 Hz, 1H), 7.56 (d, *J* = 1.2 Hz, 1H), 7.38 (dd, *J* = 8.4, 6.2 Hz, 1H), 7.31 (dd, *J* = 8.8, 2.0 Hz, 1H), 6.98 (dd, *J* = 9.6, 2.8 Hz, 1H), 6.92 (td, *J* = 8.4, 2.8 Hz, 1H), 6.36 (s, 1H), 3.79-3.71 (m, 2H), 3.58-3.50 (m, 2H), 2.62 (s, 3H), 2.38 (s, 3H), 1.19 (t, *J* = 7.2 Hz, 6H); ^{13}C NMR ($CDCl_3$, 100 MHz) δ 13C NMR (101 MHz, CDCl3) δ 162.6 (d, J = 246 Hz), 153.5, 144.2, 143.0, 142.9, 140.1, 139.4, 139.0, 138.8 (d, J = 8 Hz), 137.2 (d, J = 3 Hz), 134.1, 131.7 (d, J = 8 Hz), 124.7, 122.4, 117.3, 117.3 (d, *J* = 21 Hz), 112.7 (d, J = 21 Hz), 107.7, 107.1, 106.2, 63.0, 20.7, 14.9, 12.0; *m/z* (ESI$^-$) 356.1 ([M-CHOEt$_2$]$^+$, 100%).

6-[6-(4-Fluoro-2-methylphenyl)-1*H*-imidazo[4,5-*c*]pyridin-1-yl]-3-methyl-1*H*-indazole (19)

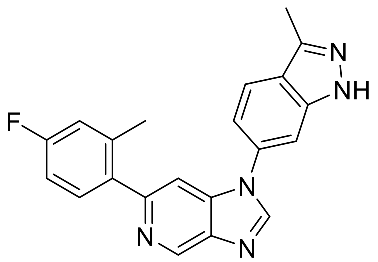

S24 (51 mg, 0.11 mmol) was suspended in MeCN (3.0 mL), TFA (140 µL, 1.94 mmol) was added and the solution was stirred at room temperature overnight. The mixture was basified with aqueous NaOH and concentrated. The aqueous layer was then extracted with DCM, dried through a phase separator, and triturated with Et_2O/hexane to give **19** (34 mg, 0.10 mmol, 24%) as a white solid. ^{1}H NMR ($CDCl_3$, 400 MHz) δ 10.28 (bs, 1H), 9.27 (d, *J* = 0.8 Hz, 1H), 8.26 (s, 1H), 7.89 (d, *J* = 8.4 Hz, 1H), 7.53 (d, *J* = 0.8 Hz, 2H), 7.36 (dd, *J* = 8.4, 6.0 Hz, 1H), 7.29 (dd, *J* = 8.4, 2.0 Hz, 1H), 6.98 (dd, *J* = 9.6, 2.8 Hz, 1H) 6.92 (dt, *J* = 8.4, 2.8 Hz, 1H), 2.66 (s, 3H), 2.35 (s, 3H); ^{13}C NMR ($CDCl_3$, 100 MHz) δ 162.6 (d, J = 247 Hz), 153.5, 144.3, 144.1, 142.9, 141.3, 140.0, 139.5, 138.8 (d, J = 8 Hz), 137.1 (d, J = 3 Hz), 134.1, 131.6 (d, J = 8 Hz), 123.0, 122.6, 117.4 (d, J = 21 Hz),

116.9, 112.8 (d, J = 21 Hz), 106.0, 105.3, 20.7, 12.1; *m/z* (ESI$^+$) 385.1 ([M+H]$^+$, 100%); HRMS (ESI$^+$) $C_{21}H_{17}N_5F^+$ ([M+H]$^+$) requires 358.1463; found 358.1469.

3-Methyl-4-[1-(3-methyl-1H-indazol-6-yl)imidazo[4,5-c]pyridin-6-yl]benzonitrile (OXS008255, 20)

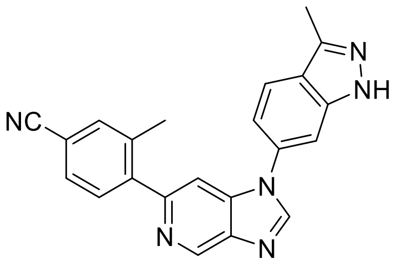

General procedure D from **S23** (75 mg, 0.19 mmol); yield 57% (52 mg, 0.11 mmol); white solid. To a solution of the resulting material (30 mg, 0.06 mmol) in ethanol (1.2 mL) was added HCl (2 M, 0.11 mL, 0.21 mmol) at 0 °C. After stirring for 30 min at room temperature, the pH of the mixture was adjusted to 14 by addition of aqueous NaOH (2 M). After a further 30 min, H_2O was added, and the mixture was extracted with 3xEtOAc. The organic layer was dried over anhydrous Na_2SO_4, evaporated, and purified by flash column chromatography (100% EtOAc in pentane) to give product **20** (5 mg, 0.01 mmol, 21%). ^{1}H NMR (500 MHz, $CDCl_3$) δ 9.29 (d, *J* = 1.1 Hz, 2H), 8.30 (s, 1H), 7.90 (d, *J* = 8.4 Hz, 1H), 7.61 – 7.55 (m, 3H), 7.55 – 7.47 (m, 2H), 7.30 (dd, *J* = 8.4, 1.8 Hz, 1H), 2.66 (s, 3H), 2.40 (s, 3H); ^{13}C NMR (126 MHz, $CDCl_3$) δ 152.4, 145.4, 144.7, 144.2, 143.2, 141.2, 140.4, 139.5, 137.9, 134.4, 134.0, 130.8, 129.7, 123.0, 122.8, 119.0, 116.9, 111.9, 106.1, 105.4, 20.5, 12.2; *m/z* (ESI$^+$) 365.1 ([M+H]$^+$, 100%); HRMS (ESI$^+$) $C_{22}H_{17}N_6^+$ ([M+H]$^+$) requires 365.1509; found 365.1512.

***N*-(2-Chloro-5-nitropyridin-4-yl)-2-methylbenzo[*d*]oxazol-6-amine (S25)**

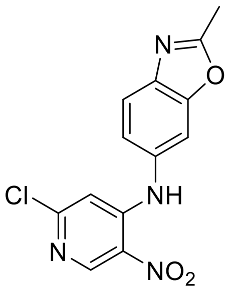

General procedure A; yield 100% (842 mg, 2.77 mmol); yellow solid. ^{1}H NMR ($CDCl_3$, 400 MHz) δ 9.74 (s, 1H), 9.09 (s, 1H), 7.75 (d, *J* = 8.4 Hz, 1H), 7.46 (d, *J* = 1.6 Hz, 1H), 7.24 (dd, *J* = 8.4, 1.6 Hz, 1H), 6.83 (d, *J* = 2 Hz, 1H), 2.70 (s, 3H); ^{13}C NMR ($CDCl_3$, 100 MHz) δ 165.5, 156.5,

151.4, 149.2, 149.1, 141.2, 132.5, 129.6, 122.4, 120.6, 108.4, 108.1, 14.6; *m/z* (ESI^+) 305.0 ($[M+H]^+$, 100%).

6-Chloro-*N*^{4}-(2-methylbenzo[*d*]oxazol-6-yl)pyridine-3,4-diamine (S26)

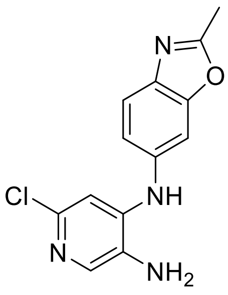

General procedure B.2 from **S25** (790 mg, 2.59 mmol); yield 46% (330 mg, 1.20 mmol); orange solid. ^{1}H NMR (400 MHz, DMSO-d_6) δ 7.85 (s, 1H), 7.64 (s, 1H), 7.61 (d, J = 8.8 Hz, 1H), 7.44 (d, J = 2.0 Hz, 1H), 7.15 (dd, J = 8.4, 2.0 Hz, 1H), 6.73 (s, 1H), 5.01 (br s, 2H), 2.56 (s, 3H); ^{13}C NMR (101 MHz, DMSO-d_6) δ 163.9, 151.6, 140.9, 139.3, 138.2, 137.5, 134.9, 133.3, 119.9, 118.9, 106.5, 103.7, 14.5; *m/z* (ESI^+) 275.1 ($[M+H]^+$, 100%).

6-(6-Chloro-1*H*-imidazo[4,5-*c*]pyridin-1-yl)-2-methylbenzo[*d*]oxazole (S27)

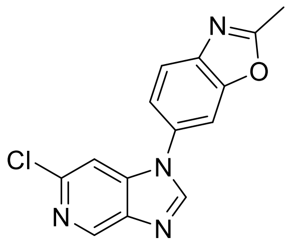

General procedure C from **S26** (315 mg, 1.15 mmol); yield 88% (287 mg, 1.01 mmol); light brown solid. ^{1}H NMR (400 MHz, DMSO-d_6) δ 8.90 (s, 1H), 8.78 (s, 1H), 8.13 (d, J = 2 Hz, 1H), 7.88 (d, J = 8.4 Hz, 1H), 7.73 (s, 1H), 7.66 (dd, J = 8.4, 2.0 Hz, 1H), 2.68 (s, 3H); ^{13}C NMR (101 MHz, DMSO-d_6) δ 166.3, 151.1, 147.4, 143.8, 142.0, 141.7, 141.0, 131.7, 121.3, 120.5, 107.8, 106.6, 14.7; *m/z* (ESI^+) 285.1 ($[M+H]^+$, 100%).

6-(6-(4-Fluoro-2-methylphenyl)-1*H*-imidazo[4,5-*c*]pyridin-1-yl)-2-methylbenzo[*d*]oxazole (21)

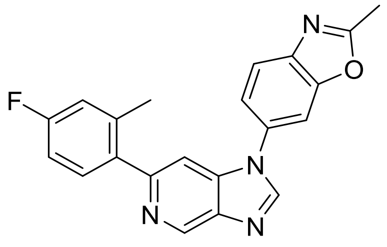

General procedure D from **S27** (150 mg, 0.527 mmol); yield 8% (14 mg, 0.040 mmol); yellow solid. ^{1}H NMR (400 MHz, DMSO-d_6) δ 9.13 (d, *J* = 0.9 Hz, 1H), 8.76 (s, 1H), 8.16 (d, *J* = 1.9 Hz, 1H), 7.88 (d, *J* = 8.4 Hz, 1H), 7.71 (dd, *J* = 8.4, 2.1 Hz, 1H), 7.68 (d, *J* = 0.9 Hz, 1H), 7.49 (dd, *J* = 8.5, 6.2 Hz, 1H), 7.14 (dd, *J* = 10.1, 2.6 Hz, 1H), 7.07 (td, *J* = 8.6, 2.8 Hz, 1H), 2.67 (s, 3H), 2.34 (s, 3H); ^{13}C NMR (101 MHz, DMSO-d_6) δ 166.1, 162.1 (d, *J* = 244.0 Hz), 152.6, 151.2, 146.2, 141.9, 141.4, 140.1, 139.3, 139.2 (d, *J* = 8.1 Hz), 137.7 (d, *J* = 2.9 Hz), 132.3 (d, *J* = 8.5 Hz), 132.2, 121.1, 120.5, 117.3 (d, *J* = 21.0 Hz), 112.8 (d, *J* = 20.9 Hz), 107.6, 106.6, 20.8, 14.6; *m/z* (ESI$^+$) 359.1 ([M+H]$^+$, 100%); HRMS (ESI$^+$) $C_{21}H_{16}N_4OF^+$ ([M+H]$^+$) requires 359.1303; found 359.1317.

***N*-(2-Chloro-5-nitropyridin-4-yl)-2-methylbenzo[*d*]oxazol-5-amine (S28)**

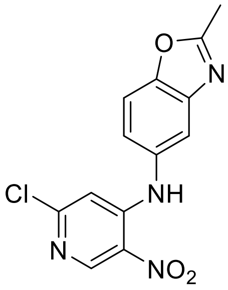

General procedure A; yield 87% (692 mg, 2.27 mmol); brown solid. ^{1}H NMR ($CDCl_3$, 400 MHz) δ 9.68 (bs, 1H), 9.10 (s, 1H), 7.61-7.59 (m, 2H), 7.20 (d, *J* = 1.6 Hz, 1H), 6.80 (s, 1H), 2.70 (s, 3H); ^{13}C NMR ($CDCl_3$, 100 MHz) δ 166.0, 156.7, 150.3, 149.4, 149.3, 143.1, 132.2, 129.6, 122.8, 117.4, 111.7, 108.0, 14.6; *m/z* (ESI$^+$) 305.0 ([M+H]$^+$, 100%).

6-Chloro-N^4-(2-methylbenzo[*d*]oxazol-5-yl)pyridine-3,4-diamine (S29)

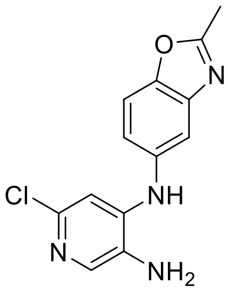

General procedure B.2 from **S28** (0.68 g, 2.23 mmol); yield 52% (320 mg, 1.16 mmol); orange solid. ^{1}H NMR (DMSO-d_6, 400 MHz) δ 7.75 (br s, 1H), 7.63 (d, J = 8.4 Hz, 1H), 7.62 (s, 1H), 7.42 (d, J = 2.0 Hz, 1H), 7.18-7.15 (m, 1H), 6.61 (s, 1H), 4.97 (br s, 2H), 2.60 (s, 3H); ^{13}C NMR (DMSO-d_6, 100 MHz) δ 165.3, 147.6, 142.6, 141.8, 139.4, 137.4, 134.8, 132.8, 120.0, 112.8, 111.4, 105.8, 14.6; *m/z* (ESI$^+$) 275.1 ([M+H]$^+$, 100%).

5-(6-Chloro-1*H*-imidazo[4,5-*c*]pyridin-1-yl)-2-methylbenzo[*d*]oxazole (S30)

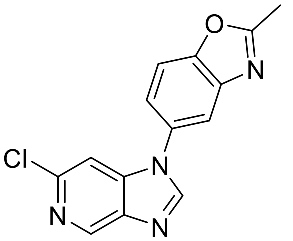

General procedure C from **S29** (310 mg, 1.13 mmol); yield 90% (290 mg, 1.02 mmol); beige solid. ^{1}H NMR (DMSO-d_6, 400 MHz) δ 8.89 (s, 1H), 8.76 (s, 1H), 8.04 (s, 1H), 7.90 (d, J = 8.8 Hz, 1H), 7.68-7.66 (m, 2H), 2.69 (s, 3H); ^{13}C NMR (DMSO-d_6, 100 MHz) δ 166.5, 150.8, 147.3, 143.9, 142.9, 142.0, 141.9, 141.5, 131.7, 121.7, 116.0, 112.0, 106.3, 14.6; *m/z* (ESI$^+$) 285.1 ([M+H]$^+$, 100%).

5-[6-(4-Fluoro-2-methylphenyl)-1*H*-imidazo[4,5-*c*]pyridin-1-yl]-2-methyl-1,3-benzoxazole (22)

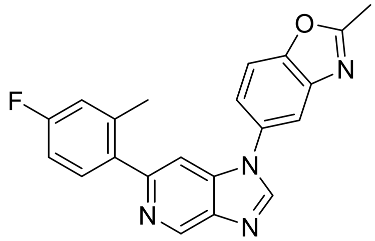

General procedure D from **S30** (150 mg, 0.527 mmol); yield 14% (27 mg, 0.075 mmol); beige solid. ^{1}H NMR (400 MHz, $CDCl_3$) δ 9.25 (d, *J* = 1.0 Hz, 1H), 8.21 (s, 1H), 7.80 (d, *J* = 2.1 Hz, 1H), 7.68 (d, *J* = 8.6 Hz, 1H), 7.47 (d, *J* = 1.0 Hz, 1H), 7.43 (dd, *J* = 8.6, 2.2 Hz, 1H), 7.35 (dd, *J* = 8.4, 6.0 Hz, 1H), 6.98 (dd, *J* = 9.8, 2.6 Hz, 1H), 6.93 (td, *J* = 8.4, 2.7 Hz, 1H), 2.71 (s, 3H), 2.35 (s, 3H); ^{13}C NMR (101 MHz, $CDCl_3$) δ 166.5, 162.6 (d, *J* = 246 Hz), 153.6, 150.9, 144.3, 143.2, 142.9, 139.9, 139.7, 138.8 (d, *J* = 8 Hz), 137.1 (d, *J* = 3 Hz), 131.9, 131.6 (d, *J* = 8 Hz), 121.0, 117.3 (d, *J* = 21 Hz), 115.7, 112.7 (d, *J* = 21 Hz), 111.8, 105.8, 20.7, 14.8; *m/z* (ESI^+) 285.1 ($[M+H]^+$, 100%); HRMS (ESI^+) $C_{21}H_{16}N_4OF^+$ ($[M+H]^+$) requires 359.1303; found 359.1305.

6-Chloro-1-(2-methyl-2H-indazol-6-yl)-1H-imidazo[4,5-c]pyridine (S31)

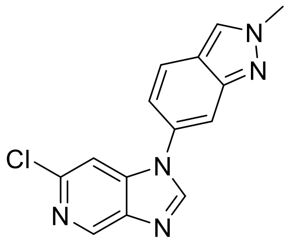

General procedure A, then general procedure B and C; yield over 3 steps 22% (162 mg, 0.570 mmol); pale yellow solid. ^{1}H NMR (500 MHz, $CDCl_3$) δ 8.97 (d, *J* = 0.9 Hz, 1H), 8.24 (s, 1H), 8.08 (s, 1H), 7.90 (dd, *J* = 8.7, 0.8 Hz, 1H), 7.79 (dt, *J* = 1.8, 0.9 Hz, 1H), 7.55 (d, *J* = 0.9 Hz, 1H), 7.17 (dd, *J* = 8.8, 1.9 Hz, 1H), 4.32 (s, 3H); ^{13}C NMR (126 MHz, $CDCl_3$) δ 148.4, 145.0, 144.7, 142.5, 140.9, 140.7, 132.6, 124.5, 122.9, 121.7, 118.2, 112.5, 105.9, 40.8; *m/z* (ESI^+) 284.0 ($[M+H]^+$, 100%); HRMS (ESI^+) $C_{14}H_{11}ClN_5^+$ ($[M+H]^+$) requires 284.0697; found 284.0697.

6-(4-Fluoro-2-methyl-phenyl)-1-(2-methylindazol-6-yl)imidazo[4,5-*c*]pyridine (23)

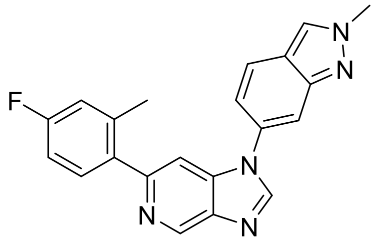

General procedure D from **S31** (75 mg, 0.26 mmol); yield 75% (71 mg, 0.20 mmol); pale yellow solid. ^{1}H NMR (400 MHz, $CDCl_3$) δ 9.25 (s, 1H), 8.27 (s, 1H), 8.04 (s, 1H), 7.86 (d, *J* = 8.7 Hz, 1H), 7.82 (s, 1H), 7.56 (s, 1H), 7.36 (t, *J* = 7.1 Hz, 1H), 7.21 (d, *J* = 8.8 Hz, 1H), 7.01 – 6.89 (m, 2H), 4.28 (s, 3H), 2.35 (s, 3H); ^{13}C NMR (101 MHz, $CDCl_3$) δ 162.4 (d, *J* = 246 Hz), 153.1, 148.4, 144.1, 142.5, 139.8, 139.2, 138.6 (d, *J* = 8 Hz), 137.0 (d, *J* = 3 Hz), 133.0, 131.5 (d, *J* = 8 Hz), 124.4, 122.7, 121.6, 118.3, 117.1 (d, *J* = 21 Hz), 112.5 (d, *J* = 21 Hz), 112.2, 106.0, 40.7, 20.5;

m/z (ESI$^+$) 358.1 ([M+H]$^+$, 100%); HRMS (ESI$^+$) $C_{21}H_{17}FN_5^+$ ([M+H]$^+$) requires 358.1463; found 358.1461.

_N_-(2-Chloro-5-nitropyridin-4-yl)-2,3-dimethyl-2_H_-indazol-5-amine (S32)

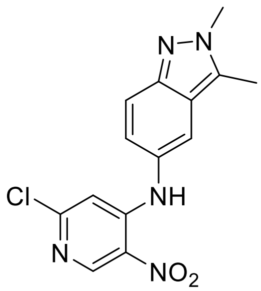

General procedure A; yield 99% (813 mg, 2.56 mmol). ^{1}H NMR (400 MHz, DMSO-d$_6$) δ 10.0 (s, 1H), 8.96 (s, 1H), 7.69 (t, *J* = 0.8 Hz, 1H), 7.63 (d, *J* = 9.2 Hz, 1H), 7.12 (dd, *J* = 2, 8.8 Hz, 1H), 6.61 (s, 1H), 4.07 (s, 3H), 2.61 (s, 3H); ^{13}C NMR (100 MHz, DMSO-d$_6$) δ 154.9, 149.8, 149.4, 146.1, 133.1, 130.3, 129.2, 125.4, 121.1, 118.7, 118.3, 108.6, 37.9, 9.9; *m/z* (ESI$^+$) 318.0 ([M+H]$^+$, 100%).

6-Chloro-_N_^{4}-(2,3-dimethyl-2_H_-indazol-5-yl)pyridine-3,4-diamine (S33)

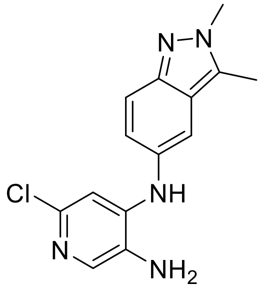

General procedure B.2 from **S32** (800 mg, 2.52 mmol), yield 54% (390 mg, 1.36 mmol); yellow solid. ^{1}H NMR (400 MHz, DMSO-*d*$_6$) δ 7.62 (s, 1H), 7.56 (s, 1H), 7.55 (d, *J* = 9.2 Hz, 1H), 7.39 (d, *J* = 1.6 Hz, 1H), 7.06 (dd, *J* = 2.0, 9.2 Hz, 1H), 6.46 (s, 1H), 4.95 (br s, 2H), 4.04 (s, 3H), 2.58 (s, 3H); ^{13}C NMR (100 MHz, DMSO-*d*$_6$) δ 145.3, 142.7, 139.5, 134.2, 132.4, 132.3, 131.7, 124.6, 121.3, 18.3, 112.9, 105.1, 37.8, 9.9; *m/z* (ESI$^+$) 288.1 ([M+H]$^+$, 100%).

6-Chloro-1-(2,3-dimethyl-2*H*-indazol-5-yl)-1*H*-imidazo[4,5-*c*]pyridine (S34)

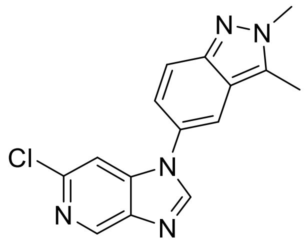

General procedure C from **S33** (0.390 g, 1.36 mmol), yield quant.; pale purple solid. ^{1}H NMR (400 MHz, DMSO-d_6) δ 8.89 (s, 1H), 8.73 (s, 1H), 8.03 (s, 1H), 7.73 (d, *J* = 8.8 Hz 1H), 7.63 (s, 1H), 7.44 (dd, *J* = 1.6, 8.8 Hz, 1H), 4.12 (s, 3H), 2.67 (s, 3H); ^{13}C NMR (100 MHz, DMSO-d_6) δ 146.8, 145.8, 143.0, 141.4, 140.7, 140.4, 133.3, 126.8, 122.4, 120.3, 118.4, 116.2, 105.9, 37.5, 9.4; *m/z* (ESI$^+$) 298.1 ([M+H]$^+$, 100%).

1-(2,3-Dimethyl-2*H*-indazol-5-yl)-6-(4-fluoro-2-methylphenyl)-1*H*-imidazo[4,5-*c*]pyridine (24)

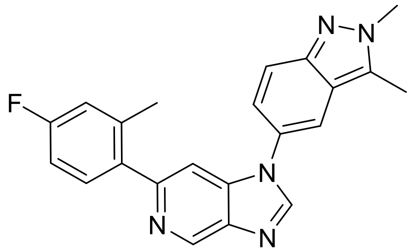

General procedure D from **S34** (150 mg, 0.504 mmol); yield 69% (129 mg, 0.347 mmol); off-white solid. ^{1}H NMR (400 MHz, DMSO-d_6) δ 9.12 (d, *J* = 0.8 Hz, 1H), 8.70 (s, 1H), 8.03 (d, *J* = 1.6 Hz, 1H), 7.73 (d, *J* = 8.8 Hz, 1H), 7.59 (d, *J* = 0.8 Hz, 1H), 7.50-7.45 (m, 2H), 7.13 (dd, *J* = 2.8, 10 Hz, 1H), 7.06 (dt, *J* = 2.8, 8.8 Hz ,1H), 4.11 (s, 3H), 2.66 (s, 3H), 2.34 (s, 3H); ^{13}C NMR (100 MHz, DMSO-d_6) δ 161.4 (d, J = 244 Hz), 151.6, 145.7, 145.6, 141.2, 139.4, 138.9, 138.5 (d, J = 8.0 Hz), 137.2 (d, J = 3 Hz), 133.1, 131.7 (d, J = 8 Hz), 127.2, 122.5, 120.3, 118.3, 116.6 (d, J = 21 Hz), 115.8, 112.2 (d, J = 21 Hz), 105.8, 37.4, 20.1, 9.4; *m/z* (ESI$^+$) 372.1 ([M+H]$^+$, 100%); HRMS (ESI$^+$) $C_{22}H_{19}N_5F^+$ ([M+H]$^+$) requires 372.1619; found 372.1620.

5-(6-Chloro-1*H*-imidazo[4,5-*c*]pyridin-1-yl)-1-methyl-1*H*-pyrazolo[4,3-*b*]pyridine (S35)

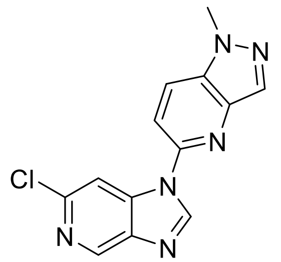

General procedure A, then general procedure B.1 and C; yield 38% over 3 steps (447 mg, 1.22 mmol); pale orange solid. ^{1}H NMR (500 MHz, $CDCl_3$) δ 8.95 (d, *J* = 0.9 Hz, 1H), 8.61 (s, 1H), 8.31 (d, *J* = 1.0 Hz, 1H), 8.17 (d, *J* = 0.9 Hz, 1H), 8.04 (dd, *J* = 8.9, 1.0 Hz, 1H), 7.57 (d, *J* = 8.9 Hz, 1H), 4.20 (s, 3H); ^{13}C NMR (126 MHz, $CDCl_3$) δ 145.4, 144.4, 143.4, 142.4, 141.0, 140.3, 139.4, 133.5, 131.6, 120.5, 112.5, 108.4, 36.5; *m/z* (ESI^+) 285.1 ($[M+H]^+$, 100%); HRMS (ESI^+) $C_{13}H_{10}ClN_6^+$ ($[M+H]^+$) requires 285.0650; found 285.0650.

5-(6-(4-Fluoro-2-methylphenyl)-1*H*-imidazo[4,5-*c*]pyridin-1-yl)-1-methyl-1*H*-pyrazolo[4,3-*b*]pyridine (25)

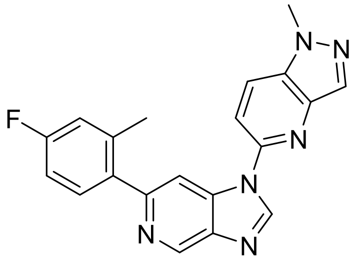

General procedure D from **S35** (50 mg, 0.18 mmol); yield 60% (38 mg, 0.11 mmol); pale yellow solid. ^{1}H NMR (400 MHz, $CDCl_3$) δ 9.23 (s, 1H), 8.64 (s, 1H), 8.25 (s, 1H), 8.12 (s, 1H), 8.01 (d, *J* = 8.8 Hz, 1H), 7.60 (d, *J* = 8.9 Hz, 1H), 7.44 – 7.40 (m, 1H), 7.02 – 6.94 (m, 2H), 4.17 (s, 3H), 2.37 (s, 3H); ^{13}C NMR (101 MHz, $CDCl_3$) δ 162.4 (d, *J* = 246 Hz), 153.7, 144.7, 142.8, 142.5, 140.3, 140.1, 138.7 (d, *J* = 8 Hz), 137.9, 137.1 (d, *J* = 3 Hz), 133.4, 131.6, 131.5 (d, *J* = 7 Hz), 120.3, 117.1 (d, *J* = 21 Hz), 112.9, 112.5 (d, *J* = 21 Hz), 108.2, 36.4, 20.5 (d, *J* = 2 Hz); *m/z* (ESI^+) 359.1 ($[M+H]^+$, 100%); HRMS (ESI^+) $C_{20}H_{16}FN_6^+$ ($[M+H]^+$) requires 359.1415; found 359.1413.

5-(6-Chloro-1*H*-imidazo[4,5-*c*]pyridin-1-yl)-1-methyl-1*H*-pyrazolo[3,4-*c*]pyridine (S36)

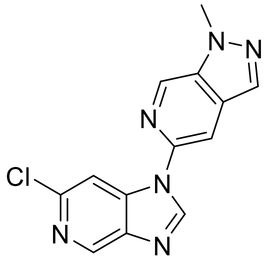

General procedure A, then general procedure B.1 and C; yield 27% over 3 steps (183 mg, 0.643 mmol); pale orange solid. ^{1}H NMR (500 MHz, $CDCl_3$) δ 8.98 (s, 1H), 8.95 (s, 1H), 8.57 (s, 1H), 8.19 (s, 1H), 7.96 (s, 1H), 7.81 (s, 1H), 4.30 (s, 3H); ^{13}C NMR (126 MHz, $CDCl_3$) δ 145.0, 144.0, 142.4, 141.0, 140.4, 139.7, 136.1, 133.2, 132.7, 129.7, 107.5, 105.6, 36.5; *m/z* (ESI$^+$) 285.0 ([M+H]$^+$, 100%); HRMS (ESI$^+$) $C_{13}H_{10}ClN_6^+$ ([M+H]$^+$) requires 285.0650; found 285.0650.

5-(6-(4-Fluoro-2-methylphenyl)-1*H*-imidazo[4,5-*c*]pyridin-1-yl)-1-methyl-1*H*-pyrazolo[3,4-*c*]pyridine (26)

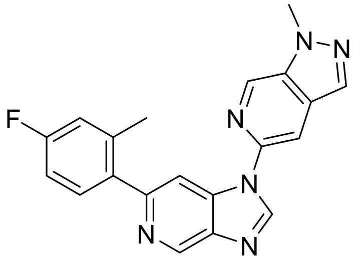

General procedure D from **S36** (50 mg, 0.18 mmol); yield 25% (16 mg, 0.045 mmol); pale yellow solid. ^{1}H NMR (400 MHz, $CDCl_3$) δ 9.25 (d, *J* = 1.1 Hz, 1H), 8.97 (t, *J* = 1.1 Hz, 1H), 8.63 (s, 1H), 8.17 (d, *J* = 0.9 Hz, 1H), 7.92 (d, *J* = 1.1 Hz, 1H), 7.84 (d, *J* = 1.2 Hz, 1H), 7.42 (dd, *J* = 8.4, 6.0 Hz, 1H), 7.03 – 6.93 (m, 2H), 4.28 (s, 3H), 2.38 (s, 3H); ^{13}C NMR (101 MHz, $CDCl_3$) δ 162.4 (d, *J* = 246 Hz), 153.4, 143.4, 142.6, 140.8, 140.2, 138.7 (d, *J* = 8 Hz), 138.2, 137.2 (d, *J* = 3 Hz), 136.0, 133.1, 132.6, 131.5 (d, *J* = 9 Hz), 129.7, 117.1 (d, *J* = 21 Hz), 112.5 (d, *J* = 21 Hz), 107.3, 105.7, 36.5, 20.6 (d, *J* = 2 Hz); *m/z* (ESI$^+$) 359.1 ([M+H]$^+$, 100%); HRMS (ESI$^+$) $C_{20}H_{16}FN_6^+$ ([M+H]$^+$) requires 359.1415; found 359.1415.

N-(2-Chloro-5-nitropyridin-4-yl)-1-methyl-1H-pyrazolo[3,4-b]pyridin-5-amine (S37)

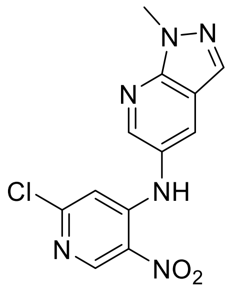

General procedure A; yield 84% (665 mg, 2.18 mmol); yellow solid. ^{1}H NMR (DMSO-d_6, 400 MHz) δ 10.03 (s, 1H), 8.98 (s, 1H), 8.53 (d, J = 2 Hz, 1H), 8.26 (d, J = 2 Hz, 1H), 8.20 (s, 1H), 6.70 (s, 1H), 4.10 (s, 3H); ^{13}C NMR (DMSO-d_6, 100 MHz) δ 155.2, 1450.1, 149.2, 149.1, 148.6, 132.7, 130.8, 129.0, 128.0, 115.6, 108.8, 34.3; m/z (ESI^{+}) 305.1 ([M+H]$^{+}$, 100%).

6-Chloro-N^4-(1-methyl-1H-pyrazolo[3,4-b]pyridin-5-yl)pyridine-3,4-diamine (S38)

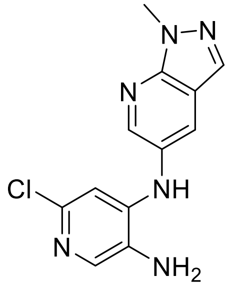

General procedure B.2 from **S37** (650 mg, 2.13 mmol); yield 61% (360 mg, 1.31 mmol); yellow solid. ^{1}H NMR (DMSO-d_6, 400 MHz) δ 8.45 (d, J = 2.4 Hz, 1H), 8.09 (s, 1H), 8.02 (d, J = 2.4 Hz, 1H), 7.82 (br s, 1H), 7.63 (s, 1H), 6.45 (s, 1H), 4.97 (br s, 2H), 4.07 (s, 3H); ^{13}C (DMSO-d_6, 100 MHz) δ 148.1, 146.6, 142.2, 139.5, 134.8, 132.8, 131.9, 131.1, 123.5, 115.6, 105.5, 34.2; m/z (ESI^{+}) 275.0 ([M+H]$^{+}$, 100%).

5-(6-Chloro-1H-imidazo[4,5-c]pyridin-1-yl)-1-methyl-1H-pyrazolo[3,4-b]pyridine (S39)

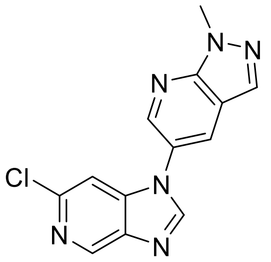

General procedure C from **S38** (340 mg, 1.24 mmol); yield 87% (307 mg, 1.08 mmol); beige solid. ^{1}H NMR (DMSO-d_6, 400 MHz) δ 8.90 (s, 1H), 8.87 (d, *J* = 2.4 Hz, 1H), 8.77 (s, 1H), 8.62 (d, *J* = 2 Hz, 1H), 8.30 (s,1H), 7.73 (s, 1H), 4.15 (s, 3H); ^{13}C NMR (DMSO-d_6, 100 MHz) δ 149.8, 147.5, 146.1, 143.9, 142.0, 141.9, 141.0, 133.1, 127.4, 125.9, 115.3, 106.4, 34.4; *m/z* (ESI^{+}) 285.1 ([M+H]$^{+}$, 100%).

6-(4-Fluoro-2-methylphenyl)-1-{1-methyl-1*H*-pyrazolo[3,4-*b*]pyridin-5-yl}-1*H*-imidazo[4,5-*c*]pyridine (OXS008203, 27)

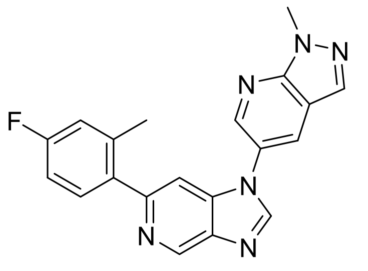

General procedure D from **S39** (150 mg, 0.527 mmol); yield 23% (44 mg, 0.12 mmol); beige solid. ^{1}H NMR (400 MHz, $CDCl_3$) δ 9.28 (d, *J* = 0.9 Hz, 1H), 8.71 (d, *J* = 2.4 Hz, 1H), 8.20 (s, 1H), 8.19 (d, *J* = 2.4 Hz, 1H), 8.15 (s, 1H), 7.40 (d, *J* = 0.9 Hz, 1H), 7.34 (dd, *J* = 8.4, 6.0 Hz, 1H), 6.98 (dd, *J* = 9.8, 2.6 Hz, 1H), 6.93 (td, *J* = 8.4, 2.6 Hz, 1H), 4.25 (s, 3H), 2.35 (s, 3H); ^{13}C NMR ($CDCl_3$, 100 MHz) δ 162.6 (d, J = 247 Hz), 153.8, 149.9, 145.3, 144.3, 143.0, 140.1, 139.8, 138.8 (d, J = 8 Hz), 136.9 (d, J = 3 Hz), 132.3, 131.6 (d, J = 8 Hz), 126.0, 125.9, 117.4 (d, J = 21 Hz), 115.4, 112.7 (d, J = 21 Hz), 105.3, 34.5, 20.7; *m/z* (ESI^{+}) 359.1 ([M+H]$^{+}$, 100%); HRMS (ESI^{+}) $C_{20}H_{16}N_6F^{+}$ ([M+H]$^{+}$) requires 359.1415; found 359.1415.

6-(6-Fluoro-4-methyl-3-pyridyl)-1-(2-methylindazol-6-yl)imidazo[4,5-*c*]pyridine (28)

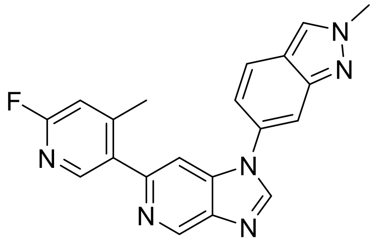

General procedure D from **S31** (75 mg, 0.26 mmol); yield 88% (83 mg, 0.23 mmol); pale yellow solid. ^{1}H NMR (400 MHz, $CDCl_3$) δ 9.26 (s, 1H), 8.29 (s, 1H), 8.17 (s, 1H), 8.05 (s, 1H), 7.87 (d, *J* = 8.8 Hz, 1H), 7.80 (s, 1H), 7.58 (d, *J* = 0.9 Hz, 1H), 7.20 (dd, *J* = 8.8, 1.8 Hz, 1H), 6.83 (s, 1H), 4.27 (s, 3H), 2.42 (s, 3H); ^{13}C NMR (101 MHz, $CDCl_3$) δ 163.5 (d, *J* = 239 Hz), 152.0 (d, *J* = 8 Hz), 149.8, 148.5, 147.8 (d, *J* = 16 Hz), 144.6, 143.1, 140.4, 139.5, 135.3 (d, *J* = 4 Hz), 133.0, 124.7, 123.0, 121.8, 118.4, 112.6, 110.5 (d, *J* = 37 Hz), 106.5, 40.9, 20.4; *m/z* (ESI^{+}) 359.1 ([M+H]$^{+}$, 100%); HRMS (ESI^{+}) $C_{20}H_{16}FN_6^{+}$ ([M+H]$^{+}$) requires 359.1415; found 359.1409.

5-(6-(6-Fluoro-4-methylpyridin-3-yl)-1*H*-imidazo[4,5-*c*]pyridin-1-yl)-1-methyl-1*H*-pyrazolo[4,3-*b*]pyridine (29)

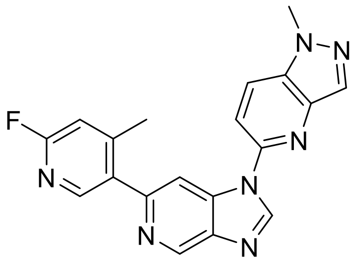

General procedure D from **S35** (50 mg, 0.18 mmol); yield 25% (16 mg, 0.045 mmol); pale orange solid. ^{1}H NMR (500 MHz, $CDCl_3$) δ 9.28 (s, 1H), 8.67 (s, 1H), 8.28 (s, 3H), 8.21 (s, 1H), 8.04 (d, *J* = 8.9 Hz, 1H), 7.62 (d, *J* = 8.8 Hz, 1H), 6.90 (s, 1H), 4.19 (s, 3H), 2.47 (s, 3H); ^{13}C NMR (126 MHz, $CDCl_3$) δ 163.6 (d, *J* = 239 Hz), 152.2 (d, *J* = 8 Hz), 150.6, 148.0 (d, *J* = 16 Hz), 144.8, 143.2, 143.1, 140.7, 140.5, 138.2, 135.4 (d, *J* = 4 Hz), 133.6, 131.8, 120.6, 113.0, 110.7 (d, *J* = 37 Hz), 108.9, 36.6, 20.6 (d, *J* = 3 Hz); *m/z* (ESI$^+$) 360.1 ([M+H]$^+$, 100%); HRMS (ESI$^+$) $C_{19}H_{15}FN_7^+$ ([M+H]$^+$) requires 360.1367; found 360.1368.

5-(6-(6-Fluoro-4-methylpyridin-3-yl)-1*H*-imidazo[4,5-*c*]pyridin-1-yl)-1-methyl-1*H*-pyrazolo[3,4-*b*]pyridine (30)

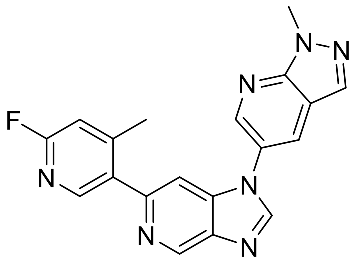

General procedure D from **S39** (40 mg, 0.14 mmol); yield 18% (9 mg, 0.03 mmol); white solid. ^{1}H NMR (500 MHz, $CDCl_3$) δ 9.34 (s, 1H), 8.74 (d, *J* = 2.4 Hz, 1H), 8.30 – 8.16 (m, 4H), 7.47 (s, 1H), 6.89 (s, 1H), 4.28 (s, 3H), 2.46 (s, 3H); ^{13}C NMR (125 MHz, $CDCl_3$) δ 163.6 (d, *J* = 240 Hz), 152.1 (d, *J* = 8 Hz), 150.4, 149.9, 147.8 (d, *J* = 16 Hz), 145.2, 144.6, 143.4, 140.2, 140.1, 135.0 (d, *J* = 4 Hz), 132.4, 126.2, 125.6, 115.4, 110.7 (d, *J* = 37 Hz), 105.7, 34.6, 20.5 (d, *J* = 3 Hz); *m/z* (ESI$^+$) 360.1 ([M+H]$^+$, 80%); HRMS (ESI$^+$) $C_{19}H_{15}N_7F^+$ ([M+H]$^+$) requires 360.13675; found 360.13701.

5-(6-(6-Fluoro-4-methylpyridin-3-yl)-1*H*-imidazo[4,5-*c*]pyridin-1-yl)-1-methyl-1*H*-pyrazolo[3,4-*c*]pyridine (31)

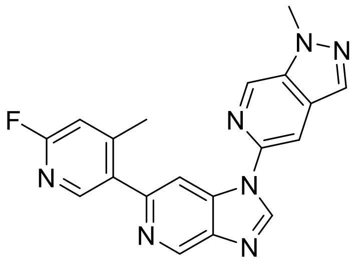

General procedure D from **S36** (50 mg, 0.18 mmol); yield 28% (18 mg, 0.050 mmol); pale orange solid. ^{1}H NMR (600 MHz, $CDCl_3$) δ 9.28 (d, *J* = 1.1 Hz, 1H), 8.98 (s, 1H), 8.63 (s, 1H), 8.25 (s, 1H), 8.18 (s, 1H), 8.00 (d, *J* = 0.9 Hz, 2H), 7.85 (d, *J* = 1.1 Hz, 1H), 6.89 (s, 1H), 2.47 (s, 3H), 1.59 (s, 3H); ^{13}C NMR (151 MHz, $CDCl_3$) δ 163.6 (d, *J* = 239 Hz), 152.2 (d, *J* = 8 Hz), 150.2, 147.9 (d, *J* = 16 Hz), 143.7, 143.1, 140.9, 140.8, 138.5, 136.2, 135.5 (d, *J* = 4 Hz), 133.4, 132.9, 129.9, 110.6 (d, *J* = 37 Hz), 108.0, 106.0, 36.7, 20.6 (d, *J* = 3 Hz); *m/z* (ESI^+) 360.1 ($[M+H]^+$, 100%); HRMS (ESI^+) $C_{19}H_{15}FN_7^+$ ($[M+H]^+$) requires 360.1367; found 360.1369.

2-Methyl-2*H*-pyrazolo[3,4-*c*]pyridin-5-amine (S40)

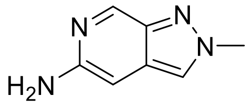

$Pd_2(dba)_3$ (87 mg, 95 µmol) was added to a stirred solution of 5-bromo-2-methyl-2*H*-pyrazolo[3,4-*c*]pyridine (405 mg, 1.91 mmol), benzophenone imine (390 µL, 2.23 mmol), sodium *tert*-butoxide (275 mg, 2.86 mmol), and rac-BINAP (119 mg, 0.191 mmol) in toluene (19 mL) at room temperature. The resultant mixture was heated to 80 °C for 3 h, then cooled to room temperature, filtered through Celite® (EtOAc) and concentrated. To a solution of the resulting residue in THF (10 mL) was added dropwise 1 M aqueous HCl (10 mL) at room temperature. The resultant mixture was stirred for 1 h, then basified to pH 10 with 1 M aqueous NaOH and concentrated. Purification via flash column chromatography (5% MeOH in DCM) gave product **S40** (194 mg, 1.32 mmol, 69%) as a pale orange solid. ^{1}H NMR (400 MHz, $CDCl_3$) δ 8.90 (s, 1H), 7.63 (s, 1H), 6.54 (s, 1H), 4.18 (s, 3H); ^{13}C NMR (101 MHz, $CDCl_3$) δ 150.7, 143.0, 142.5, 127.3, 120.8, 91.0, 40.6; HRMS (ESI^+) $C_7H_9N_4^+$ ($[M+H]^+$) requires 149.0822; found 149.0822.

5-(6-Chloro-1*H*-imidazo[4,5-*c*]pyridin-1-yl)-2-methyl-2*H*-pyrazolo[3,4-*c*]pyridine (S41)

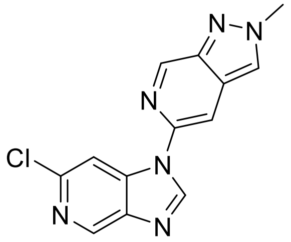

General procedure A from **S40** (205 mg, 1.38 mmol), then general procedure B.1 and C; yield 24% (125 mg, 0.33 mmol) over 3 steps; pale orange solid. ^{1}H NMR (500 MHz, $CDCl_3$) δ 9.28 (t, *J* = 1.1 Hz, 1H), 8.95 (d, *J* = 0.9 Hz, 1H), 8.59 (s, 1H), 8.15 (s, 1H), 7.95 (d, *J* = 0.9 Hz, 1H), 7.72 (d, *J* = 1.3 Hz, 1H), 4.40 (s, 3H); ^{13}C NMR (126 MHz, $CDCl_3$) δ 145.0, 144.9, 144.3, 144.3, 142.3, 141.1, 140.1, 139.7, 125.6, 124.4, 107.5, 104.3, 41.4; *m/z* (ESI$^+$) 285.1 ([M+H]$^+$, 100%); HRMS (ESI$^+$) $C_{13}H_{10}ClN_6^+$ ([M+H]$^+$) requires 285.0650; found 285.0651.

5-(6-(6-Fluoro-4-methylpyridin-3-yl)-1*H*-imidazo[4,5-*c*]pyridin-1-yl)-2-methyl-2*H*-pyrazolo[3,4-*c*]pyridine (32)

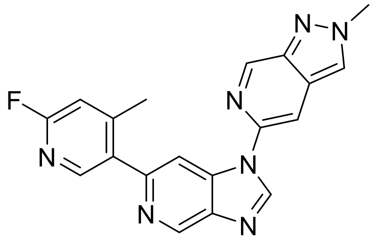

General procedure D from **S41** (50 mg, 0.18 mmol); yield 43% (27 mg, 75 mmol); pale brown solid. ^{1}H NMR (500 MHz, $CDCl_3$) δ 9.28 – 9.26 (m, 1H), 8.64 (s, 0H), 8.25 (s, 0H), 8.12 (s, 0H), 7.98 (d, *J* = 1.0 Hz, 1H), 7.74 (d, *J* = 1.3 Hz, 1H), 6.88 (s, 1H), 4.37 (s, 3H), 2.46 (s, 3H); ^{13}C NMR (126 MHz, $CDCl_3$) δ 163.6 (d, *J* = 239 Hz), 152.3 (d, *J* = 8 Hz), 150.0, 147.9 (d, *J* = 15 Hz), 145.1, 144.4, 144.0, 143.1, 140.8, 140.5, 138.5, 135.5 (d, *J* = 4 Hz), 125.8, 124.5, 110.6 (d, *J* = 36 Hz), 108.1, 104.6, 41.5, 20.6 (d, *J* = 2 Hz); *m/z* (ESI$^+$) 360.1 ([M+H]$^+$, 100%); HRMS (ESI$^+$) $C_{19}H_{15}FN_7^+$ ([M+H]$^+$) requires 360.1367; found 360.1367.

tert-Butyl 6-(6-chloro-1H-imidazo[4,5-c]pyridin-1-yl)-3-methyl-1H-indazole-1-carboxylate (S42)

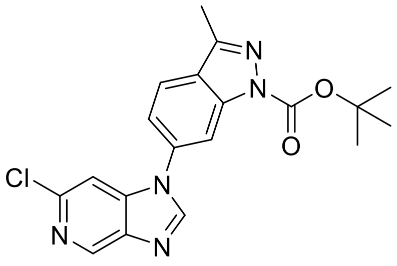

S23 (300 mg, 0.780 mmol) was dissolved in ethanol (15 mL), and aqueous HCl (2 M, 1.3 mL, 2.6 mmol) was added. The mixture was stirred at room temperature for 2 h, and then it was concentrated, diluted with EtOAc, washed with 1x$NaHCO_3$ and 1xbrine, dried over anhydrous Na_2SO_4 and concentrated. To a suspension of the resulting crude product in anhydrous DCM (3.9 mL) were added triethylamine (120 μL, 0.860 mmol), Boc anhydride (255 mg, 1.17 mmol), and DMAP (10 mg, 0.080 mmol). The mixture was stirred at room temperature for 3 h, then it was diluted with brine, extracted with 3xDCM, dried over anhydrous Na_2SO_4, and concentrated to give Boc-protected indazole **S42** (300 mg, 0.780 mmol, quant.) as an orange solid. ^{1}H NMR (400 MHz, $CDCl_3$) δ 8.96 (d, *J* = 1.0 Hz, 1H), 8.30 (s, 1H), 8.25 (s, 1H), 7.88 (dd, *J* = 8.3, 0.7 Hz, 1H), 7.55 (d, *J* = 0.9 Hz, 1H), 7.43 (dd, *J* = 8.4, 1.9 Hz, 1H), 2.67 (s, 3H), 1.72 (s, 9H); ^{13}C NMR (100 MHz, $CDCl_3$) δ 149.0, 148.5, 145.2, 144.9, 142.9, 142.7, 140.9, 140.8, 135.3, 126.0, 122.6, 119.2, 110.4, 105.8, 85.8, 28.3, 12.5; *m/z* (ESI$^+$) 384.1 ([M+H]$^+$, 100%); HRMS (ESI$^+$) $C_{19}H_{19}N_5O_2Cl^+$ ([M+H]$^+$) requires 384.1222; found 384.1220.

6-(6-Fluoro-4-methylpyridin-3-yl)-1-(3-methyl-1H-indazol-6-yl)-1H-imidazo[4,5-c]pyridine (33)

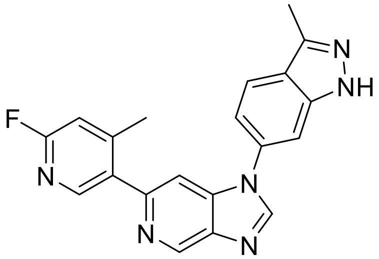

General procedure D from **S42** (237 mg, 0.618 mmol); yield 7% (16 mg, 0.045 mmol); white solid. ^{1}H NMR (500 MHz, DMSO-d_6) δ 12.94 (s, 1H), 9.19 (d, *J* = 1.0 Hz, 1H), 8.87 (s, 1H), 8.28 (s, 1H), 7.95 (d, *J* = 8.5 Hz, 1H), 7.84 (d, *J* = 1.1 Hz, 1H), 7.82 (d, *J* = 1.8 Hz, 1H), 7.45 (dd, *J* = 8.5, 1.8 Hz, 1H), 7.17 (s, 1H), 2.55 (s, 3H), 2.42 (s, 3H); ^{13}C NMR (126 MHz, DMSO-d_6) δ 162.6 (d, *J* = 236 Hz), 152.0 (d, *J* = 8.6 Hz), 148.6, 147.6 (d, *J* = 16 Hz), 146.1, 141.8, 141.6, 140.8, 140.0, 138.8, 135.2 (d, *J* = 4 Hz), 133.0, 121.8, 121.7, 116.1, 110.2 (d, *J* = 37 Hz), 106.9, 105.5, 20.0 (d,

J = 3 Hz), 11.7; *m/z* (ESI$^+$) 359.1 ([M+H]$^+$, 100%); HRMS (ESI$^+$) $C_{20}H_{16}N_6F^+$ ([M+H]$^+$) requires 359.1415; found 359.1414.

6-(6-Chloro-1*H*-imidazo[4,5-*c*]pyridin-1-yl)-1-(4-methoxybenzyl)-3-methyl-3*a*,7*a*-dihydro-1*H*-pyrazolo[4,3-*b*]pyridine (S43)

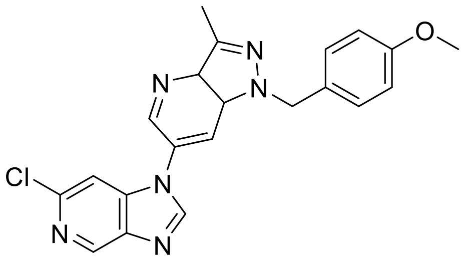

Step 1: 4-Methoxybenzoyl chloride (0.70 mL, 5.66 mmol) was added dropwise to a stirred solution of 6-bromo-3-methyl-3*a*,7*a*-dihydro-1*H*-pyrazolo[4,3-*b*]pyridine (1.00 g, 4.72 mmol) and K_2CO_3 (1.30 g, 9.43 mmol) in DMF (24 mL) at room temperature. The resultant mixture was stirred for 16 h then concentrated. EtOAc and brine were added, and the aqueous layer was extracted with 3xEtOAc, dried over $MgSO_4$, and concentrated.

Step 2: $Pd_2(dba)_3$ (133 mg, 0.145 mmol) was added to a stirred solution of the residue from the previous step, benzophenone imine (590 µL, 3.37 mmol), sodium *tert*-butoxide (419 mg, 4.36 mmol) and rac-BINAP (181 mg, 0.291 mmol) in toluene (47 mL) at room temperature. The resultant mixture was heated to 80 °C for 3 h, then cooled to room temperature, filtered through Celite® (EtOAc), and concentrated.

Step 3: 1 M aqueous HCl (25 mL) was added dropwise to a stirred solution of the residue from the previous step in THF (25 mL) at room temperature. The resultant mixture was stirred for 1 h, basified to pH 10 with 1 M aqueous NaOH, and concentrated.

Step 4: Triethylamine (1.32 mL, 9.44 mmol) was added dropwise to a stirred solution of 2,4-dichloro-5-nitro-pyridine (911 mg, 4.72 mmol) and the residue from the previous step in acetonitrile (9.0 mL) at room temperature. The resultant mixture was heated to 80 °C for 16 h, cooled to room temperature, and concentrated *in vacuo*.

Step 5: Iron powder (1.36 g, 24.3 mmol) was added to stirred acetic acid (15 mL) at room temperature. The resultant mixture was heated to 80 °C for 20 min before the addition of a solution of the residue from the previous step in acetic acid (15 mL) was added. The resultant mixture was stirred at 80 °C for 30 min, cooled to room temperature, filtered through Celite® (DCM), and concentrated. DCM and a saturated aqueous solution of $NaHCO_3$ were added. The aqueous layer was then extracted with 3xDCM, dried over $MgSO_4$, and concentrated.

Step 6: Formic acid (180 µL, 4.72 mmol) was added dropwise to a stirred solution of the residue from the previous step and triethylorthoformate (15.7 mL, 94.4 mmol) at room temperature. The resultant mixture was stirred at 100 °C for 24 h, cooled to room temperature, and concentrated. EtOAc (20 mL) and saturated aqueous $NaHCO_3$ were added, and the aqueous layer was extracted with EtOAc (3 × 20 mL), dried over $MgSO_4$ and concentrated. Purification via flash column chromatography (4% MeOH in DCM) gave the desired product **S43** as a pale brown solid (681 mg, 1.68 mmol, 36%). ^1H NMR (400 MHz, $CDCl_3$) δ 8.95 (s, 1H), 8.61 (d, *J* = 2.2 Hz, 1H), 8.15 (s, 1H), 7.53 (d, *J* = 2.2 Hz, 1H), 7.22 (d, *J* = 8.7 Hz, 1H), 6.89 (d, *J* = 8.7 Hz, 2H), 5.54 (s, 2H), 3.78 (s, 3H), 2.75 (s, 3H); ^{13}C NMR (101 MHz, $CDCl_3$) δ 159.8, 145.2, 144.5, 143.4, 142.8, 141.4, 140.8, 140.5, 139.8, 132.0, 128.9, 128.8, 128.4, 127.2, 114.5, 112.4, 105.3, 55.3, 53.8; HRMS (ESI^+) $C_{21}H_{20}ClN_6O^+$ ($[M+H]^+$) requires 405.1225; found 405.1227.

6-(6-(6-Fluoro-4-methylpyridin-3-yl)-1*H*-imidazo[4,5-*c*]pyridin-1-yl)-3-methyl-1*H*-pyrazolo[4,3-*b*]pyridine (34)

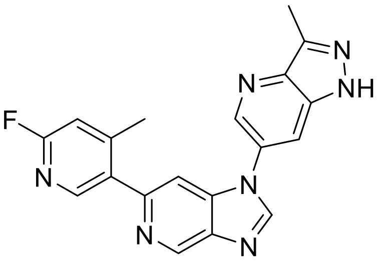

General procedure D from **S43** (300 mg, 0.741 mmol). The resulting residue was dissolved in TFA (6 mL), and the mixture was heated to 75 °C for 6 h. Then, it was cooled to room temperature before the addition of 1 M aqueous NaOH until pH >7. The aqueous layer was extracted with 3xDCM, dried over anhydrous $MgSO_4$, and concentrated. Purification via flash column chromatography (6% MeOH in DCM) gave the desired product **34** (94 mg, 0.26 mmol, 35%) as a pale yellow solid. ^1H NMR (600 MHz, DMSO-d_6) δ 9.20 (d, *J* = 0.9 Hz, 1H), 8.89 (s, 1H), 8.83 (d, *J* = 2.2 Hz, 1H), 8.36 (d, *J* = 2.1 Hz, 1H), 8.28 (s, 1H), 7.93 (d, *J* = 0.9 Hz, 1H), 7.17 (s, 1H), 2.60 (s, 3H), 2.43 (s, 3H); ^{13}C NMR (151 MHz, DMSO-d_6) δ 162.6 (d, *J* = 236 Hz), 152.0 (d, *J* = 8 Hz), 148.8, 147.7 (d, *J* = 16 Hz), 146.2, 142.0, 141.8, 140.6, 139.9, 139.1, 139.0, 135.1 (d, *J* = 4 Hz), 132.7, 129.0, 114.2, 110.2 (d, *J* = 37 Hz), 106.9, 20.0, 10.7; HRMS (ESI^+) $C_{19}H_{15}FN_7^+$ ($[M+H]^+$) requires 360.1367; found 360.1367.

(*E*)-1-(4-Bromo-2-hydroxyphenyl)ethanone oxime (S44)

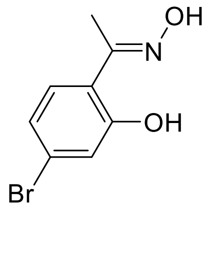

To a solution of 1-(4-bromo-2-hydroxy-phenyl)ethanone (4.00 g, 18.6 mmol) in EtOH (30 mL) were added $NH_2OH \cdot HCl$ (6.46 g, 93.0 mmol) and pyridine (19.5 mL, 242 mmol), and the mixture was stirred at 90 °C for 4 h. The mixture was quenched by HCl (2 M, 10 mL) and extracted with 3xEtOAc. The combined organic layers were washed with 3xwater, dried over anhydrous Na_2SO_4, filtered, and concentrated under reduced pressure. The residue was purified by flash column chromatography (50% EtOAc in petroleum ether) to afford oxime **S44** (3.80 g, 16.5 mmol, 89%) as a white solid. ^1H NMR (400 MHz, DMSO-d_6) δ 11.84 (br s, 1H), 11.65 (br s, 1H), 7.41 (d, *J*=8.16 Hz, 1H), 7.05 (br d, *J*=1.98 Hz, 1H), 2.23 (s, 3H); *m/z* (ESI^+) 230.9 ($[M+H]^+$, 100%).

(*E*)-1-(4-Bromo-2-hydroxyphenyl)ethanone *O*-acetyl oxime (S45)

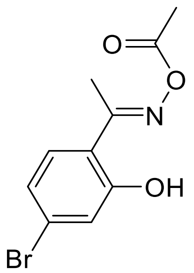

A solution of **S44** (3.80 g, 16.5 mmol) in acetyl acetate (7.7 mL, 83 mmol) was stirred at 25 °C for 1 h. The reaction mixture was extracted with 3xEtOAc. The combined organic layers were washed with 3xwater, dried over Na_2SO_4, filtered, and concentrated under reduced pressure to give the *O*-acetyl oxime **S45** (3.50 g, 12.9 mmol, 78%) as a yellow solid; *m/z* (ESI^+) 211.9 ($[M-AcO]^+$, 15%).

6-Bromo-3-methylbenzo[*d*]isoxazole (S46)

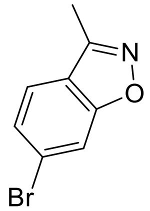

A solution of **S45** (3.50 g, 12.9 mmol) in pyridine (140 mL) was stirred at 130 °C for 12 hr. The reaction was quenched by HCl (2 M, 10 mL).The reaction mixture was extracted with 3xEtOAc. The combined organic layers were washed with 3xwater, dried over anhydrous Na_2SO_4, filtered and concentrated under reduced pressure. The residue was purified by flash column chromatography (10% EtOAc in petroleum ether) to afford 1,2-benzoxazole **S46** (1.50 g, 7.07 mmol, 55%) as a yellow solid. ^1H NMR (400 MHz, DMSO-d$_6$) δ 8.05 (d, *J*=1.47 Hz, 1H), 7.82 (d, *J*=7.82 Hz, 1H), 7.56 (dd, *J*=1.47, 8.31 Hz, 1H), 2.55 (s, 3H); *m/z* (ESI^+) 211.9 ($[M+H]^+$, 100%).

tert-Butyl (3-methylbenzo[d]isoxazol-6-yl)carbamate (S47)

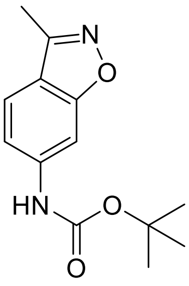

To a solution of **S46** (1.50 g, 7.07 mmol) and *tert*-butyl carbamate (1.24 g, 10.6 mmol) in dioxane (30 mL) were added Cs_2CO_3 (6.91 g, 21.2 mmol), $Pd(OAc)_2$ (318 mg, 1.41 mmol), and Xantphos (819 mg, 1.41 mmol), and the mixture was stirred at 110 °C for 12 h in a sealed tube. The reaction mixture was extracted with 3xEtOAc. The combined organic layers were washed with 3xwater, dried over Na_2SO_4, filtered and concentrated under reduced pressure to afford carbamate **S47** (600 mg, 2.42 mmol, 34%) as a yellow solid. *m/z* (ESI^+) 249.0 ($[M+H]^+$, 100%).

3-Methylbenzo[d]isoxazol-6-amine (S48)

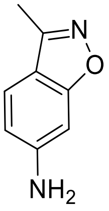

A solution of the Boc-protected compound **S47** (450 mg, 1.81 mmol) in HCl/EtOAc (4 M, 27 mL) was stirred at 25 °C for 2 h. The reaction mixture was extracted with 3xEtOAc. The combined organic layers were washed with 3xwater, dried over Na_2SO_4, filtered and concentrated under reduced pressure. The residue was purified by flash column chromatography (50% EtOAc in petroleum ether) to afford amine **S48** (200 mg, 1.35 mmol, 74%) as a yellow solid. *m/z* (ESI^+) 149.1 ($[M+H]^+$, 100%).

***N*-(2-Chloro-5-nitropyridin-4-yl)-3-methylbenzo[d]isoxazol-6-amine (S49)**

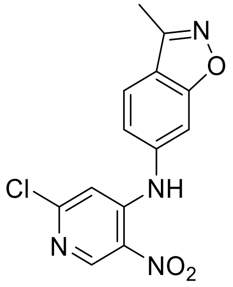

To a solution of amine **S48** (200 mg, 1.35 mmol) 2,4-dichloro-5-nitro-pyridine (261 mg, 1.35 mmol) in EtOH (3 mL) was added sodium acetate (332 mg, 4.05 mmol) and stirred at 80 °C for 12 h. The reaction mixture was extracted with 3xEtOAc. The combined organic layers were washed with 3xwater, dried over anhydrous Na_2SO_4, filtered, and concentrated under reduced pressure. The residue was purified by flash column chromatography (50% EtOAc in petroleum ether) to afford **S49** (300 mg, 0.985 mmol, 73%) as a yellow solid.

6-Chloro-*N*⁴-(3-methylbenzo[*d*]isoxazol-6-yl)pyridine-3,4-diamine (S50)

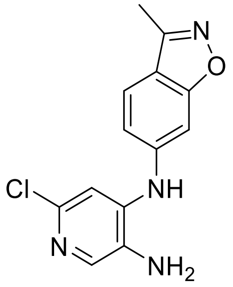

To a solution of nitro derivative **S49** (400 mg, 1.31 mmol) in THF (5 mL) was added $SnCl_2 \cdot 2H_2O$ (1.48 g, 6.56 mmol) and stirred at 60 °C for 3 h. The reaction mixture was extracted with 3xEtOAc. The combined organic layers were washed with 3xwater, dried over anhydrous Na_2SO_4, filtered, and concentrated under reduced pressure to give a residue. The residue was purified by flash column chromatography (50% EtoAc in petroleum ether) to afford amine **S50** (250 mg, 0.910 mmol, 69%) as a yellow solid. *m/z* (ESI^+) 275.0 ($[M+H]^+$, 100%).

6-(6-Chloro-1*H*-imidazo[4,5-*c*]pyridin-1-yl)-3-methylbenzo[*d*]isoxazole (S51)

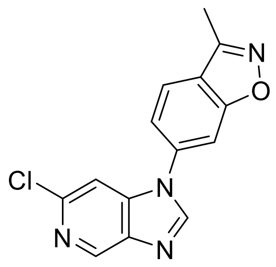

General procedure C from **S50** (150 mg, 0.546 mmol); yield 77% (120 mg, 0.422 mmol); yellow solid.

6-(6-(6-Fluoro-4-methylpyridin-3-yl)-1*H*-imidazo[4,5-*c*]pyridin-1-yl)-3-methylbenzo[*d*]isoxazole (35)

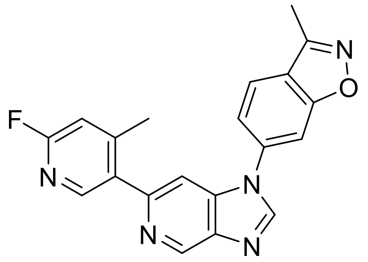

General procedure D from **S51** (50 mg, 0.18 mmol); yield 92% (59 mg, 0.16 mmol); white solid. ^{1}H NMR (600 MHz, $CDCl_3$) δ 9.31 (d, *J* = 0.8 Hz, 1H), 8.31 (s, 1H), 8.20 (s, 1H), 7.89 (d, *J* = 8.3 Hz, 1H), 7.73 (d, *J* = 1.4 Hz, 1H), 7.59 (d, *J* = 0.9 Hz, 1H), 7.51 (dd, *J* = 8.3, 1.7 Hz, 1H), 6.88 (s, 1H), 2.67 (s, 3H), 2.45 (s, 3H); ^{13}C NMR (151 MHz, $CDCl_3$) δ 163.6 (d, *J* = 240 Hz), 163.4, 155.3, 152.1 (d, *J* = 8 Hz), 150.5, 147.9 (d, *J* = 16 Hz), 144.2, 143.5, 140.4, 139.3, 136.8, 135.1 (d, *J* = 4 Hz), 123.5, 122.8, 119.6, 110.7 (d, *J* = 37 Hz), 106.1, 105.7, 20.5, 10.2; *m/z* (ESI$^+$) 360.2 ([M+H]$^+$, 100%); HRMS (ESI$^+$) $C_{20}H_{15}N_5OF^+$ ([M+H]$^+$) requires 360.1255; found 360.1258.

Supplementary Figures

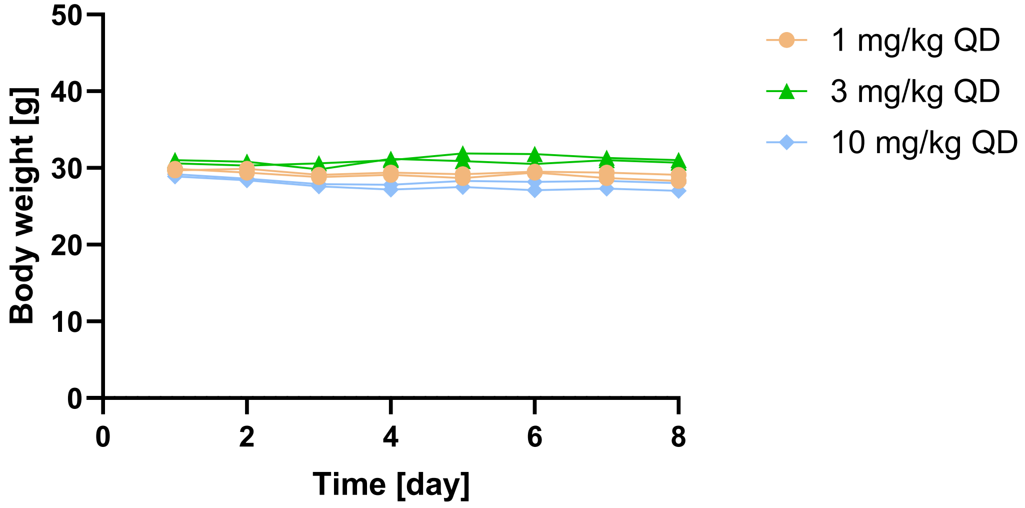

Figure S1. Tolerance study of OXS008203. No adverse effects were observed in any of the doses and no abnormalities were found during the necropsy.

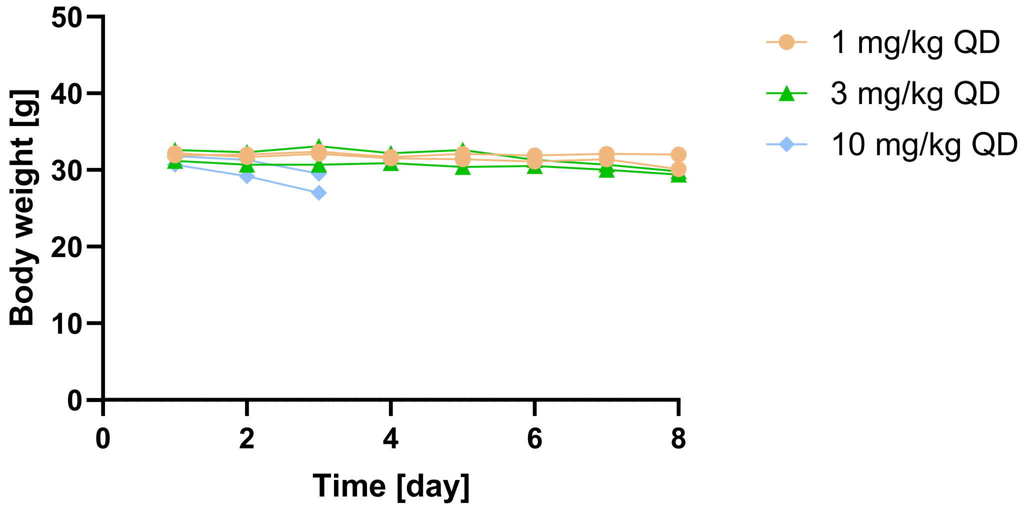

Figure S2. Tolerance study of OXS008255. At 1 mg/kg, no adverse effects were observed and no abnormalities were found during the necropsy. At 3 mg/kg, no adverse effects were observed but necropsy showed atrophied thymuses. At 10 mg/kg, animals were found dead at day 4.

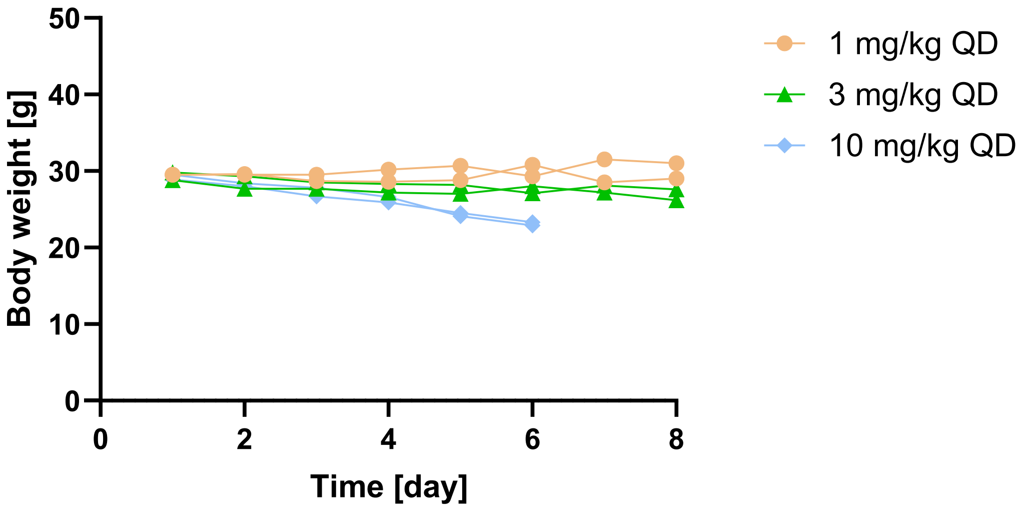

Figure S3. Tolerance study of OXS008474. At 1 and 3 mg/kg, no adverse effects were observed and no abnormalities were found during the necropsy. At 10 mg/kg, on day 6 the body weight decreased above 20% and animals were sent to euthanasia.

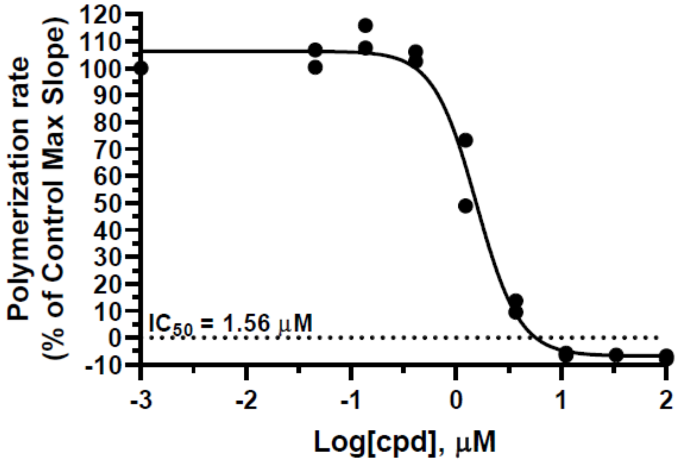

Figure S4. Inhibition of tubulin polymerisation by OXS008474 in a cell-free assay. Two replicates, representative curve of 3 biological repeats, EC_{50} value = mean +/- SEM.

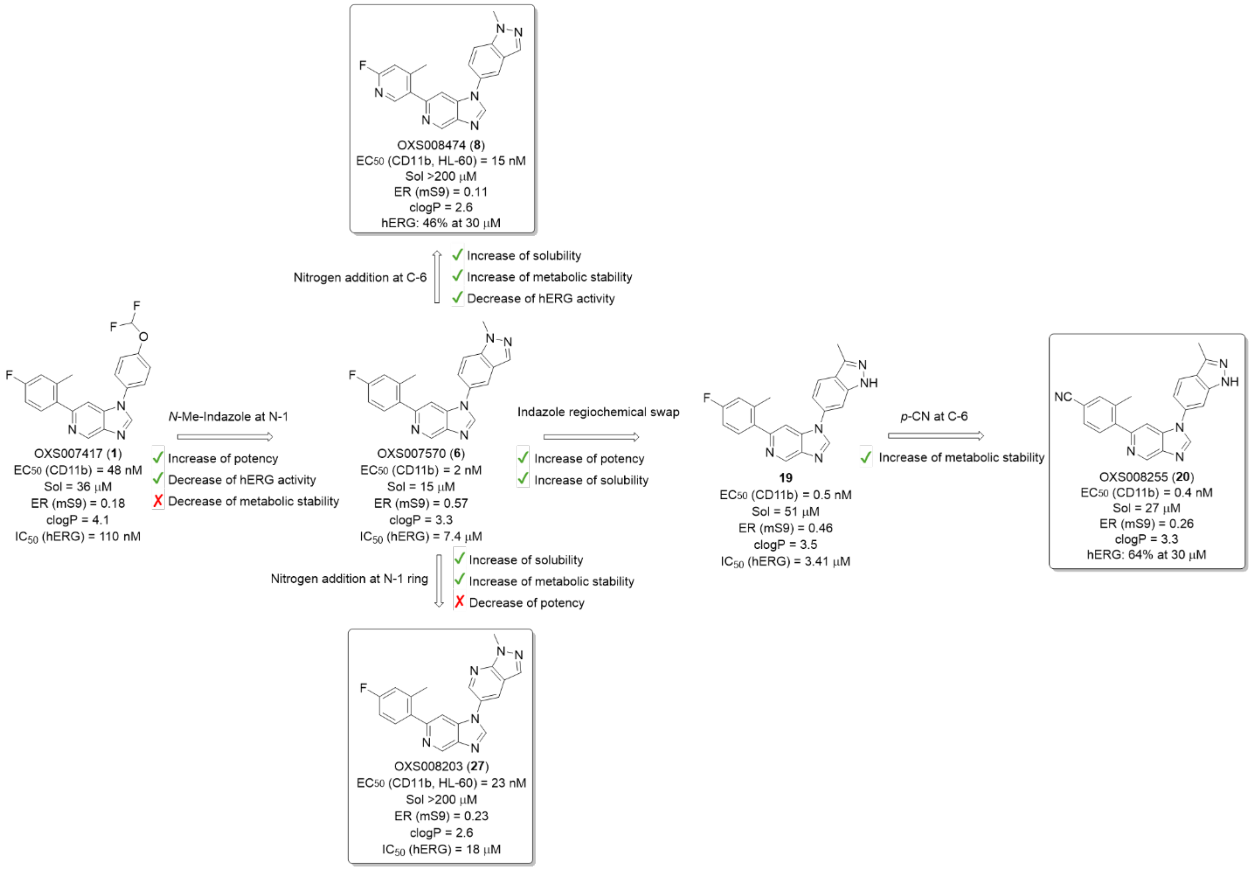

Figure S5. Roadmap from OXS007417 towards the three *in vivo* candidates.

NMR spectra of final compounds

3-Methyl-4-(1-(1-methyl-1*H*-indazol-5-yl)-1*H*-imidazo[4,5-*c*]pyridin-6-yl)benzonitrile (7)

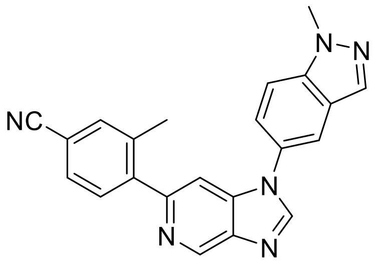

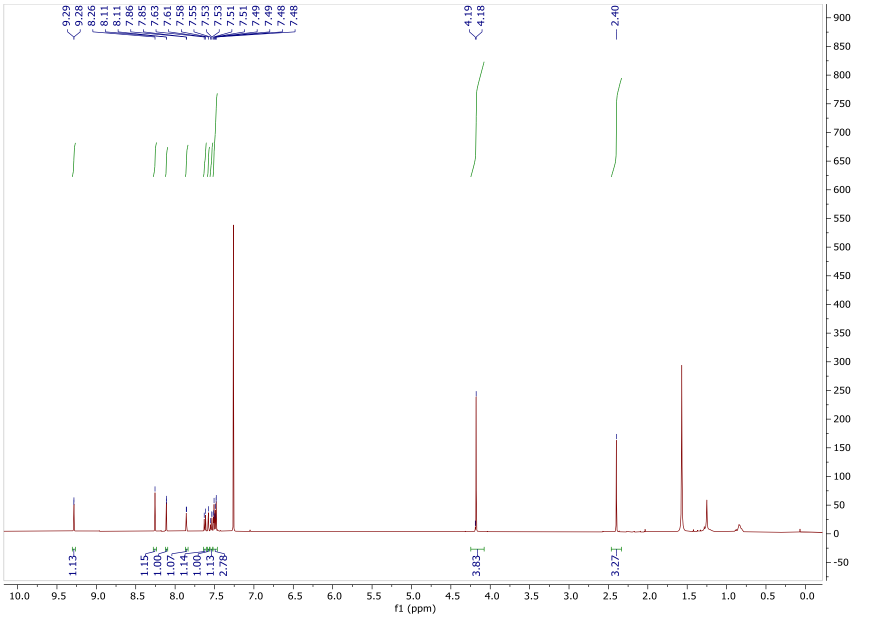

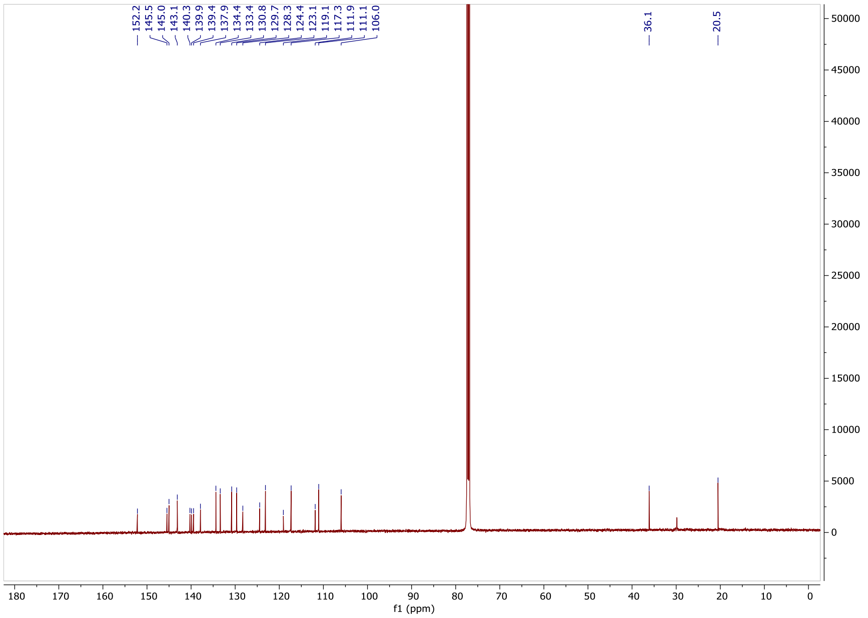

6-(6-Fluoro-4-methylpyridin-3-yl)-1-(1-methyl-1*H*-indazol-5-yl)-1*H*-imidazo[4,5-*c*]pyridine (OXS008474, 8)

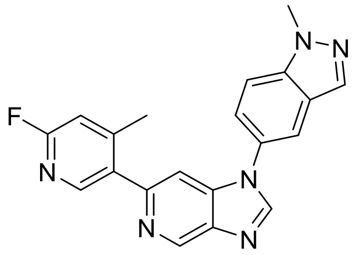

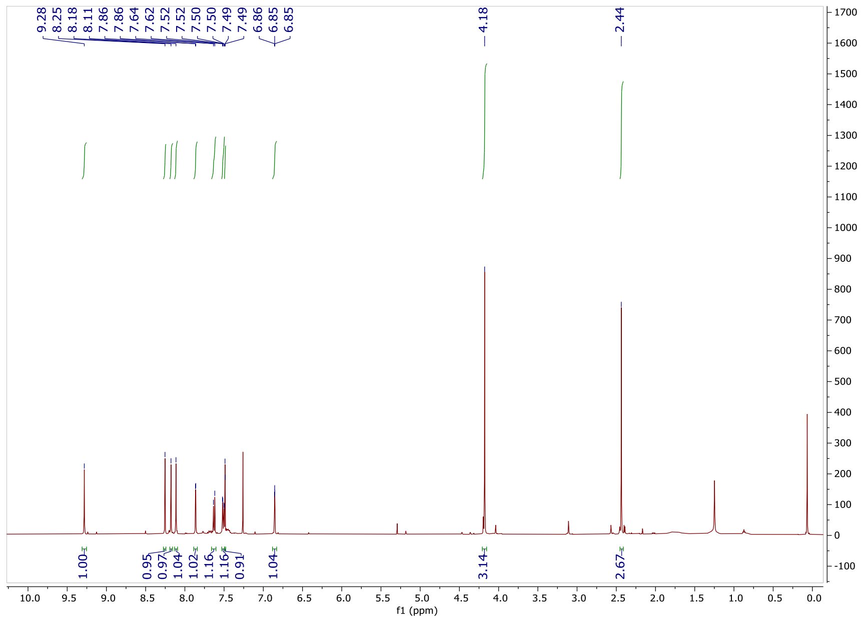

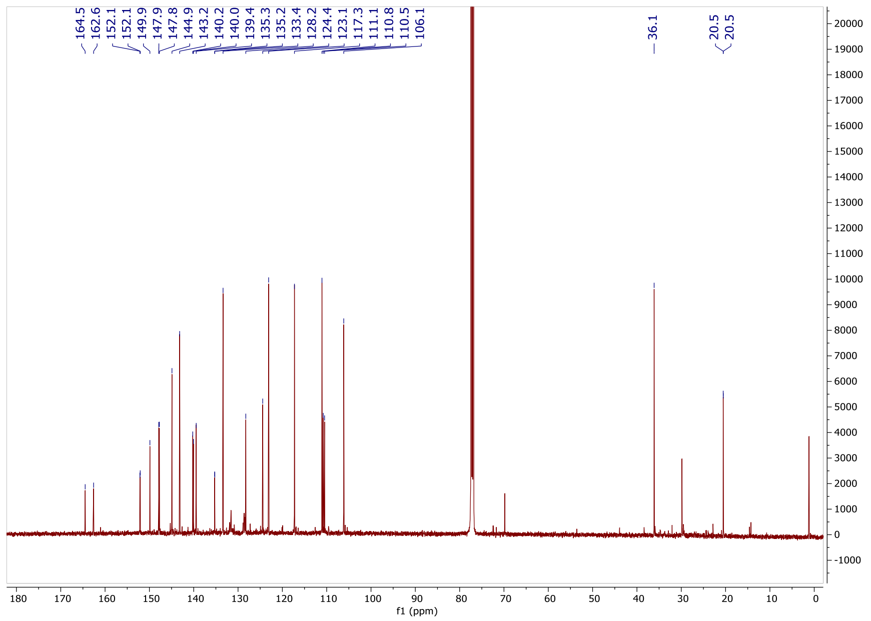

1-(1-Methyl-1*H*-indazol-5-yl)-6-(2-methylcyclohex-1-en-1-yl)-1*H*-imidazo[4,5-*c*]pyridine (9)

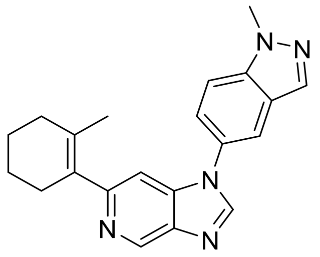

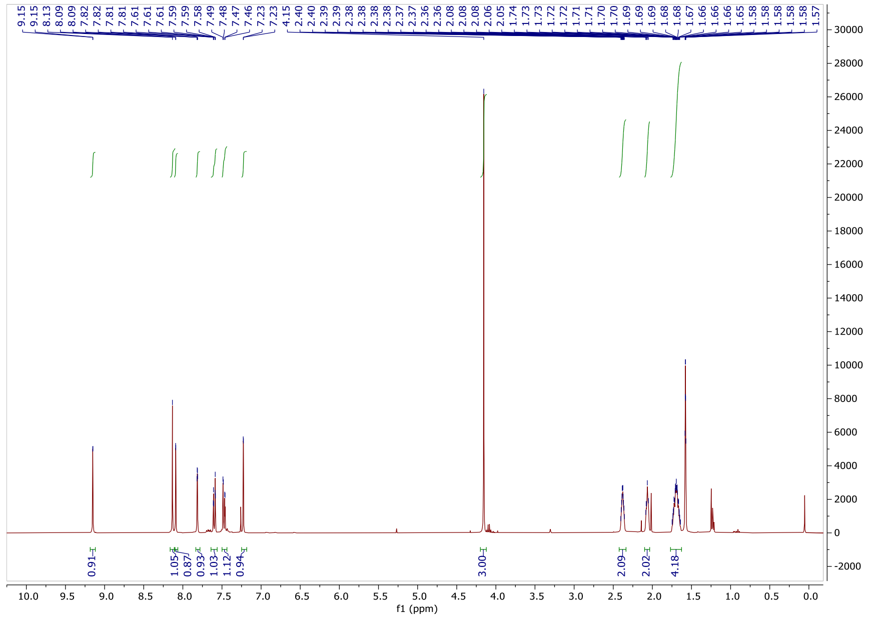

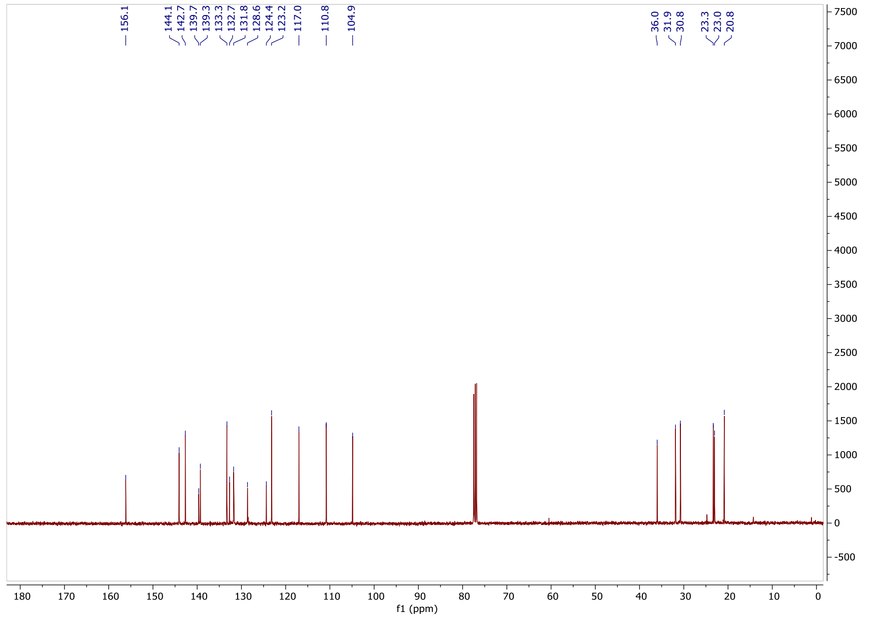

1-(1-Methyl-1*H*-indazol-5-yl)-6-(2-methylpiperidin-1-yl)-1*H*-imidazo[4,5-*c*]pyridine (10)

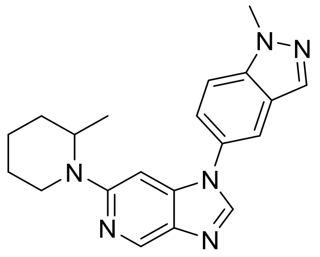

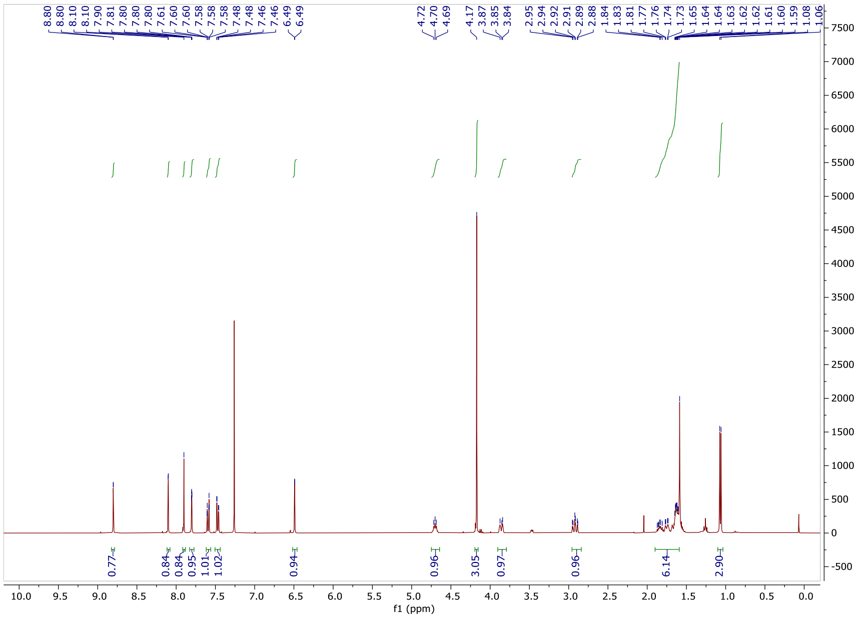

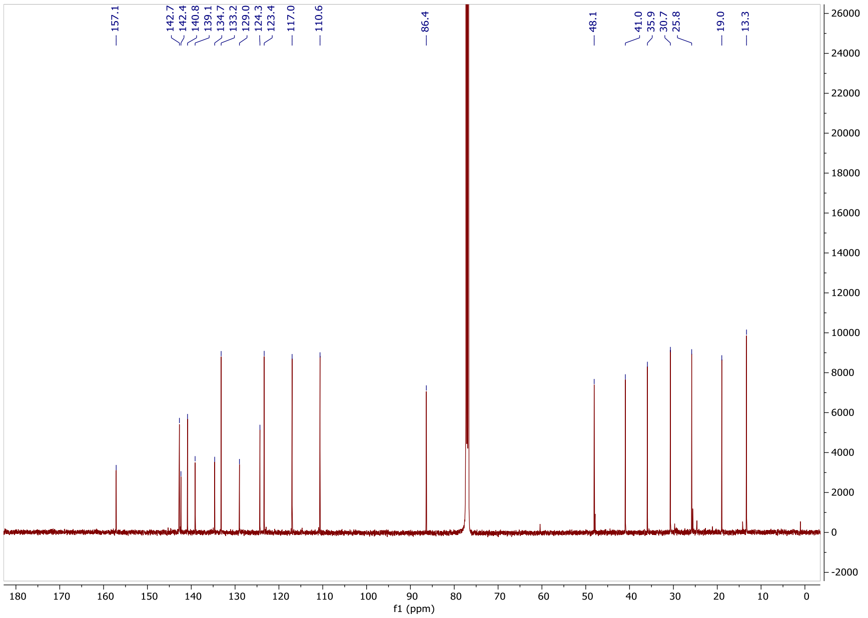

1-(1-(1-Methyl-1H-indazol-5-yl)-1H-imidazo[4,5-c]pyridin-6-yl)piperidin-2-one (11)

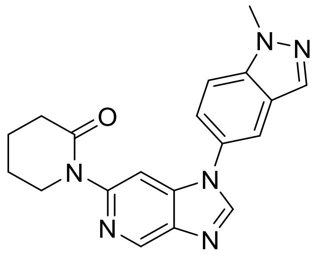

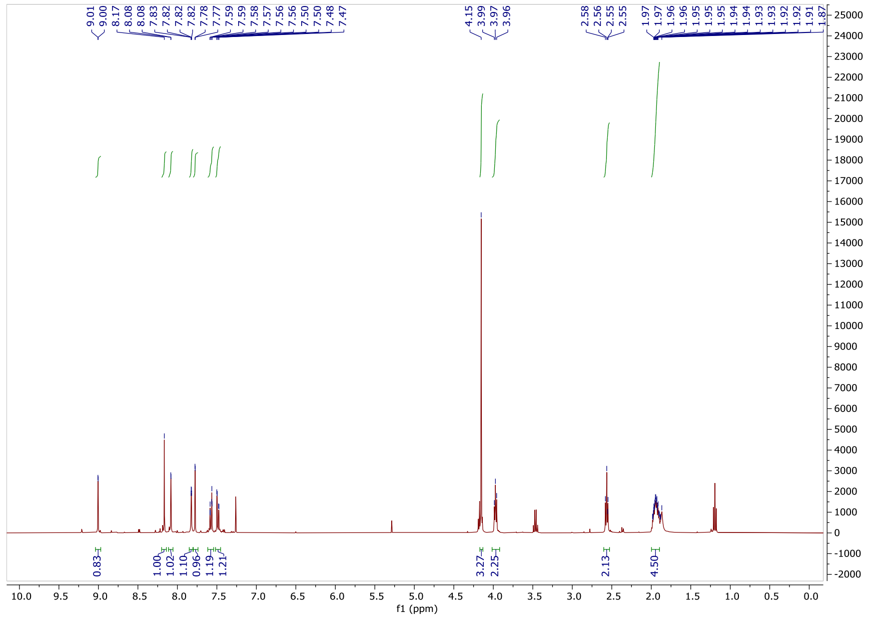

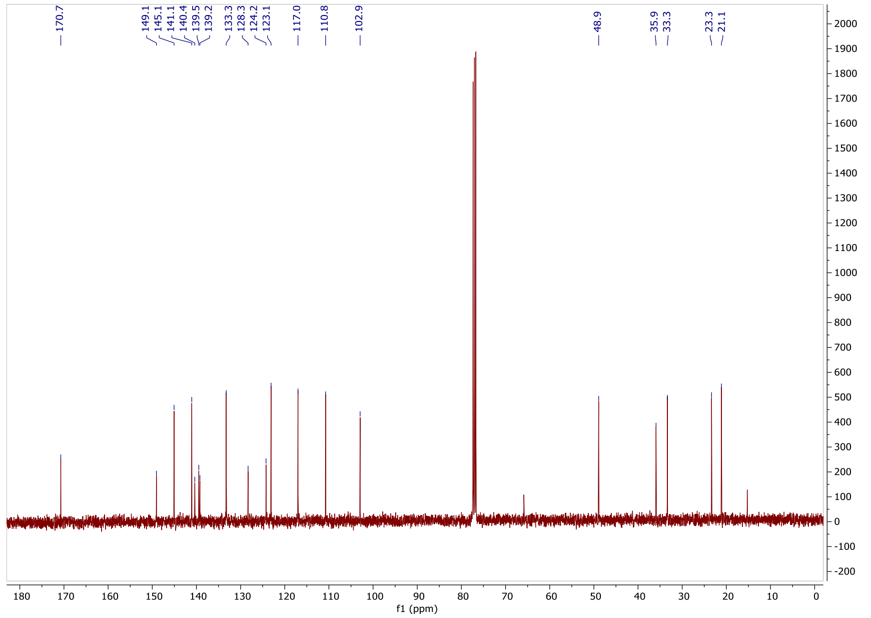

4-(1-(6-(Difluoromethoxy)pyridin-3-yl)-1*H*-imidazo[4,5-*c*]pyridin-6-yl)-3-methylbenzonitrile (14)

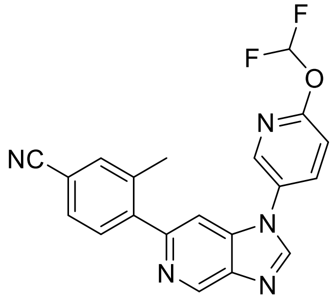

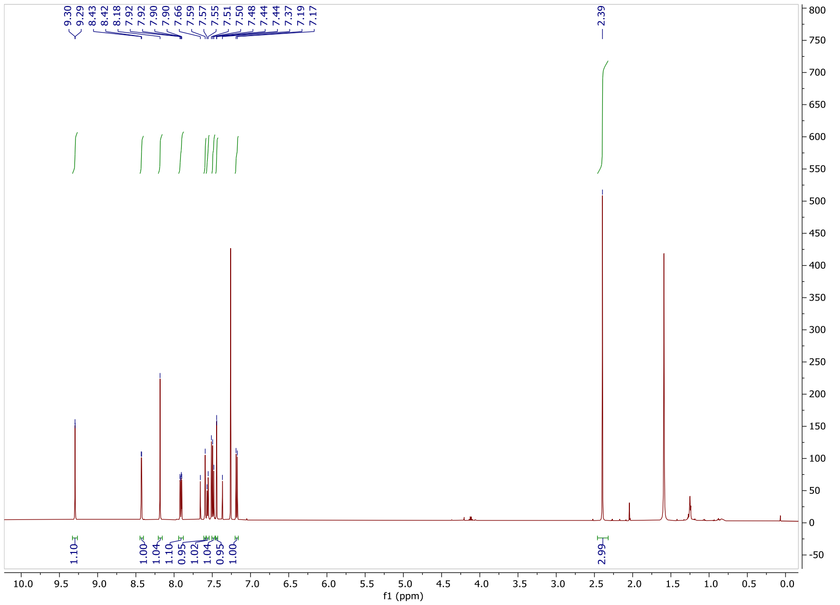

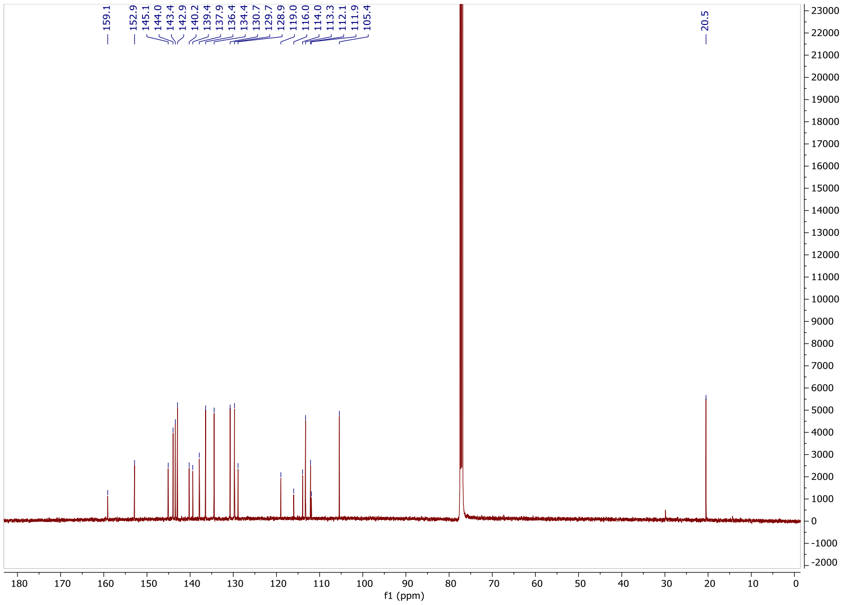

1-(1,3-Dimethyl-1*H*-indazol-5-yl)-6-(4-fluoro-2-methylphenyl)-1*H*-imidazo[4,5-*c*]pyridine (15)

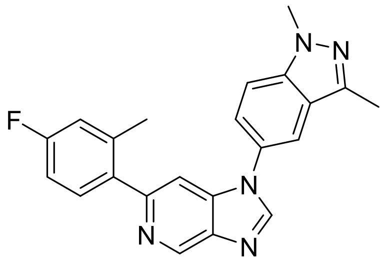

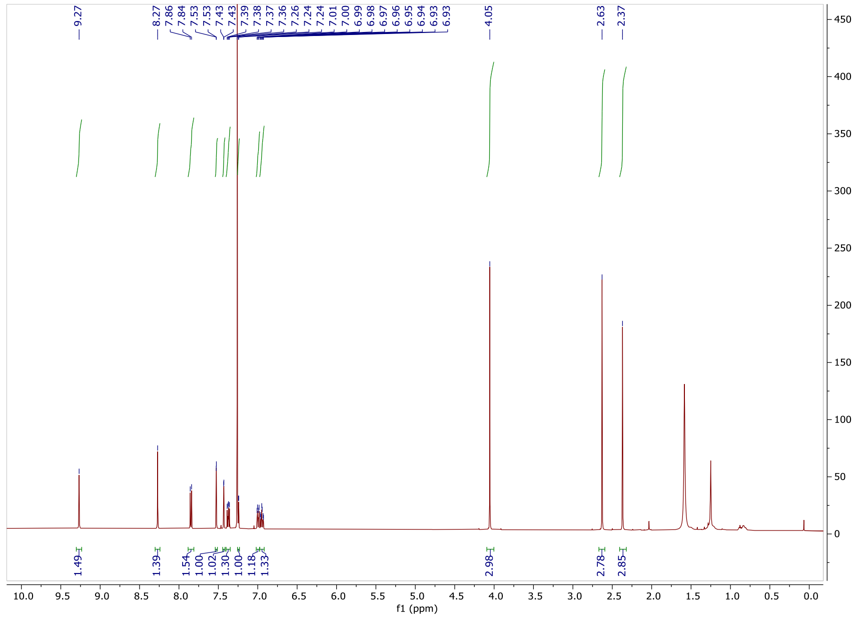

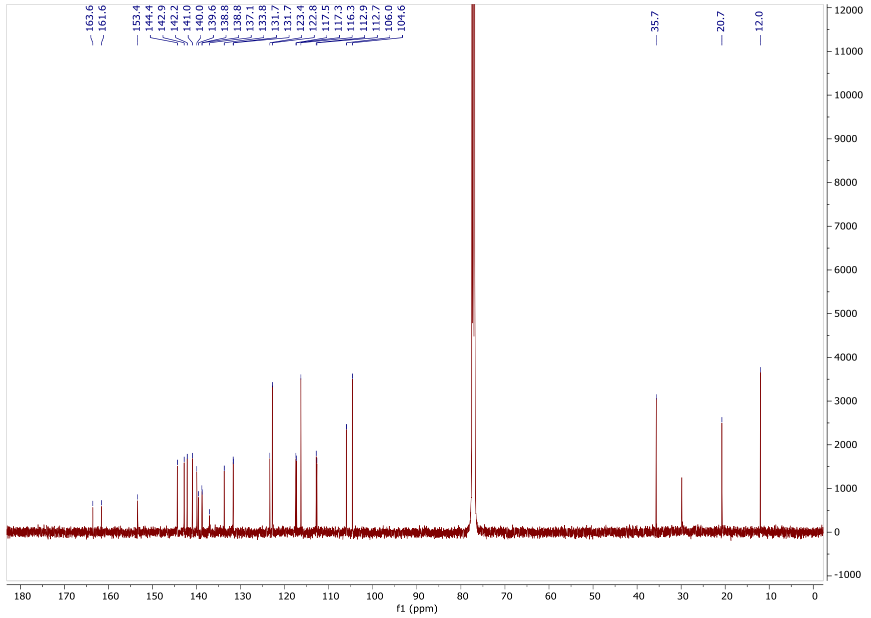

6-[6-(4-Fluoro-2-methylphenyl)-1*H*-imidazo[4,5-*c*]pyridin-1-yl]-1-methyl-1*H*-indazole (16)

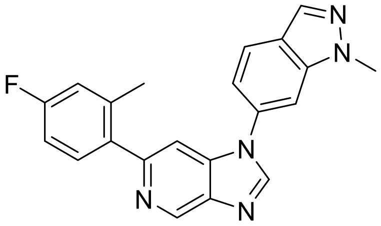

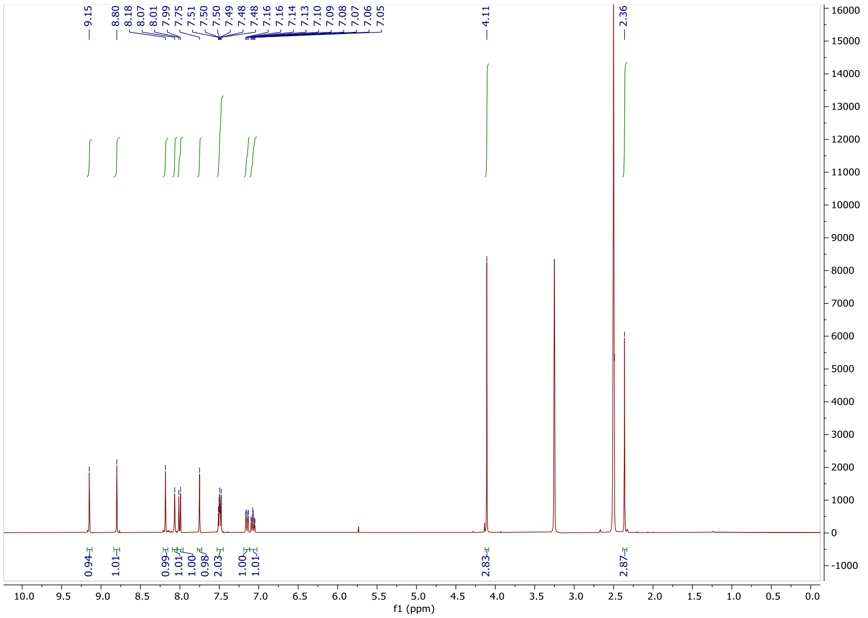

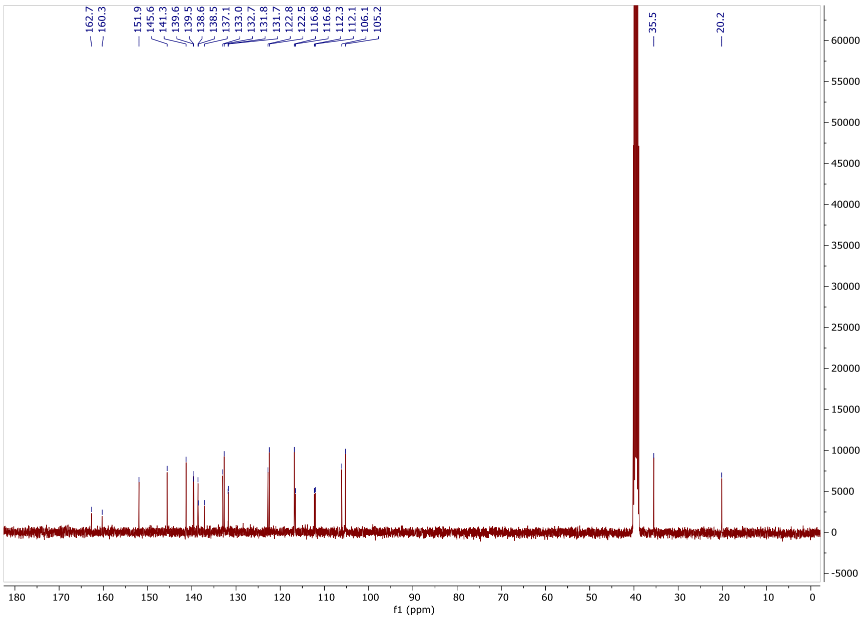

5-[6-(4-Fluoro-2-methylphenyl)-1*H*-imidazo[4,5-*c*]pyridin-1-yl]-3-methyl-1*H*-indazole (17)

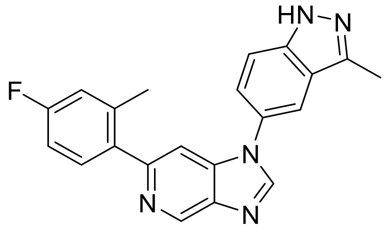

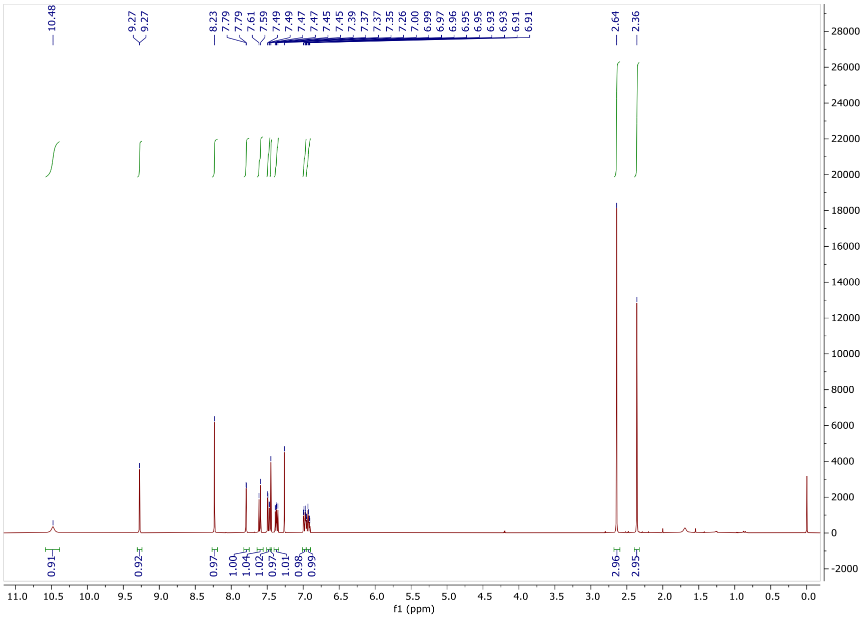

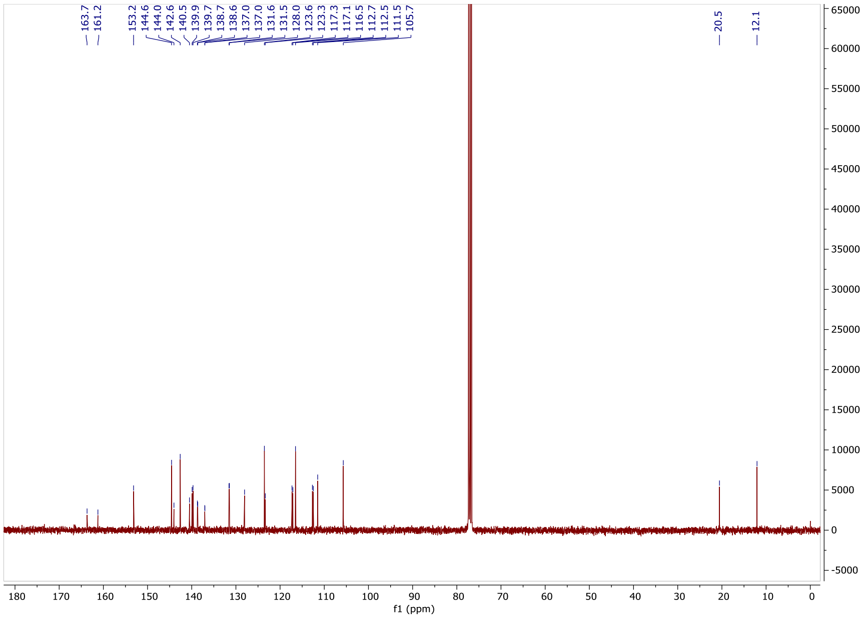

5-[6-(4-Fluoro-2-methylphenyl)-1*H*-imidazo[4,5-*c*]pyridin-1-yl]-1H-indazole (18)

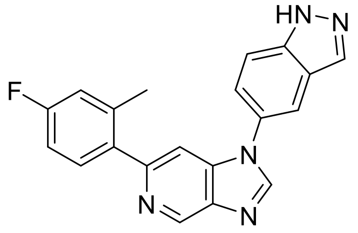

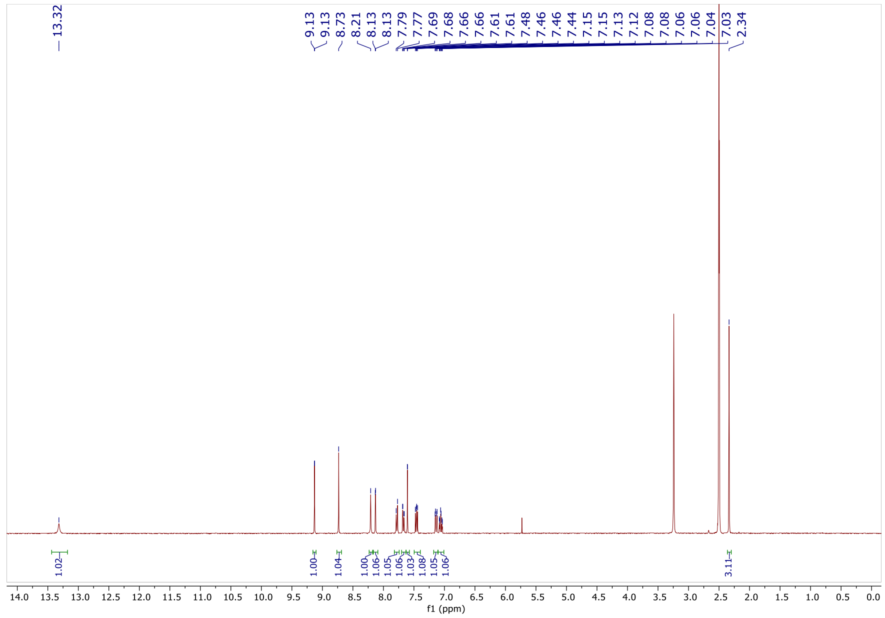

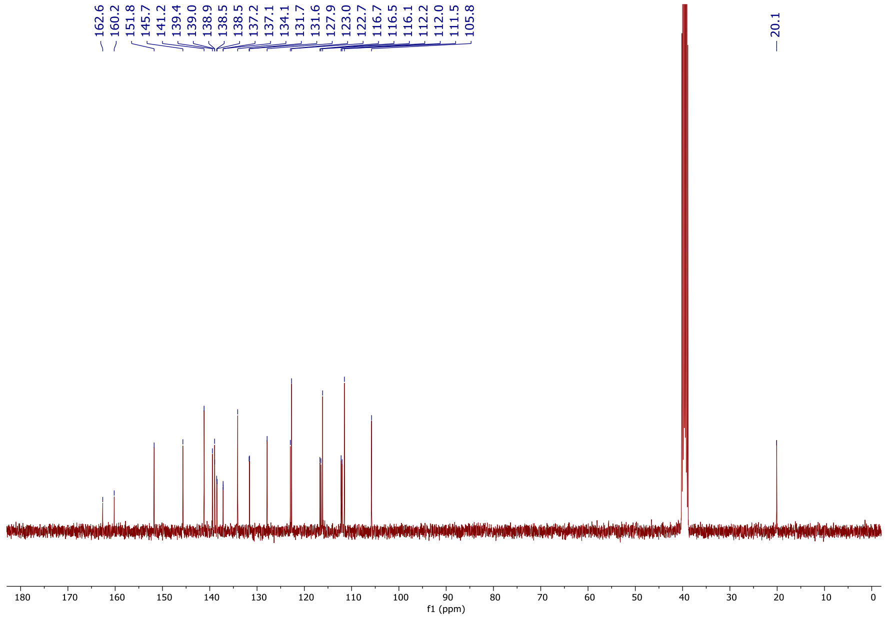

6-[6-(4-Fluoro-2-methylphenyl)-1*H*-imidazo[4,5-*c*]pyridin-1-yl]-3-methyl-1*H*-indazole (19)

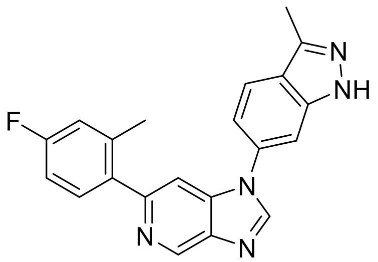

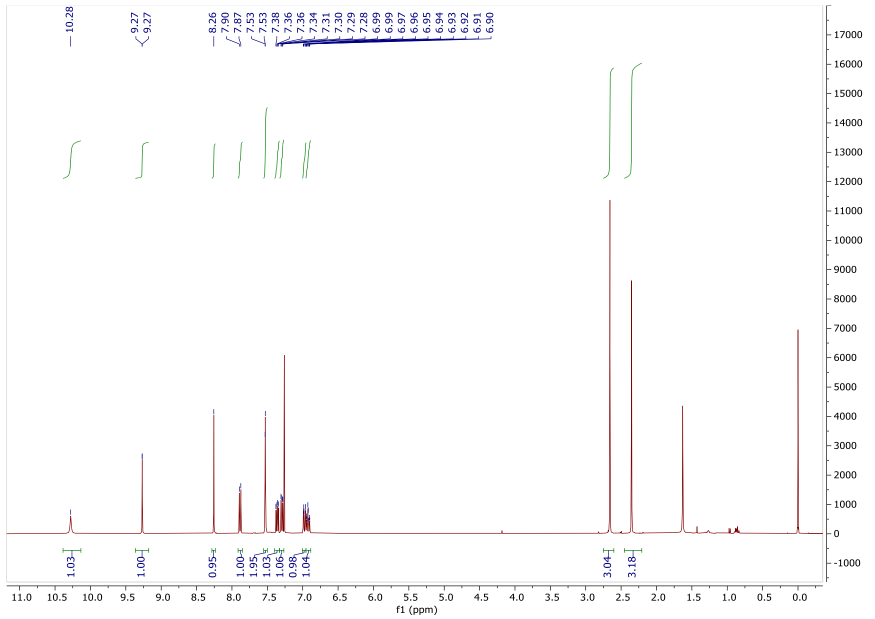

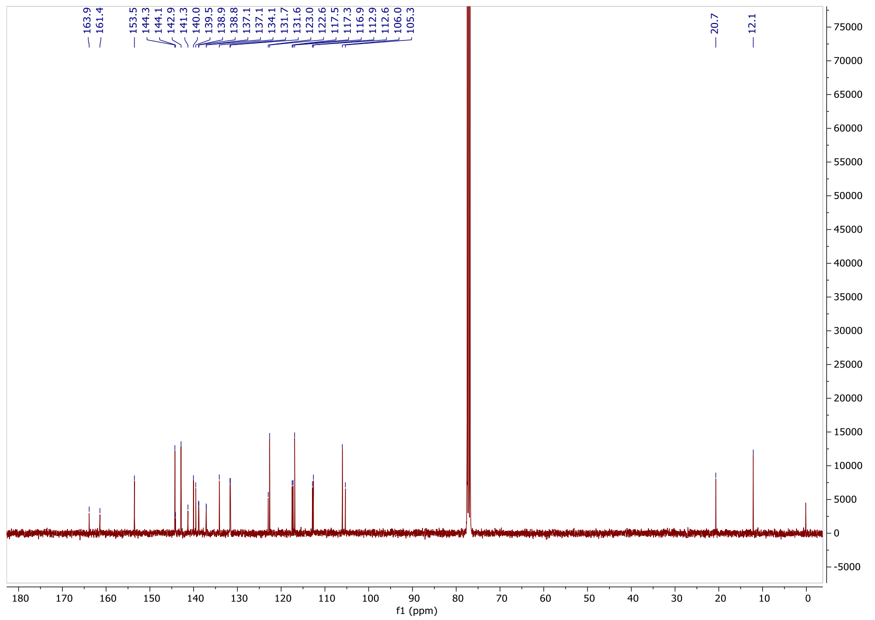

3-Methyl-4-[1-(3-methyl-1H-indazol-6-yl)imidazo[4,5-c]pyridin-6-yl]benzonitrile (OXS008255, 20)

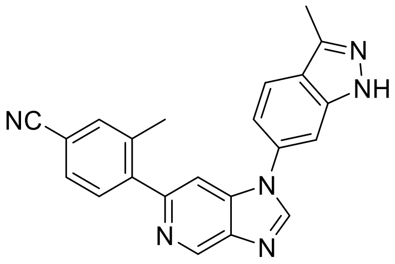

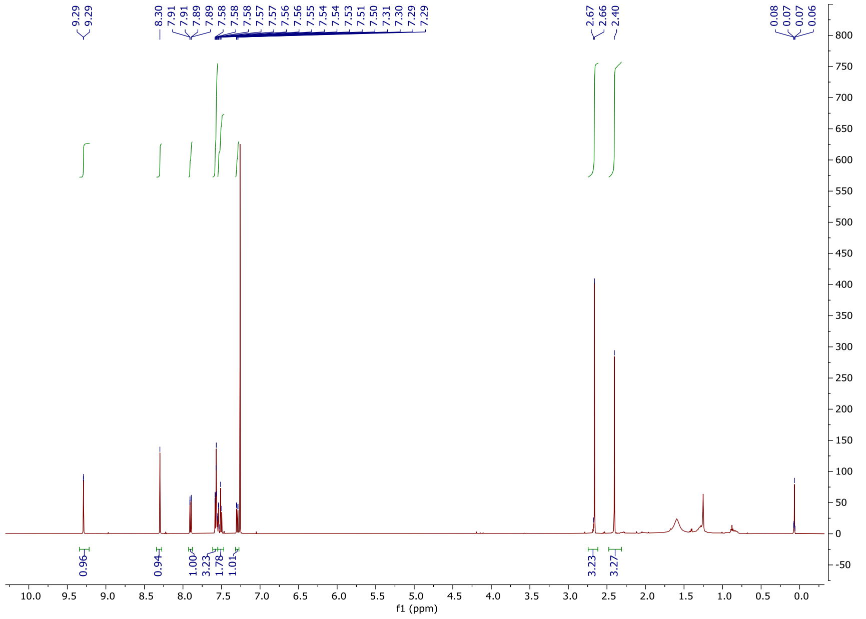

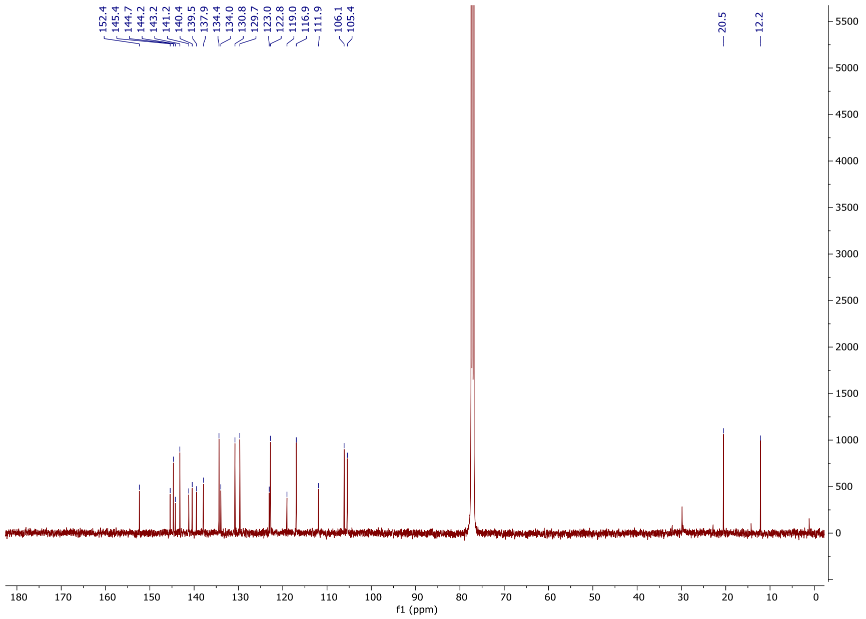

6-(6-(4-Fluoro-2-methylphenyl)-1*H*-imidazo[4,5-*c*]pyridin-1-yl)-2-methylbenzo[*d*]oxazole (21)

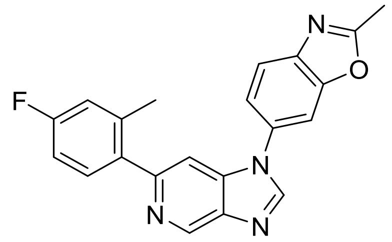

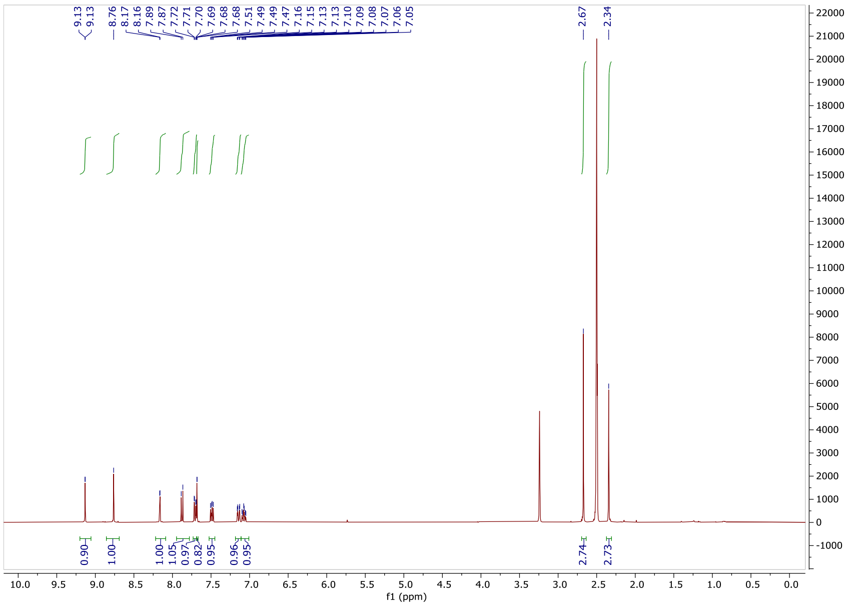

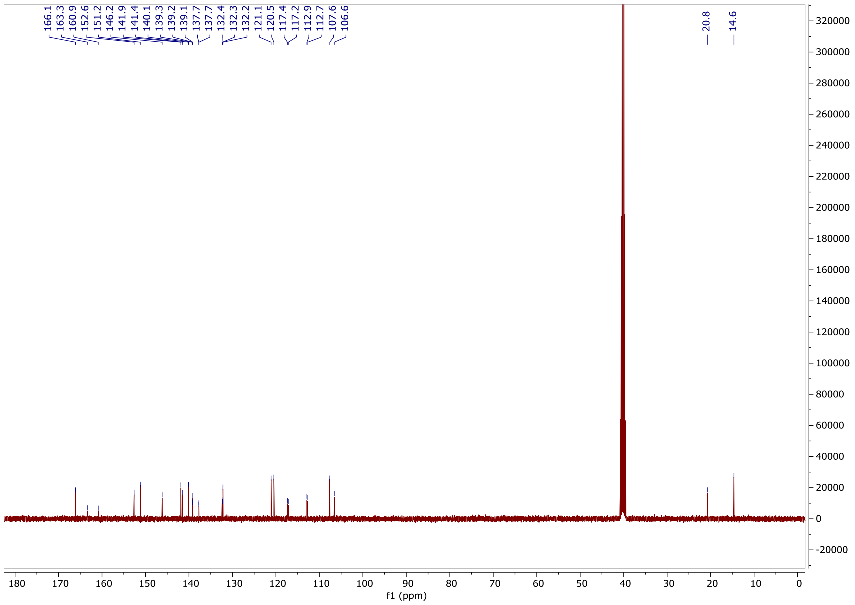

5-[6-(4-Fluoro-2-methylphenyl)-1*H*-imidazo[4,5-*c*]pyridin-1-yl]-2-methyl-1,3-benzoxazole (22)

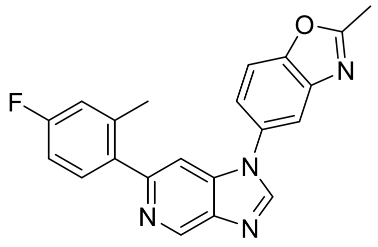

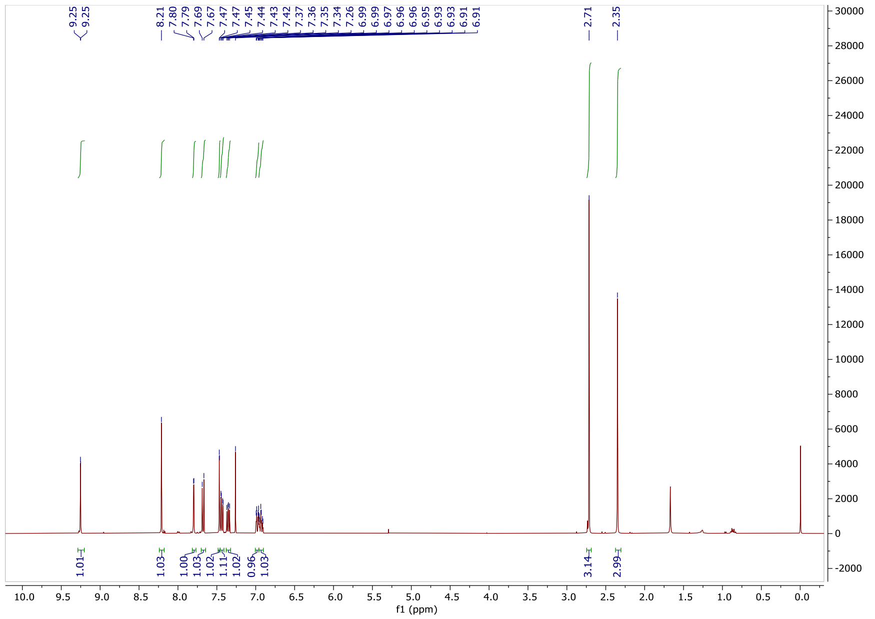

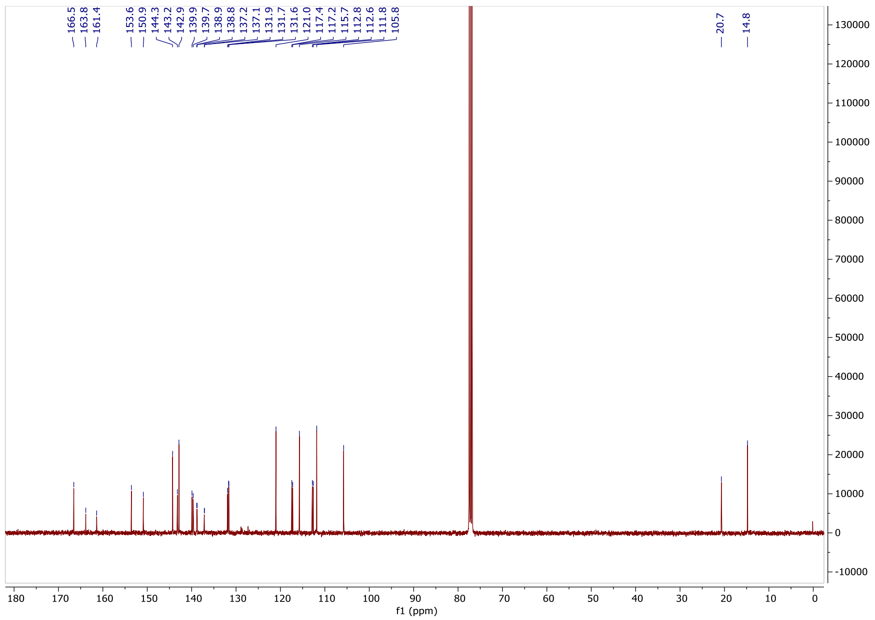

6-(4-Fluoro-2-methyl-phenyl)-1-(2-methylindazol-6-yl)imidazo[4,5-*c*]pyridine (23)

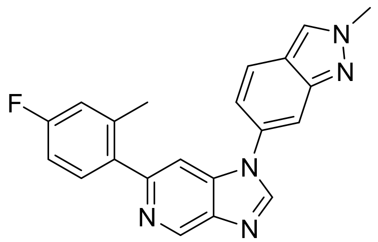

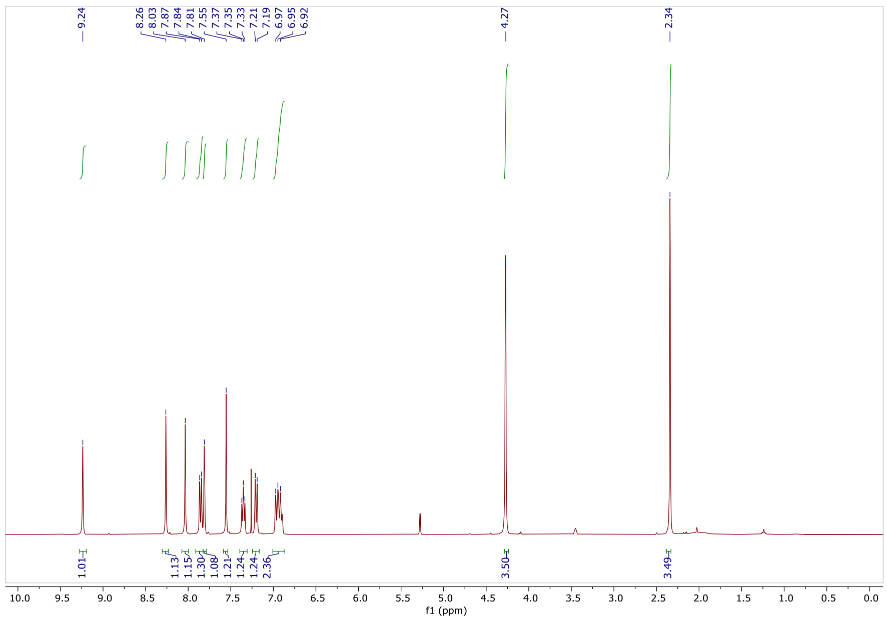

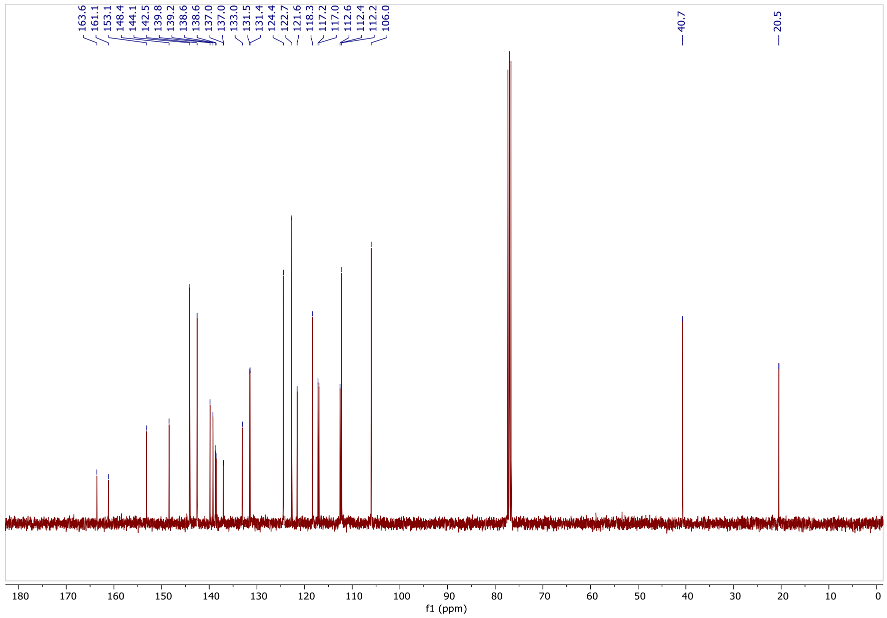

1-(2,3-Dimethyl-2*H*-indazol-5-yl)-6-(4-fluoro-2-methylphenyl)-1*H*-imidazo[4,5-*c*]pyridine (24)

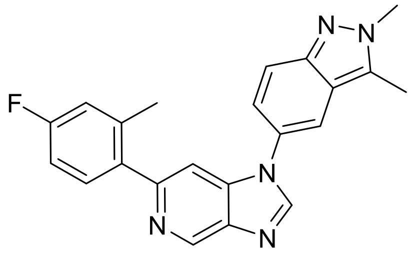

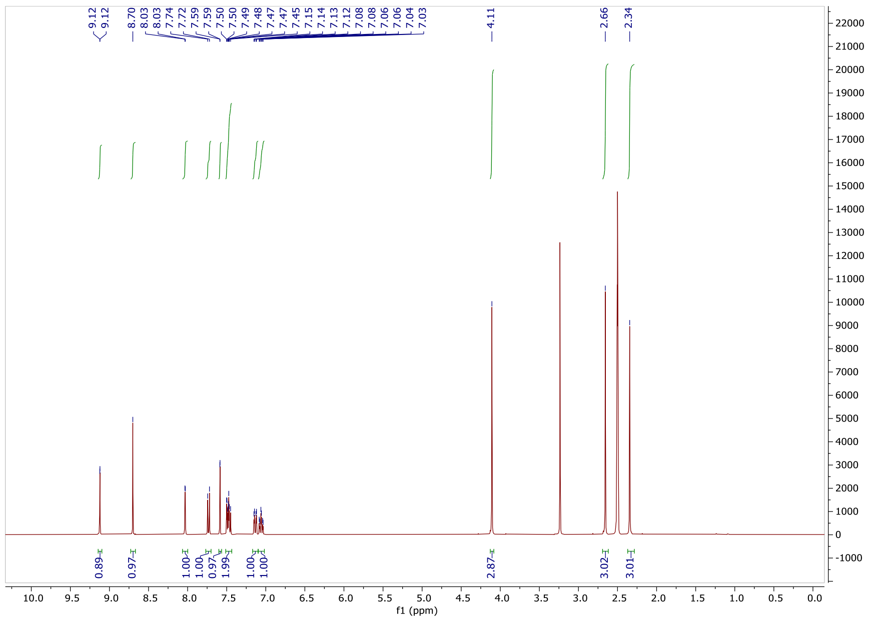

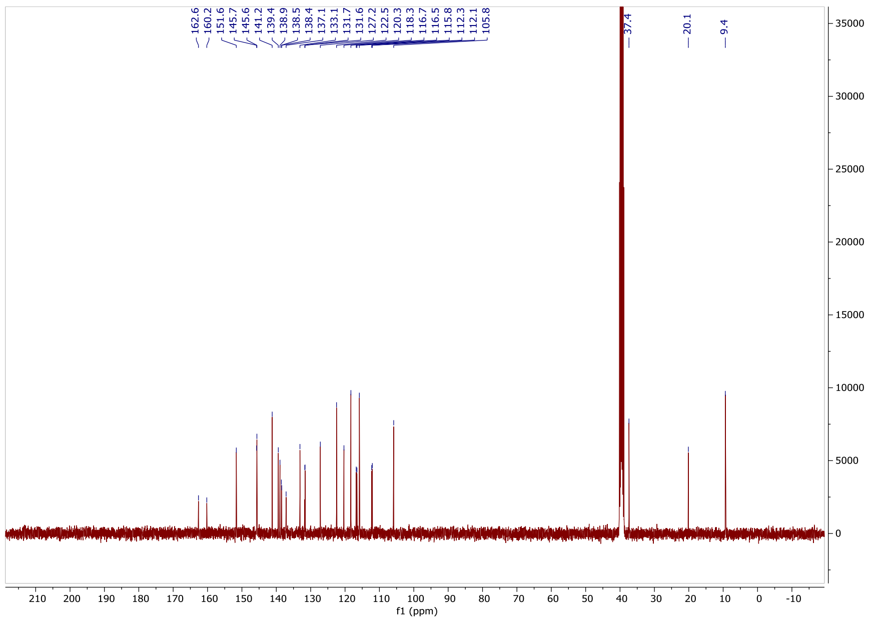

5-(6-(4-Fluoro-2-methylphenyl)-1*H*-imidazo[4,5-*c*]pyridin-1-yl)-1-methyl-1*H*-pyrazolo[4,3-*b*]pyridine (25)

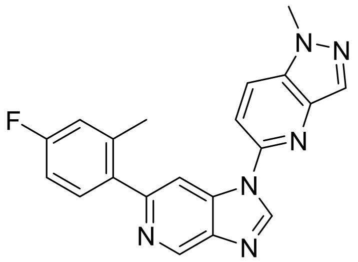

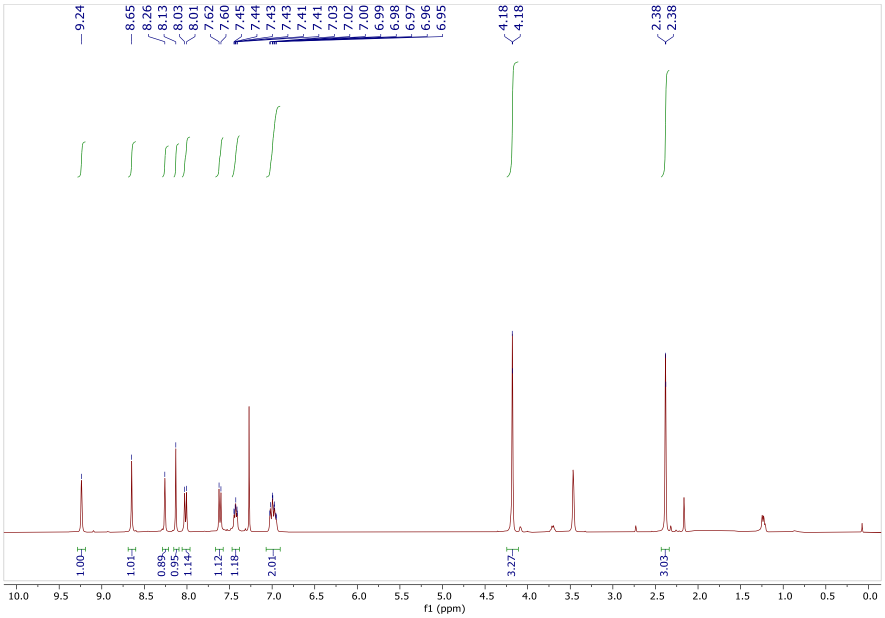

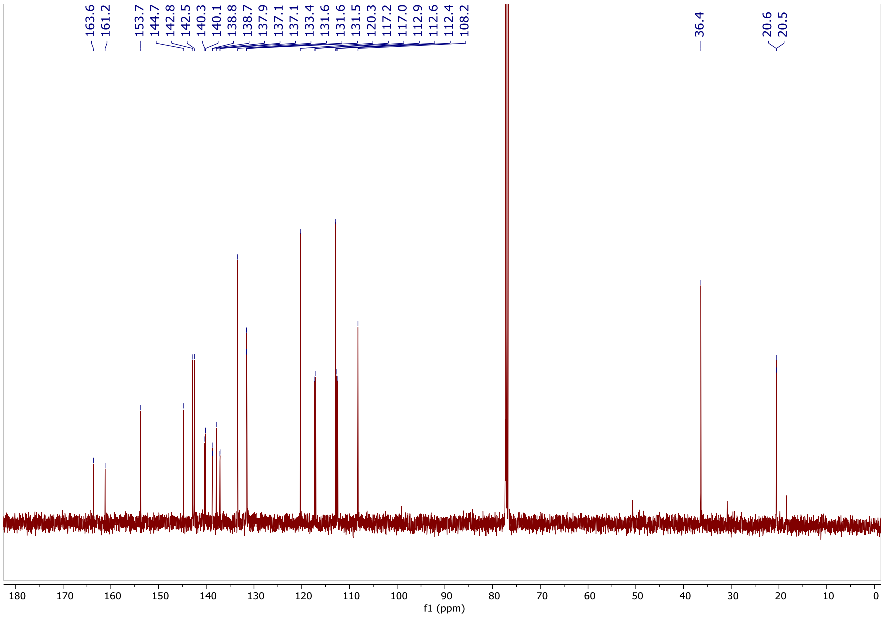

5-(6-(4-Fluoro-2-methylphenyl)-1*H*-imidazo[4,5-*c*]pyridin-1-yl)-1-methyl-1*H*-pyrazolo[3,4-*c*]pyridine (26)

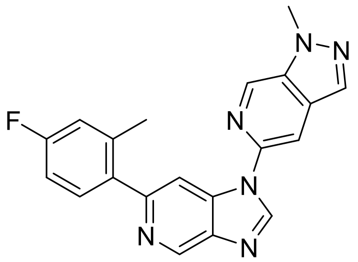

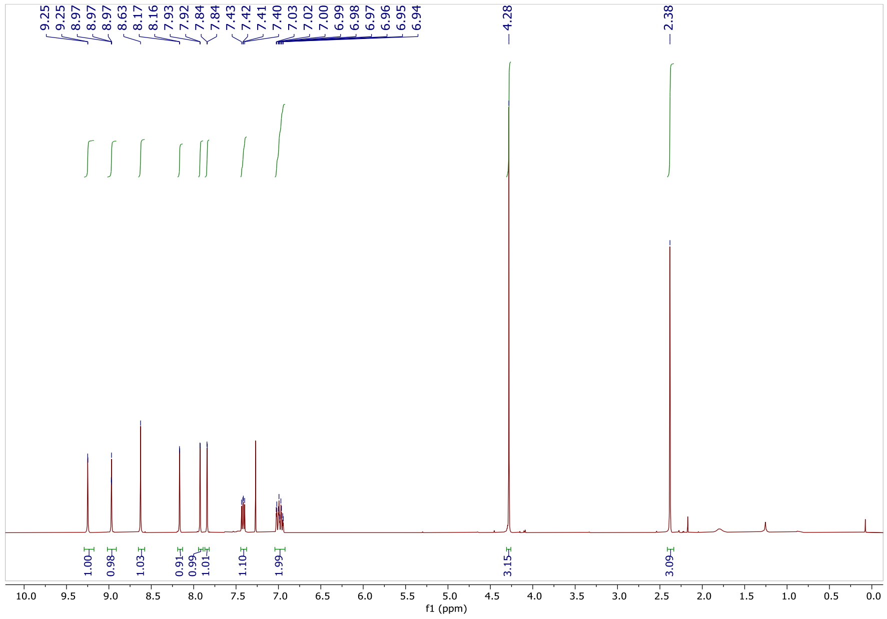

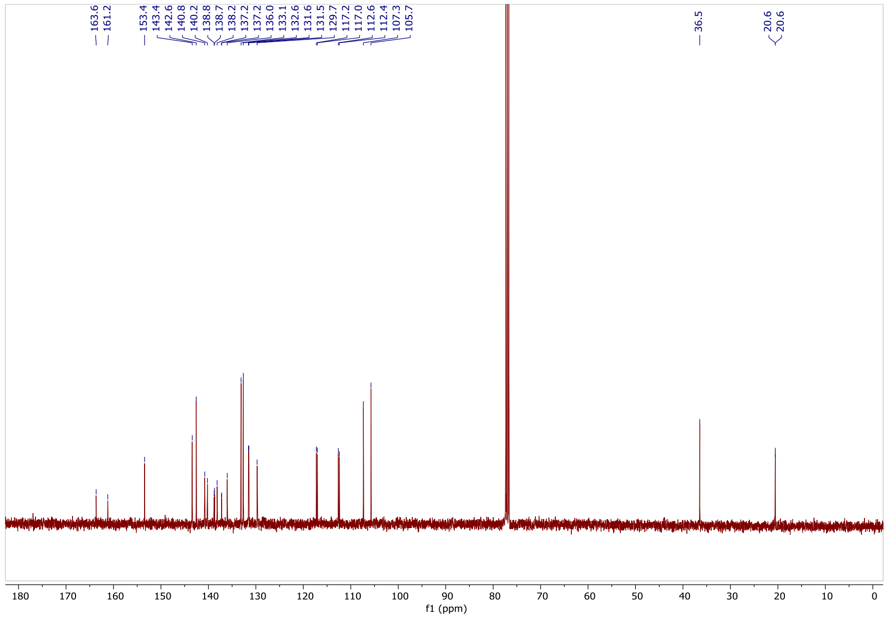

6-(4-Fluoro-2-methylphenyl)-1-{1-methyl-1*H*-pyrazolo[3,4-*b*]pyridin-5-yl}-1*H*-imidazo[4,5-*c*]pyridine (OXS008203, 27)

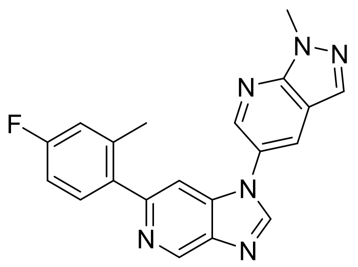

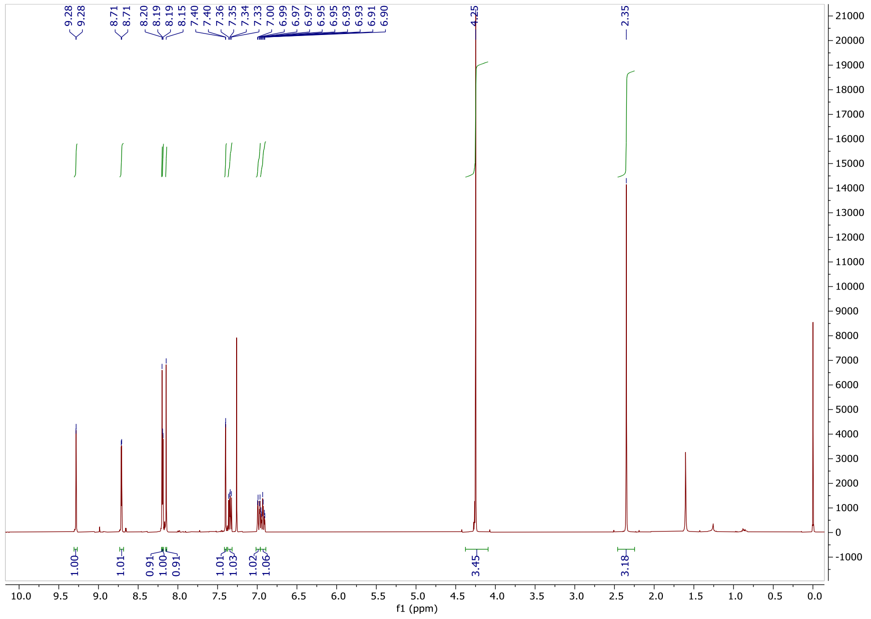

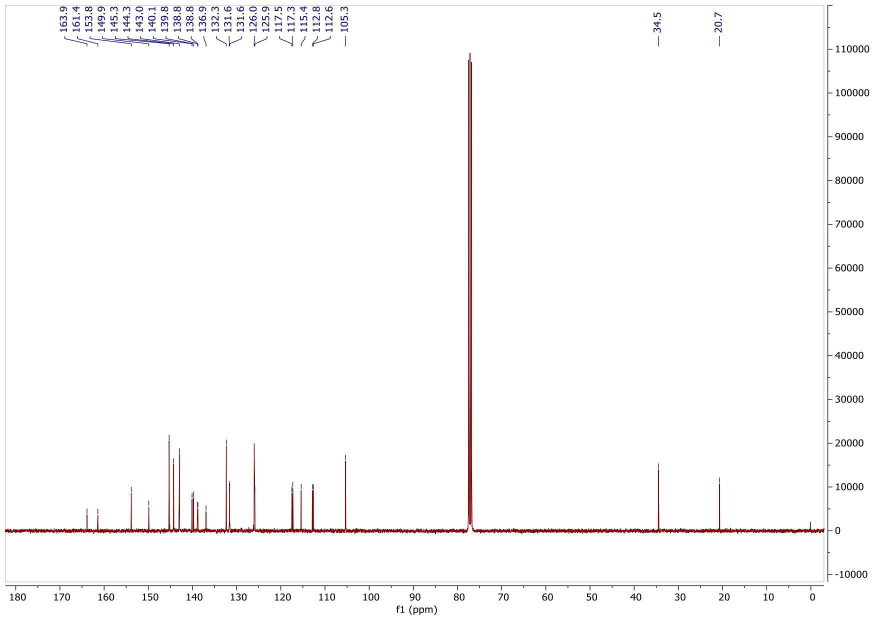

6-(6-Fluoro-4-methyl-3-pyridyl)-1-(2-methylindazol-6-yl)imidazo[4,5-*c*]pyridine (28)

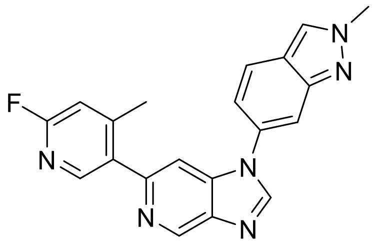

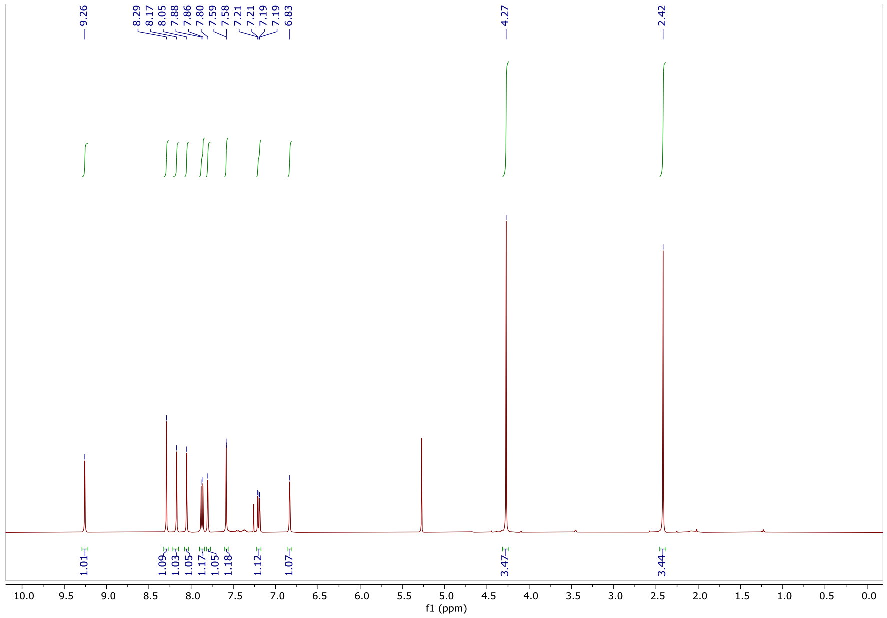

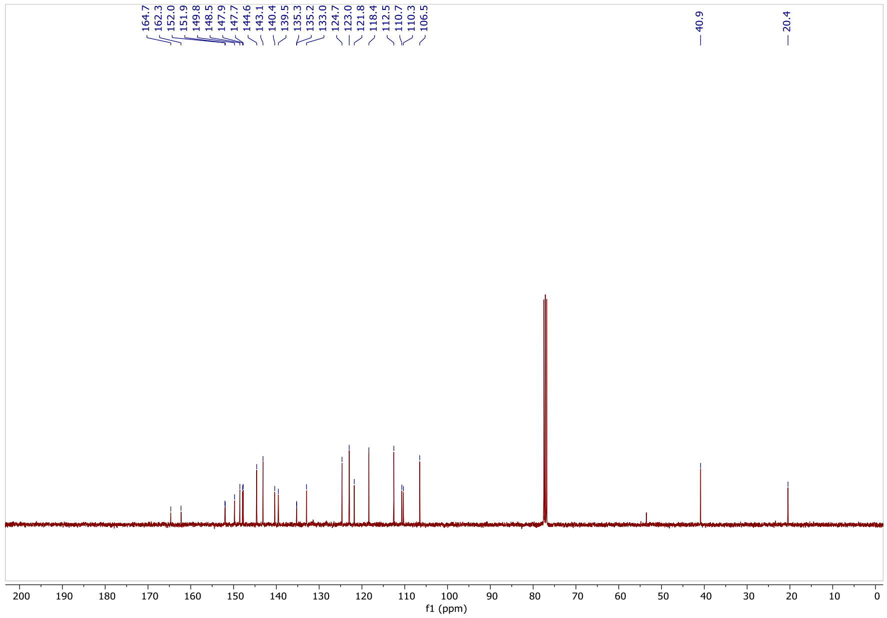

5-(6-(6-Fluoro-4-methylpyridin-3-yl)-1*H*-imidazo[4,5-*c*]pyridin-1-yl)-1-methyl-1*H*-pyrazolo[4,3-*b*]pyridine (29)

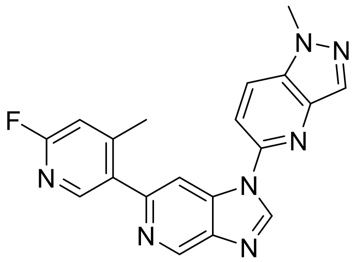

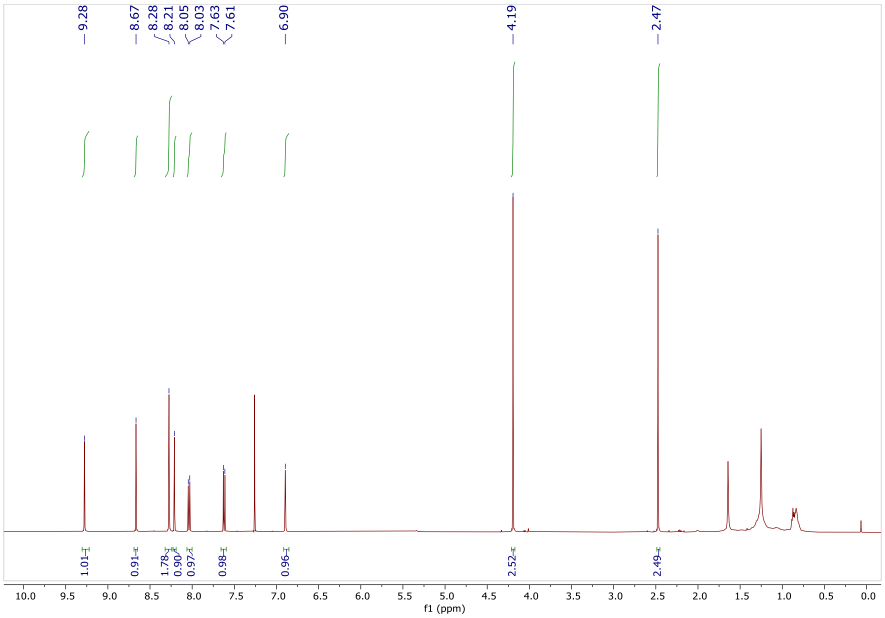

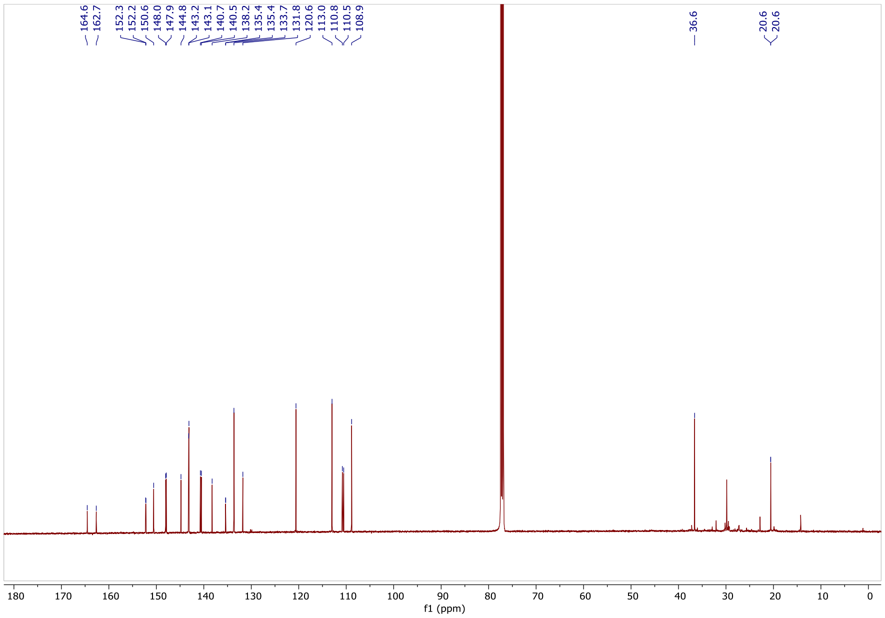

5-(6-(6-Fluoro-4-methylpyridin-3-yl)-1*H*-imidazo[4,5-*c*]pyridin-1-yl)-1-methyl-1*H*-pyrazolo[3,4-*b*]pyridine (30)

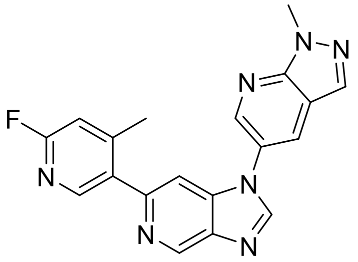

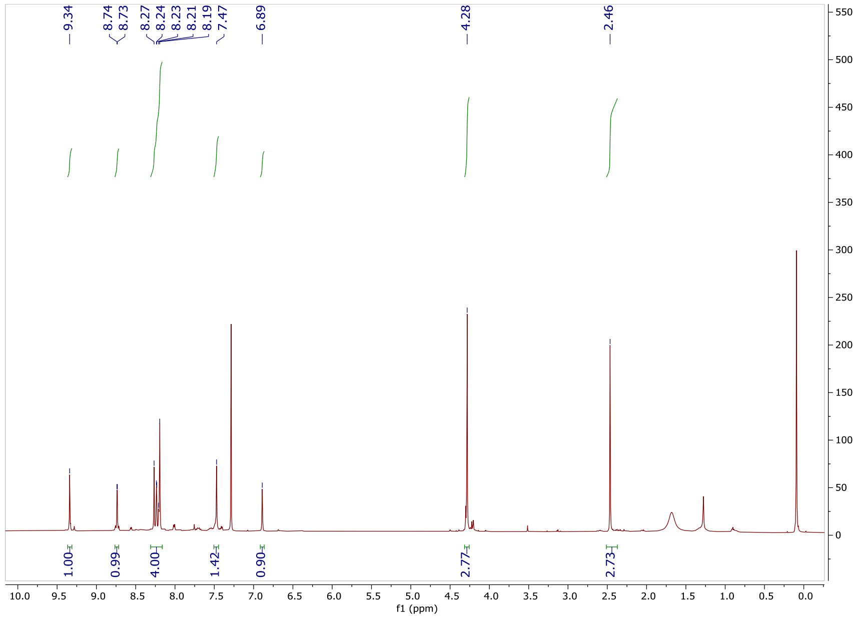

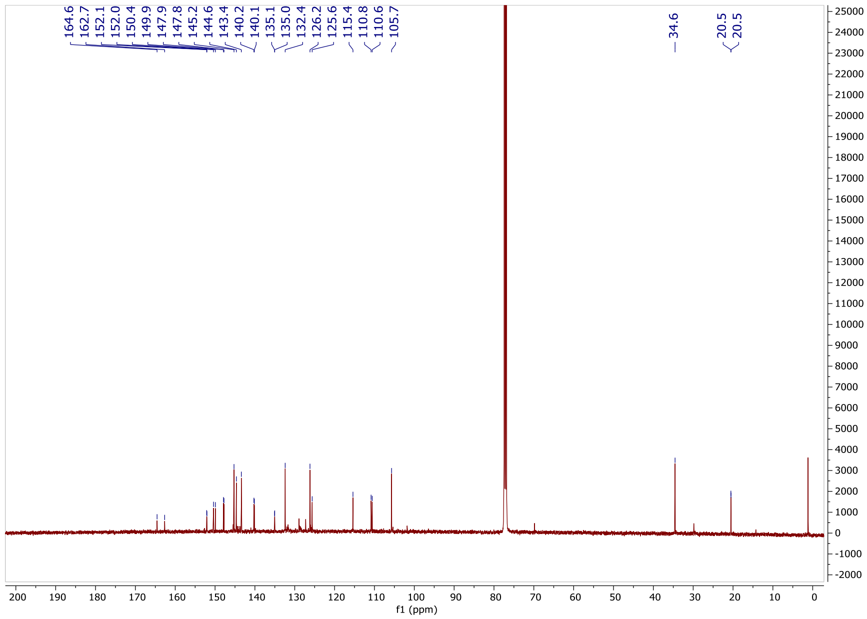

5-(6-(6-Fluoro-4-methylpyridin-3-yl)-1*H*-imidazo[4,5-*c*]pyridin-1-yl)-1-methyl-1*H*-pyrazolo[3,4-*c*]pyridine (31)

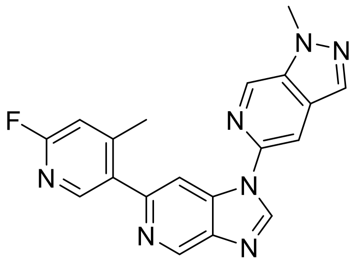

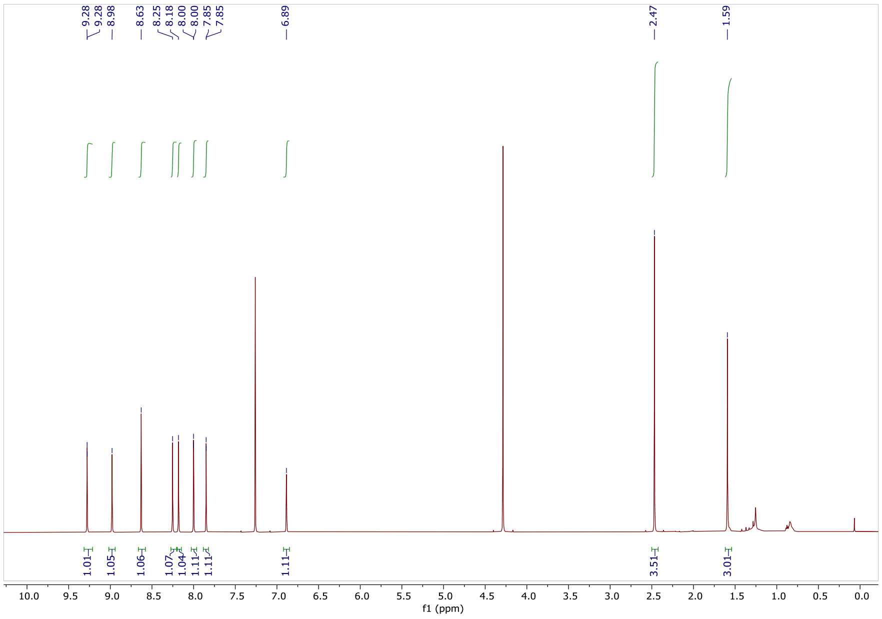

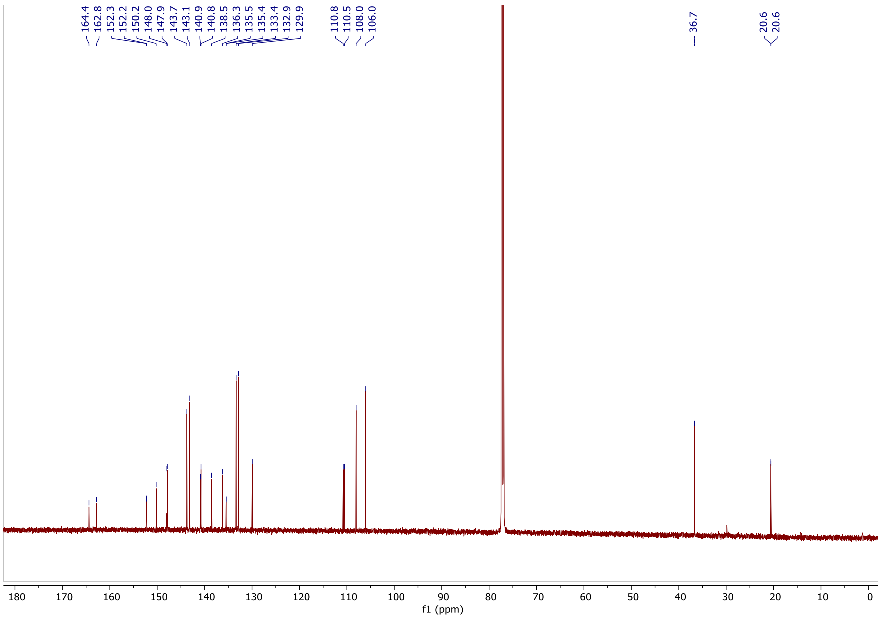

5-(6-(6-Fluoro-4-methylpyridin-3-yl)-1*H*-imidazo[4,5-*c*]pyridin-1-yl)-2-methyl-2*H*-pyrazolo[3,4-*c*]pyridine (32)

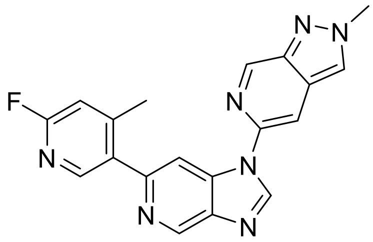

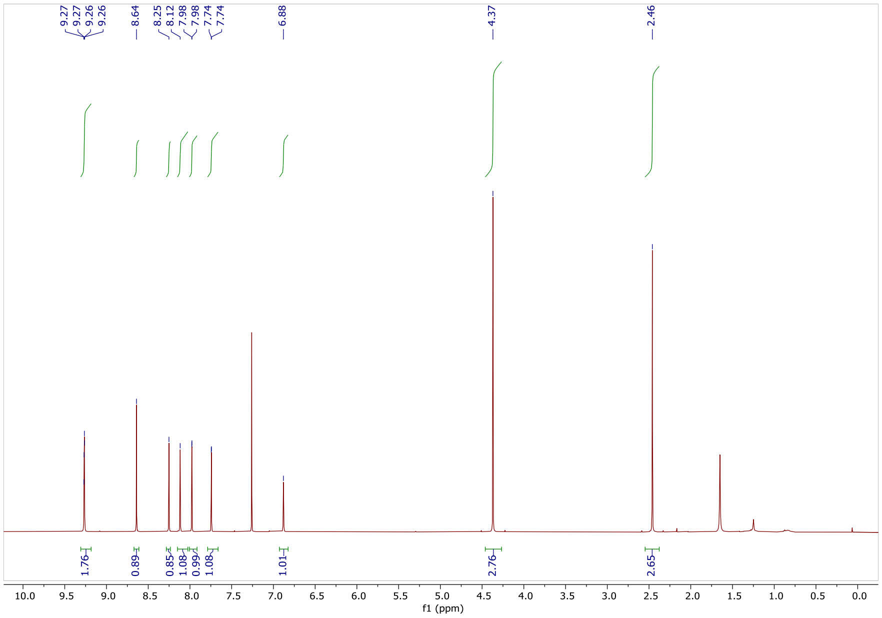

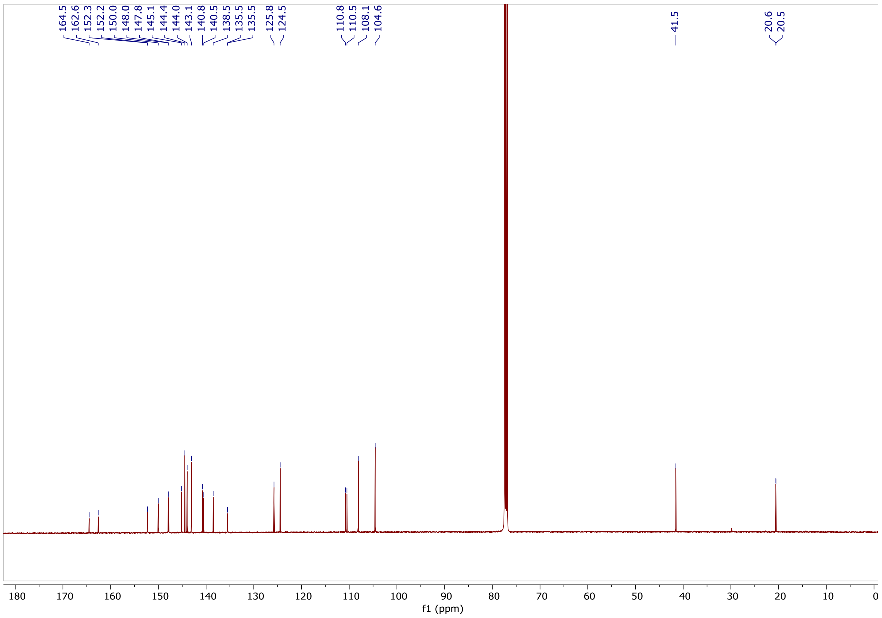

6-(6-Fluoro-4-methylpyridin-3-yl)-1-(3-methyl-1*H*-indazol-6-yl)-1*H*-imidazo[4,5-*c*]pyridine (33)

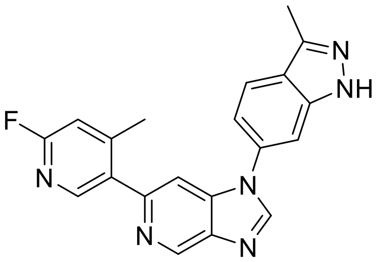

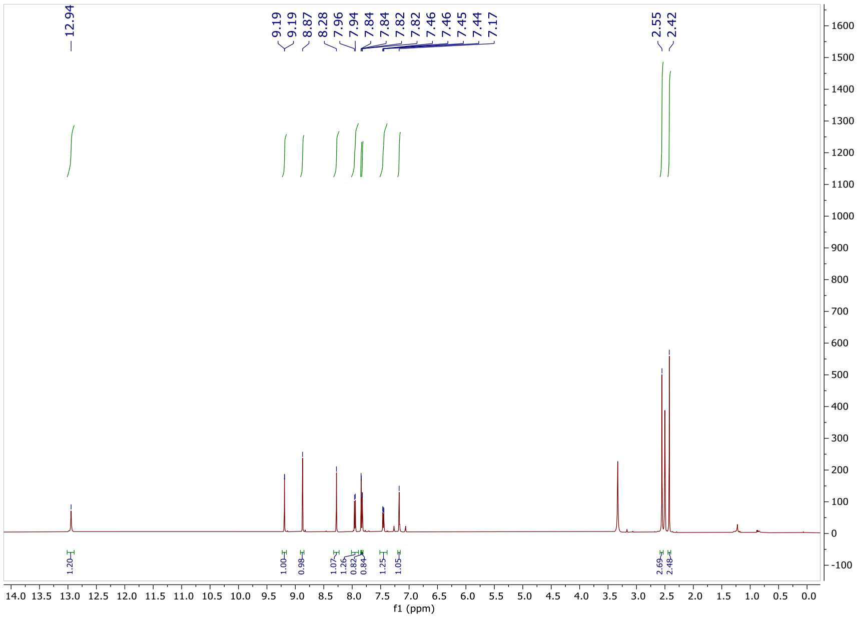

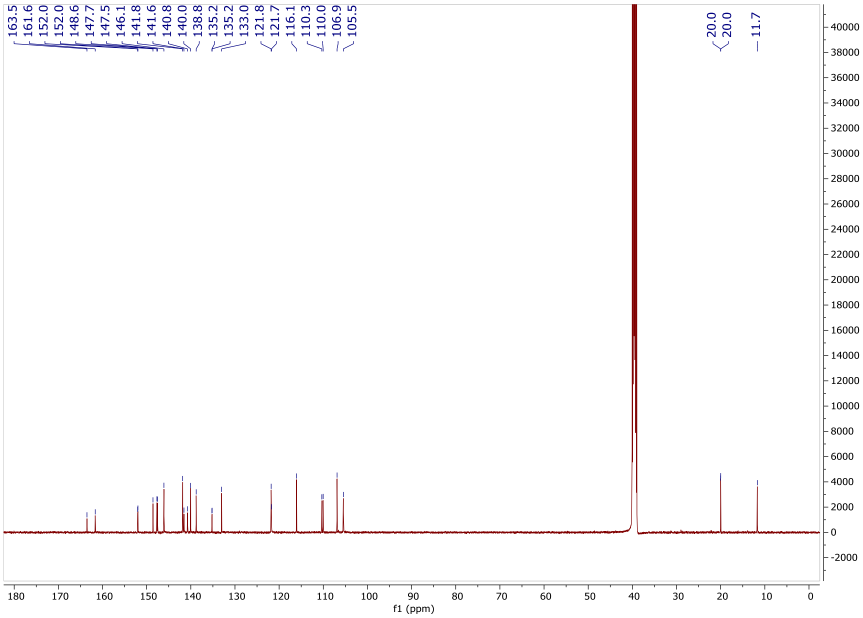

6-(6-(6-Fluoro-4-methylpyridin-3-yl)-1*H*-imidazo[4,5-*c*]pyridin-1-yl)-3-methyl-1*H*-pyrazolo[4,3-*b*]pyridine (34)

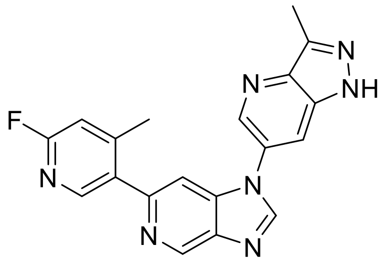

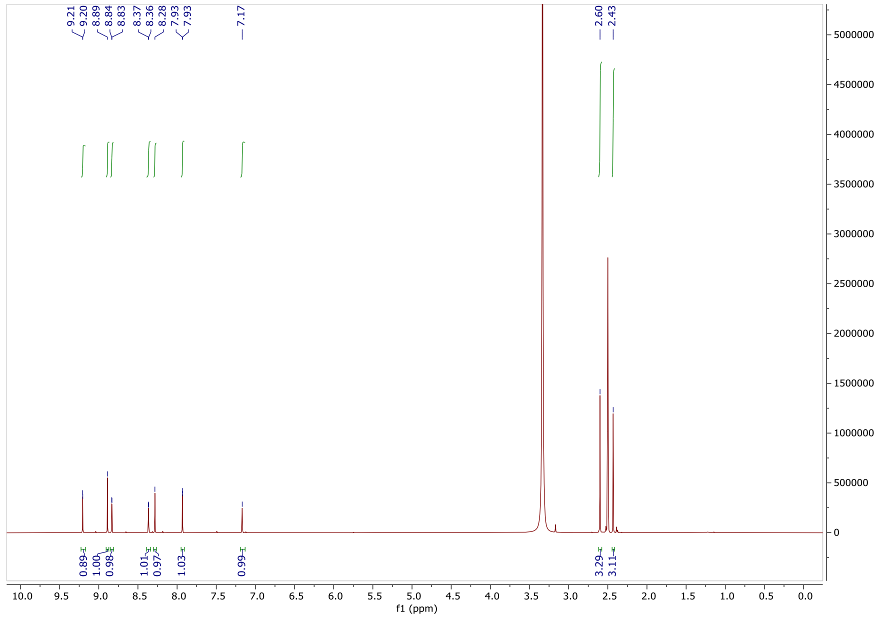

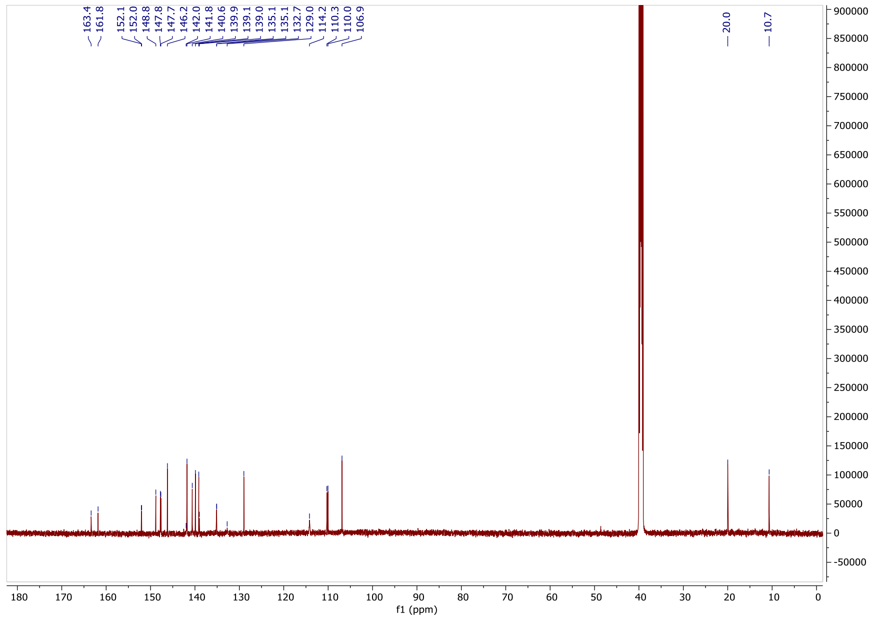

6-(6-(6-Fluoro-4-methylpyridin-3-yl)-1*H*-imidazo[4,5-*c*]pyridin-1-yl)-3-methylbenzo[*d*]isoxazole (35)

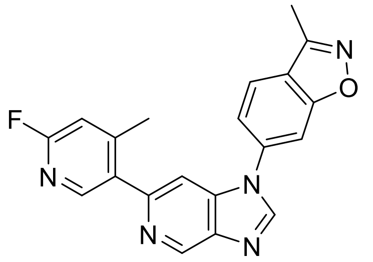

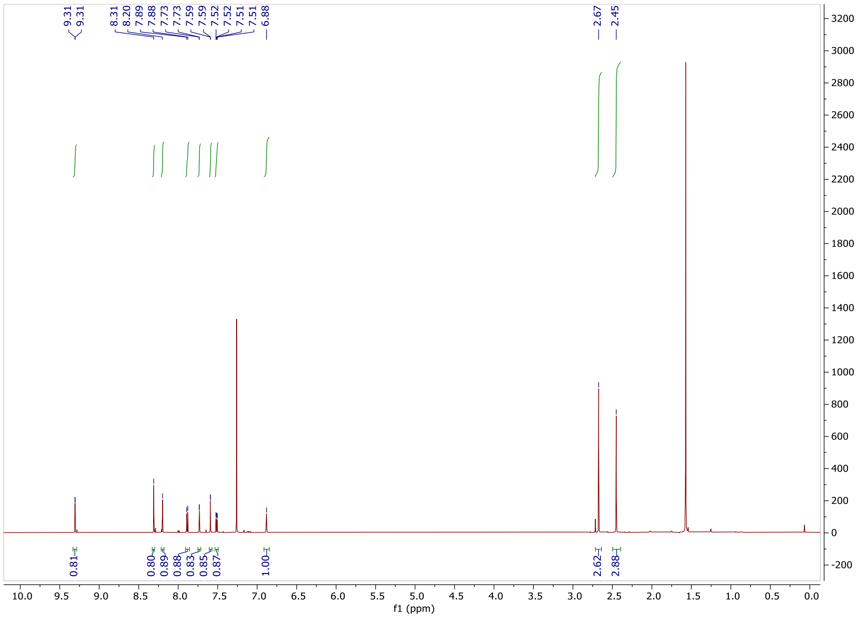

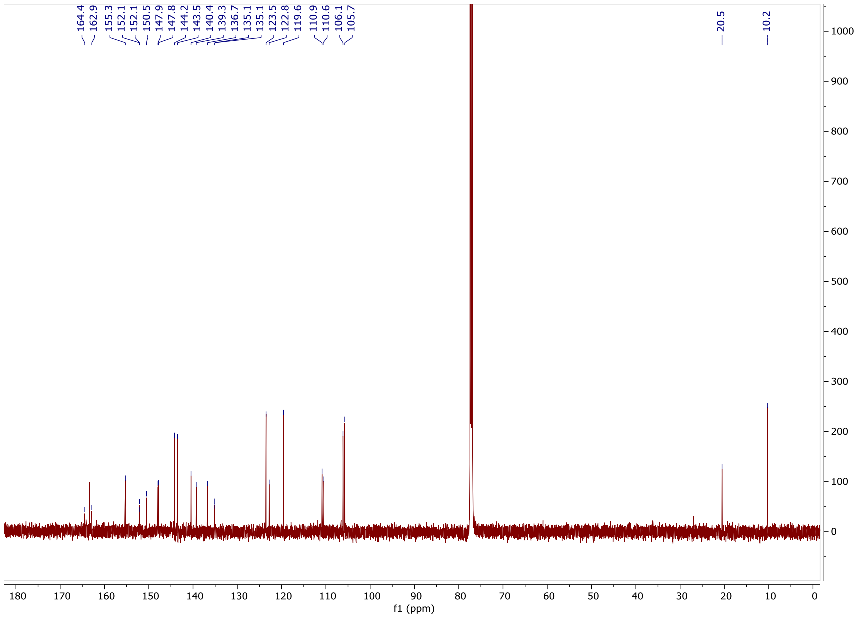

References

(1) Josa-Culleré, L.; Madden, K. S.; Cogswell, T. J.; Jackson, T. R.; Carter, T. S.; Zhang, D.; Trevitt, G.; Davies, S. G.; Vyas, P.; Wynne, G. M.; Milne, T. A.; Russell, A. J. A Phenotypic Screen Identifies a Compound Series That Induces Differentiation of Acute Myeloid Leukemia Cells in Vitro and Shows Anti-Tumour Effects in Vivo. *J Med Chem* **2021**, *64* (21), 15608–15628. https://doi.org/10.1021/acs.jmedchem.1c00574.
